# The impact of working from home on sedentary behaviour and physical activity compared to onsite work in the working population: a systematic review and meta-analysis

**DOI:** 10.1186/s12889-025-24960-x

**Published:** 2025-11-17

**Authors:** Carina Schöne, Martha Sauter, Eva-Maria Backé, Michaela Prigge, Claudia Brendler, Janice Hegewald

**Affiliations:** 1 Unit 3.1 Prevention of Work-Related Diseases, Federal Institute for Occupational Safety and Health, Nöldnerstr. 40-42, Berlin, 10317 Germany; 2 Unit 3.4 Medical Occupational Safety and Health, Occupational Diseases, Federal Institute for Occupational Safety and Health, Nööldnerstr. 40-42, Berlin, 10317 Germany

**Keywords:** Physical inactivity, Sitting behaviour, Sitting breaks, Teleworking, Hybrid working, Health promotion

## Abstract

**Background:**

Sedentary behaviour (SB) and the lack of physical activity (PA) are associated with negative health outcomes. Among desk-based workers, sitting at work contributes substantially to the daily time spent sedentary. Working environment can influence SB. Thus, we aimed to investigate the evidence on the impact of working from home/teleworking (WFH), which is now a common working environment versus working onsite on SB and PA.

**Methods:**

We conducted a systematic review comparing SB and PA of workers WFH compared to onsite work. We searched Pubmed, Embase and SPORTDiscus (last search: June 2025). At least two reviewers independently screened the studies and rated the of risk of bias based on adapted existing tools. We included studies on adult workers, which at least part-time WFH with comparison group working onsite, reporting SB or/and PA-outcomes per workday/work time. Data extraction was done by one reviewer and checked by two reviewers. Results were described qualitatively and random-effect meta-analyses for daily sedentary time (ST), sitting breaks, and steps were performed.

**Results:**

We included 38 studies (from 42 articles, with *n* = 282,264 subjects) comparing WFH and onsite work. Four of these studies were rated as having a “low” risk of bias. SB was described in 23 studies (with *n* = 209,267 subjects). A meta-analysis of studies reporting quantitative results suggests an increase in ST of 31 min (95% CI 14 to 48; I^2^ = 57.5%; 7 studies) during work hours when WFH. PA was described in 36 studies (with *n* = 270,617 subjects), and the meta-analysis found a decrease in daily steps of 2564 (mean difference: − 2564; 95% CI -3809 to -1320, 289; I^2^ = 91.4%; 7 studies) when WFH.

**Conclusion:**

We found SB tends to increase and daily steps tend to decrease when WFH compared to onsite work. Studies of PA varied in their methods and results, and few studies measured movement. As most of the studies (*n* = 31) were conducted during the COVID-19 pandemic, that may have influenced the results. Nevertheless, workplace interventions that aim to reduce SB and promote PA need to be adapted to the home working environment.

**Registration number:**

CRD42022349442.

**Supplementary Information:**

The online version contains supplementary material available at 10.1186/s12889-025-24960-x.

## Background

During the COVID-19 pandemic, working from home/teleworking (WFH) was used to prevent the transmission of the virus in the population. After the end of the pandemic situation, many workers in European countries continue to WFH or work hybrid, which means WFH and work onsite (2024: “usually” or “sometimes” 22.2% of all workers) [1].This change in working environment may also impact health by changing how employees sit and move during working hours and throughout a workday.

Long uninterrupted sitting time (ST) and the lack of physical activity (PA) are associated with diverse health risks, such as cardiovascular disease and type 2 diabetes [2–5]. Sedentary behaviour (SB) is defined as any waking behaviour with an energy expenditure less than 1.5 metabolic equivalents of tasks (METs) while sitting, reclining, or lying down [6]. When examining SB and its influence on health, also sitting patterns are important [7, 8], which are often described using sitting bouts or sitting breaks (e.g., [9–11]). In contrast, PA is defined as any bodily movement produced by skeletal muscles that requires an energy expenditure. PA can be categorized according to energy consumption into vigorous PA (≥ 6 METs), moderate PA (>3–6 METs), and light PA (>1.5–3.5 METs). The World Health Organization (WHO) Guideline recommends at least 150 min per week of moderate PA. Physical inactivity (PI) is the failure to achieve global recommendations for PA [5]. 

PI and SB are both risk factors for health problems, and can reinforce each other. While PA can counteract the effect of long periods of sitting [12, 13], when daily ST exceeds 8 h, the recommended 150 min of moderate PA is not enough to counteract the health risks of sitting [5, 13].

More than one quarter of adults do not adhere to the recommendations for PA [14] and high levels of ST are common in the working population [15]. Furthermore, the odds for prolonged SB increased in persons aged 21–65 years over the last years [16].

Workers able to WFH are typically desk workers in digitalized fields. The use of digital tools, often used for work, is positively associated with ST [17]. Prolonged sitting at work contributes to total daily SB [15] that can increase the risk for disease [12]. While movement is naturally integrated into an onsite workday (e.g., the commute to work, longer distances to central places), many opportunities for movement during working hours are greatly reduced when WFH [18, 19].

A review from Wilms et al. [20] investigated the impact of WFH in combination with COVID-19 restrictions on health behaviours like PA/SB as percentages of change, but did not try to explicitly examine the effect of WFH alone. Also, a meta-analysis by Chaudhary et al. [21] focussed on the abrupt change in work practice (transition to WFH) due to the pandemic, and a meta-analysis by Polspoel et al. [22] analysed the effect of WFH on time spent with different levels of PA and ST. Chaudhary et al. [21] and Polspoel et al. [22] both report the pooled effect magnitudes as Hedge’s g. While Hedge’s g provides an indication of the overall effect by making results on different measurement scales comparable, the standardised scale is an abstract representation of effect magnitude. Sers et al. [23] also conducted a systematic literature search for evidence focusing on the relationship between WFH and PA, SB and sleep of healthy working adults and summarized the results visually with an effect direction plot. None of the reviews [20–23] analysed sitting breaks/sitting bouts or differentiated between worktime and total time awake.

### Aims

Thus, we conducted a systematic review of the literature to examine the impact of WFH compared to onsite work on SB, in terms of ST and variables describing sitting patterns (e.g., sitting breaks) and PA of workers. According the population, intervention/exposure, comparison, and outcome (PICO) framework, we addressed the following research questions among people in paid employment:

1. What impact does work from home/teleworking have on sedentary behaviour compared to onsite working during work or a workday?
2. What impact does work from home/teleworking have on physical activity compared to onsite working during work or a workday?
3. Is the proportion of time spent working from home/teleworking associated with the total time spent sitting/sedentary or the amount of physical activity during work or on a workday (dose-response relationship)?

We also planned to determine if results systematically differ according to the method used to measure SB or PA (accelerometer vs. questionnaire) and if the literature indicates if there are groups (i.e., occupation, gender, ages) at particular risk for increased SB when WFH.

## Methods

### Literature search

The procedures for the systematic review were published a priori in the PROSPERO database under the number CRD42022349442 and we reported according to “Preferred Reporting Items for Systematic reviews and Meta-Analyses” (PRISMA) [24]. We conducted a systematic search of the literature in the MEDLINE, Embase, and SPORTDiscus databases on 31 August 2022 (MEDLINE and Embase) and 5 September 2022 (SPORTDiscus). We updated the search to include publications indexed up to 26 June 2025 (MEDLINE, Embase, SportDiscus). Databases were selected based on the experience of the “Sitting@work” project [25]. Search string was developed by two researchers (CS, EB) and cross-checked by a third researcher (JH).

The following PubMed search string was used for each database:

(seated OR sedentar* OR sit OR sitt* OR “physical activity” OR “physical activit*” OR “physical exercise” OR “physical fitness”) AND (“flexible work*” OR “flexibility work*” OR “home-based work*” OR homeoffice OR “home office” OR “home work” OR “home working” OR homeworking OR “mobile work*” OR “remote work*” OR telecommuting OR telework* OR “virtual work” OR “virtual office” OR “work at home” OR “working at home” OR “work from home” OR “working from home”).

Medical Subject Headings (MeSH) were not included as they did not have an impact on the number of hits. References of included studies were searched for additional relevant articles (backward search). No forward search was performed. Endnote was used to document the search results. These were subsequently imported into the software tool Rayyan for identification and removal of duplicates and screening documentation [26].

### Screening

The screening of titles and abstracts was conducted independently by at least two researchers (CS, EB, MS, JH). In case of discrepancies, consensus was sought with a third researcher (JH, CB, CS). The screening of the full texts was done in a similar fashion by the same researchers (CS, EB, MS, JH, CB) after a pilot screening of a subset of the publications conducted by three researchers.

### Inclusion and exclusion criteria

We included studies investigating populations of workers or employees in paid employment that compared SB and PA, when working at least partially from home or in telework versus onsite working. Studies should have assessed WFH among individual study participants. A quantification of WFH in terms of workdays, hours, or percentage of work time was advantageous for examining the effects of response but not necessary for inclusion. Sitting behaviour and PA could be recorded using accelerometric measurements or with questionnaires.

Studies of children and animals, studies without reference to WFH, or studies only reporting aggregated data on WFH (e.g., assuming an increase in WFH in a population due to lockdown measures), and studies without possibility for comparison of WFH and onsite work were excluded.

We included longitudinal cohorts, cross-sectional studies, and intervention studies, because these study designs can provide quantitative results for individual participants. Qualitative studies, ecological studies, editorials, and reviews were excluded. Grey literature could be included but was not explicitly sought. Studies not published in English or German were excluded.

### Data extraction

Study characteristics and results were extracted by one researcher (CS, EB or MS) and checked for accuracy and completeness by at least a second researcher (CS, EB, MS or JH). The extraction of study characteristics included information on author, publication, study location, study design, population characteristics and occupational settings, definition and measurement of exposure, relevant outcomes, and outcome measurement. The extraction of study results included any results (descriptive and analytic) that provided information on differences in SB and PA while WFH compared to onsite work. As we included also studies with not only desk-based workers, we used the terminology “usual workplace” (usual WP) when work was not performed at home. If studies included only desk workers, we used the terminology “working at the office” (WAO). When communicating mixed results and in the section discussion, we used the term “onsite work” as comparison to WFH. This included both descriptions of activities during working hours and during the entire day on workdays. If a study was described in more than one publication, the data of the publications were extracted separately. We selected the publication providing results best suited (e.g., due to operationalisation of exposure, quality of statistical analysis) to answer our research questions and included that one in the qualitative synthesis, and when possible, in the meta-analysis.

### Risk of bias

Risk of bias was assessed with a tool based on the structure (major and minor domains) and content of the risk of bias instrument described in Romero Starke et al. [27] and Bolm-Audorff et al. [28]. We formulated our criteria also according to existing checklists of the Joanna Briggs Institute [29], the National Institute of Health [30], and the Appraisal tool for Cross-Sectional Studies (AXIS) [31].

Domains considered to be of major relevance for the internal validity of the study results were the study population, recruitment procedures, exposure assessment, adequacy of comparison group, outcome assessment, recall bias, confounding, and statistical analysis. Minor domains included ethics (ethical board review, informed consent) and conflicts of interest (e.g., due to funding sources). We also created a new minor domain we called “pandemic bias”. Many of the studies were conducted during the pandemic, when a number of infection prevention measures to reduce COVID-19 transmissions (e.g., stay-at-home orders, voluntary isolation measures, fitness studio closures) reduced the generalizability of the results to post-pandemic conditions. Criteria for “low” pandemic bias were: data collection before the start of the pandemic. Furthermore, “low pandemic” bias was selected if authors indicated that data collection started after the end of the pandemic. Because this did not reduce the internal validity of the studies, we considered “pandemic bias” as an additional minor domain of potential bias relevant to the external validity of the results. The risk of bias assessment form used, with examples for rating single domains, is included in the online additional files (Additional file 1). The risk of bias was assessed independently by at least two researchers (CS, EB, JH, MS) and discussed at length until consensus was reached (CS, EB, MS, CB, JH).

To determine the overall risk of bias of each study, we considered the major domains of the risk of bias assessment form (minor domains did not impact the overall judgement). All studies with no “high” risk of bias in all the major domains were rated with an overall “low” risk of bias (+). Studies with only one major domain with “high” risk of bias were classified overall into “almost low” risk of bias (+/-), and studies with more than one major domain with “high” risk of bias were rated overall as “high” risk of bias (-).

### Synthesis of results

#### Qualitative synthesis

For the qualitative analysis, SB and PA were considered separately. We narratively summarised which studies indicated an increase, a decrease, or no change of sitting behaviour or PA when WFH. Studies investigating only desk/office workers, studies that measured SB and PA and studies without pandemic bias were considered separately. Results addressing dose-response relationships between the amount of WFH and the outcomes were also summarised separately. Results are presented as tables structured according to their risk of bias and outcome parameter including author, main findings and statistical approach.

#### Meta-analysis

If similar outcome parameters were reported in the included studies, quantitative data were included in the meta-analyses. Meta-analyses considered differences in mean ST in minutes (minutes/day or minutes/work), odds ratios (OR) of increased SB (≥ 8 h sitting or + ≥ 2 h increase in sitting), differences in the mean number of steps (steps/day or steps/work time), and differences of mean number of sitting breaks (sitting breaks/day or sitting breaks/work) when WFH vs. onsite work. Meta-analysis was performed in STATA (Version 18) using a random effects model with the estimation method of DerSimonian and Laird [32]. Analysis was also performed by second researcher in SPSS (Version 29) to ensure quality [33]. If only 95% confidence interval (CI) of OR were given, log standard errors (SE) were calculated by formula [1] with upper CI (UCI) and lower CI (LCI).

1 $$\:\mathrm{ln}\left(SE\right)=\:\frac{\mathrm{ln}\left(UCI\right)-\mathrm{l}\mathrm{n}\left(LCI\right)}{3.92}$$

If only a standard deviation (SD) and number of participants (n) was given, SE were calculated using formula [2]. Results are presented as forest plots. If further data of an included study were needed, corresponding authors were contacted and asked to provide information. Funnel plots were not created due to low number of studies included in the meta-analysis.

2 $$\:\mathrm{S}\mathrm{E}=\frac{\mathrm{S}\mathrm{D}}{\sqrt{n}}$$

## Results

### Study selection and risk of bias

**Fig. 1:**
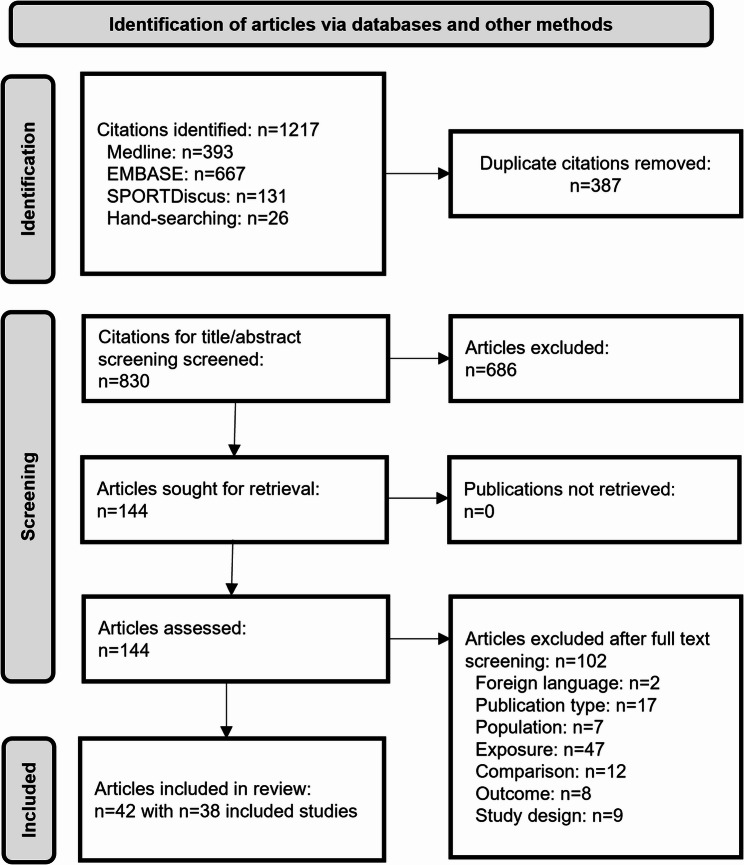
PRISMA Diagram depicting the literature search and selection of studies (and publications)

We found *n* = 1217 citations in databases, of which we removed *n* = 387 as duplicate. After title/abstract screening we excluded *n* = 686 articles. This resulted in *n* = 144 articles for the full-text-screening of whom 102 were excluded. We included 38 studies (*n* = 282,264 subjects), described in 42 articles (Fig. 1). Three studies were each described in two or more published articles. This was the case for the “Reducing Sedentary Behaviour on Blood Pressure (RESET BP)” study [10, 34, 35], the “Lifelines” study [36, 37] and the “COVID Inconfidentes” study [38, 39]. For the RESET BP study and the “Lifelines” study, we focused on the publications providing results most applicable to our research questions [10, 36] for the synthesis of results. Specifically, we used Holmes et al. [10] for the RESET BP study due to better statistical analysis and Loef et al. [36] for the “Lifelines” study as it provided more applicable results to our research question. For the “COVID Inconfidentes” study we used results of Moura et al. [38] for research questions 1 and 2 and the results of Moura et al. [39] for research question 3 due to the operationalisation of the exposure (WFH). Nonetheless, each of the publications were extracted, but considered to be one study (Additional file 2).

SB was investigated by 23 studies with *n* = 209,267 subjects, and 36 studies with *n* = 270,617 subjects investigated PA. The studies were conducted in 15 countries (Japan *n* = 9, USA *n* = 7, Brazil *n* = 3, Germany *n* = 4, Sweden *n* = 3, Australia *n* = 2, Netherlands *n* = 2, France *n* = 1, India = 1, Qatar *n* = 1, Singapore *n* = 1, Italy = 1, Belgium = 1, UK = 1, Finland = 1). Of the 38 included studies, 31 were cross-sectional (including also surveys and field studies) and seven studies had a longitudinal study design (e.g., cohort, panel) (Table 1). Secondary outcomes like sleep, weight, depression, or nutritional habits were described in 16 of the 38 articles (Additional file 2, Table A1). A table of excluded studies with reason for exclusion is given in additional file 3.

**Table 1:** Study characteristics of included studies

Study	Country	Design	Population	Exposure vs. Comparison	Outcome(s)	Notes
Abed Alah et al. (2022) [40]	Qatar	CS	All workers	WFH vs. usual WP	ΔPA, ΔSB (h/day)	-
Bérard et al. (2021) [41]	France	CS	All workers	WFH vs. usual WP	ΔPA (min/week)	-
Cobbold et al. (2024) [42]	Australia	CS	All workers	Less or same WFH vs. more WFH than pre-pandemic	ΔPA (min/week)	-
De Oliveira da Silva Scaranni et al. (2023) [43]	Brazil	CS^1^	Civil servants	WFH vs. usual WP	SB (> 8 h/day), PI (WHO recommendation) & domestic PA	-
Delanoeije et al. (2024) [44]	Belgium	CS	Office workers	WFH vs. usual WP	PA (PA-score)	-
Elangovan et al. (2021) [45]	India	CS^2^	Government & private sector workers	WFH vs. WAO	ΔPA (exercise/week since lockdown)	-
Fukushima et al. (2021) [46]	Japan	CS^3^	All workers	WFH vs. usual WP	Occupational PA/SB, standing, walking, sitting, heavy PA (min), SB bout length (min)	-
Grubben et al. (2022) [47]	Netherlands	CS^1^	All workers	WFH (≥ 1 h/week) vs. usual WP (< 1 h/week WFH), h/week WFH	PA (sports participation in the last 3-months)	-
Hallman et al. (2021) [48]	Sweden	CS^4^	Office workers	WFH days vs. WAO days	PA at work: SB, standing, moving time	Axivity AX3
Henke et al. (2016) [49]	USA	LS	Office workers	WFH vs. WAO	Prevalence PI (cardio-exercise < 3days/week)	Not during pandemic
Herbolsheimer et al. (2024) [50]	Germany	CS	General population	WFH vs. usual WP	SB & PA (change of PA in different domains compared to pre-Covid in categories and adherence to WHO guidelines)	-
Holmes et al. (2023) [10]; Barone Gibbs et al. (2021) [34]; Holmes et al. (2025) [35]	USA	CS^1^	Office workers	WFH vs. WAO	PA & SB (min/workday), sitting bouts at work (≥ 30; ≥60 min), STS (no/day)	ActivPALmicro
Ishibashi et al. (2022) [18]	Japan	CS^3^	Secondary/tertiary sectors	WFH (always, pandemic only) vs. usual WP	PA, MET	-
Kikuchi et al. (2025) [51]	Japan	CS^4^	Office workers	WFH vs. WAO	PA (steps, min/day) & SB (min/day)	Active style Pro HJA-750 C
Kim et al. (2022) [52]	Japan	CS^1^	Office workers	WFH vs. WAO	PA (min/day & steps/day), SB (min/day),	Active style Pro HJA-350IT
Kitano et al. (2024) [53]	Japan	CS	Office workers	WFH vs. WAO	SB & PA (min/day), prolonged SB (times/day and min/time)	Pro HJA750-C
Javad Koohsari et al. (2021) [54]	Japan	LS	All workers	Absolute change of days WFH	ΔPA & ΔSB (h/day)	-
Koyama et al. (2021) [55]	Japan	CS	All workers	WFH vs. usual WP	SB (+ ≥ 2 h/day after state of emergency)	-
Leskinen et al. (2025) [56]	Finland	CS	All workers	WFH vs. usual WP	SB & PA in min/day	ActiGraph wActiSleep BT
Loef et al. (2022a; 2022b) [36] [37]	Netherlands	LS	All workers	WFH/Hybrid vs. usual WP	PA (moderate to vigorous PA ≥ 150 min/week), SB sitting ≥ 8 h/day)	-
Marenus et al. (2025) [57]	USA	CS	All workers	WFH vs. hybrid vs. usual WP	PA (MET-min/week)	-
Massar et al. (2023) [58]	Singapore	LS^1^	Working adults	WFH vs. usual WP	PA (daily steps)	Fitbit Versa 2
Matthews et al. (2022) [59]	USA	LS	General population	WFH (always/some) vs. usual WP	PA & SB (h/day)	-
Moura et al. (2022; 2023) [38] [39]	Brazil	CS	General population	WFH (some/always) vs. usual WP	PA during leisure time (yes/no)	-
Olsen et al. (2018) [60]	Australia	LS	Office workers	WFH vs. WAO	SB (min/workday)	Not during pandemic
Oxenham et al. (2025) [61]	UK	LS	All workers	WFH vs. usual WP	PA (MET-min/week)	-
Prince et al. (2024) [62]	Canada	CS	All workers	WFH vs. usual WP	PA (moderate to vigorous PA ≥ 150 min/week, min/week)	-
Sauter et al. (2025) [63]	Germany	CS	Office workers	WFH vs. WAO	PA (steps/day & steps/work time) & SB (min/day & min/work time), STS (counts/work & counts/day)	ActivPAL3
Scurati et al. (2025) [64]	Italy	CS	Office workers	WFH vs. WAO	PA (MET-min/week & min/week) & SB (min/day or min/week)	Axivity AX3
Sers et al. (2023) [9]	Germany	CS^4^	Office workers	WFH vs. WAO	PA & SB (h/day), SB breaks (no/day), SB bouts (no/day)	Move4
Silva et al. (2021) [65]	Brazil	CS	All workers	WFH vs. usual WP	PI (≤ 150 min/week)	-
Suzuki et al. (2025) [66]	Japan	CS	All workers	WFH vs. usual WP	Change of PA during the COVID-19 pandemic	-
Thralls Butte et al. (2023) [67]	USA	CS	Office workers	WFH vs. WAO vs. Hybrid	PA (min/day & steps/day), SB (min/day), STS (no/day)	ActivPAL
Tomonaga et al. (2024) [11]	Japan	CS	White collar workers	WFH vs. WAO	PA (kcal, min), SB (min), SB bouts & sedentary break (count)	Not during pandemic, Active style Pro HJA-750 C
Wahlström et al. (2023) [68]	Sweden	CS	Office workers	WFH vs. WAO	PA (time in min standing, walking, stair walking, running), SB (time in min sitting, time in short, moderate and long sitting bouts)	Axivity AX3
Wallmann-Sperlich et al. (2023) [69]	Germany	CS	All workers (WFH >0%)	Different proportions of WFH (1–25%, 26–50%, 51–75%, 76–99% & 100% WFH)	PA (%walking of working hours) & SB (%sitting of working hours), sitting breaks (no/hour)	-
Webber et al. (2023) [70]	USA	CS	All workers	Never WFH vs. more WFH vs. same WFH vs. less WFH	Change of PA compared to pre-pandemic and PA level (≥ 150 min moderate to vigorous VPA)	-
Widar et al. (2021) [71]	Sweden	CS^4^	University staff	WFH vs. WAO	PA (walking, running, cycling in min/day), stand (min/day), SB (sit/lie min/day), STS (no)	Not during pandemic, AX3 accelerometer

*Abbreviations*: *CS* Cross sectional, survey or field studies, *h* hours, kcal – kilocalorie, *LS* Longitudinal (cohort/panel) study, *MET* Metabolic equivalent, *min* Minutes, *no* Number, *PA* Physical activity, *PI* Physical inactivity, *SB* Sedentary behaviour, *STS* Sit to stand transitions, *WAO* Work at the office, *WFH* Work from home, *WP* Workplace

^1^embedded in longitudinal or intervention study ^2^convenience sample ^3^repeated cross-sectional ^4^observational field study

In relation to the risk of bias assessment, four of the 38 studies were rated with a “low risk of bias” (+), ten were considered “almost low risk of bias” (+/-), and 24 studies had a “high risk of bias” (-) (Table 2). Seven studies were not biased by COVID-19 pandemic conditions.

**Table 2:** Risk of bias assessment of major and minor domains

Study	Major Domains								Minor Domains			Overall
	Pop-ulation	Selection process	Exposure	Compari-son	Outcome	Recall Bias	Con-founding	Statistical Analysis	Pandemic Bias	Funding	Ethics	
Abed Alah et al. (2022) [40]	+	-	+	-	+	-	-	-	-	+	+	-
Barone Gibbs et al. (2021) [34]	+	+	+	+	+	+	-	-	-	+	+	-
Bérard et al. (2021) [41]	+	+	-	-	-	-	-	+	-	+	+	-
Cobbold et al. (2024) [42]	+	-	-	-	+	-	-	-	-	+	+	-
De Oliveira da Silva Scaranni et al. (2023) [43]	+	+	+	+	+	+	+	+	-	+	+	+
Delanoeije et al. (2024) [44]	-	-	+	+	+	-	+	+	-	+	+	-
Elangovan et al. (2021) [45]	+	-	-	-	-	+	-	-	-	-	+	-
Fukushima et al. (2021) [46]	+	+	+	-	+	+	+	+	-	+	+	+/-
Grubben et al. (2022) [47]	+	+	+	-	+	-	-	-	-	+	+	-
Hallman et al. (2021) [48]	+	+	+	+	+	+	+	+	-	+	+	+
Henke et al. (2016) [49]	+	+	+	+	-	+	+	+	+	-	-	+/-
Herbolsheimer et al. (2024) [50]	+	+	+	-	+/-^1^	-	-	+	-	+	+	-
Holmes et al. (2023) [10]	+	+	+	+	+	+	+	+	-	+	+	+
Holmes et al. (2025) [35]	+	+	+	+	+	+	+	+	-	+	+	+
Ishibashi et al. (2022) [18]	+	-	+	-	+	+	-	-	-	+	+	-
Kikuchi et al. (2025) [51]	+	-	+	+	+	+	+	+	+	+	+	+/-
Kim et al. (2022) [52]	+	-	-	+	+	+	+	-	-	+	+	-
Kitano et al. (2024) [53]	+	-	+	+	+	+	+	+	+	+	+	+/-
Javad Koohsari et al. (2021) [54]	+	-	+	-	+	-	-	+	-	+	+	-
Koyama et al. (2021) [55]	+	+	+	-	-	-	+	+	-	+	+	-
Leskinen et al. (2025) [56]	+	-	+	+	+	+	+	+	-	+	+	+/-
Loef et al. (2022a, b) [36] [37]	+	+	+	-	+	+	+	+	-	+	+	+/-
Marenus et al. (2025) [57]	-	-	-	-	+	+	-	-	-	+	+	-
Massar et al. (2023) [58]	+	-	+	-	+	+	-	+	-	+	+	-
Matthews et al. (2022) [59]	+	-	+	-	+	+	-	-	-	+	+	-
Moura et al. (2022) [38]	+	+	+	-	+	+/-^2^	-	+	-	+	+	-
Moura et al. (2023) [39]	+	+	+	-	+	+	-	+	-	+	+	-
Olsen et al. (2018) [60]	+	-	+	+	+	+	+	-	+	+	+	-
Oxenham et al. (2025) [61]	+	+	-	-	+	+	-	-	-	+	+	-
Prince et al. (2024) [62]	+	+	+	-	+	+	-	+	-	+	+	-
Sauter et al. (2025) [63]	+	-	+	+	+	+	+	+	-	+	+	+/-
Scurati et al. (2025) [64]	+	-	+	-	+	+	-	-	+	+	+	-
Sers et al. (2023) [9]	+	-	+	+	+	+	+	+	-	+	+	+/-
Silva et al. (2021) [65]	-	-	-	-	+	+	-	-	-	+	+	-
Suzuki et al. (2025) [66]	+	-	+	-	-	-	-	-	-	+	+	-
Thralls Butte et al. (2023) [67]	+	-	+	+	+	+	-	+	-	+	+	-
Tomonaga et al. (2024) [11]	+	+	+	+	+	+	+	+	+	+	+	+
Wahlström et al. (2023) [68]	+	-	+	+	+	+	+	+	-	+	+	+/-
Wallmann-Sperlich et al. (2023) [69]	+	+	+	-	+	+	-	-	-	+	+	-
Webber et al. (2023) [70]	+	+	-	-	+/-^1^	-	-	+	-	+	+	-
Widar et al. (2021) [71]	+	-	+	+	+	+	+	+	+	+	+	+/-

*Abbreviations*: low risk of bias: +, almost low risk of bias: +/-, high risk of bias: -

^1^one outcome variable was rated with low risk of bias and one with high; ^2^one outcome variable was rated with low risk for recall bias and one with high

### Sedentary behaviour

In total, 23 studies investigated SB. Of these, four studies had a “low”, nine studies an “almost low”, and ten studies had a “high” risk of bias.

#### Sedentary time

Overall, 16 studies reported an increase in ST when WFH compared to onsite work [10, 11, 36, 40, 43, 46, 50, 51, 53–56, 59, 63, 67, 69]. Six studies showed no change [9, 48, 52, 64, 68, 71], and one study found ST decreased when WFH compared to WAO [60]. Among the studies with a “low” risk of bias, three out of four studies report an increased ST compared to onsite work [10, 11, 43], while one study reported no markedly change [48]. In the “almost low” risk of bias category six out of nine studies reported increased ST compared to onsite work [36, 46, 51, 53, 56, 63], while three studies found no change [9, 68, 71]. Among the studies with a “high” risk of bias, seven out of ten studies reported an increase in ST when WFH compared to onsite work [40, 50, 54, 55, 59, 67, 69], while two observed no change [52, 64] and one decreased ST when WFH compared to WAO [60] (Table 3).

**Table 3:** Main results sedentary behaviour (*n* = 23)

Study	Main Results – Sedentary Behaviour (SB)				
Studies with low risk of bias (*n*=4)					
De Oliveira da Silva Scaranni et al. (2023) [43]	SB (>8h/day) increased when WFH compared to usual WP Logistic regression, adjusted sitting time >8h/day: adjusted OR 2.68 (95% CI 2.02, 3.56)				
Hallman et al. (2021) [48]	No markedly difference in the distribution of SB, standing and moving during work or leisure time (sitting time min/day) between WFH vs. WAO				
	Descriptive analysis, unadjusted				
				WAO days mean (SD)	WFH days mean (SD)
	Total time at work in min/day			512 (165)	486 (205)
	Sedentary in min/day			373 (86)	361 (116)
	Standing in min/day			102 (63)	88 (63)
	Total leisure time in min/day			468 (128)	461 (159)
	Sedentary in min/day			258 (50)	256 (71)
	Standing in min/day			141 (44)	143 (58)
	Statistical results of univariate models of compositional data (n= 27) show that behaviours during work and leisure did not change markedly on days WFH compared with days not WFH.				
Holmes et al. (2023)^1^[10]	SB (sitting time/workday, sitting time of 30- and 60-min bouts/workday) increased when WFH and sit-to-stand transitions decreased during workday when WFH				
	Linear regression, adjusted				
				WAO adjusted β± SE	WFH
	SB (min/workday)			−17.2±8.4**	Ref.
	SB30 (min/workday)			−39.1±12.8**	Ref.
	SB60 (min/workday)			−41.3±11.8**	Ref.
	Sit-to-Stand Transitions (no/workday)			+2.1±1.3	Ref.
	Standing time (min/workday)			+13.0±7.1*	Ref.
	*0.05≤p≤0.10 **p≤0.05				
Tomonaga et al. (2024) [11]	Sitting time, and sedentary bouts (>30 min; >60 min) increased and number of sedentary breaks decreased when WFH				
	Multivariate analysis of variance, adjusted				
	Comparison between WFH and WAO including commute				
	Parameter		WAO mean^1^ (95% CI)	WFH mean^1^ (95% CI)	*p*-value
	Sitting time in min		501.7 (485.8, 517.6)	571.6 (555.7, 587.5)	<0.01
	Sedentary break, count		44.2 (40.2, 48.2)	37.4 (33.3, 41.4)	<0.05
	Sedentary bout >30 min, count		4.8 (4.3, 5.2)	5.6 (5.1, 6.1)	<0.01
	Sedentary bout >60 min, count		1.4 (1.0, 1.7)	2.0 (1.7, 2.4)	<0.05
	^1^indicated as least squares mean				
Studies with almost low risk of bias (*n*=9)					
Fukushima et al. (2021) [46]	SB (min/work time) increased and SB bout length increased when WFH compared to usual WP				
	Analysis of covariance, adjusted values				
	Mean time spent in each behaviour during working time (min)				
			Usual WP mean (SE)	WFH mean (SE)	*p*-value
	SB in min		179.7 (17.4)	256.2 (18.7)	<0.001
	SB bout length in min		24.0 (3.2)	33.6 (3.5)	<0.001
Kikuchi et al. (2025) [51]	SB (min/day) increased when WFH compared to WAO				
	Descriptive results, intra-individual comparison				
	Daily time of sedentary behaviour by work location				
				WFH	WAO
	SB in min/day (SD)			715.5 (147.3)	694.9 (121.9)
Kitano et al. (2024) [53]	SB (min/day, prolonged SB min/day, prolonged SB counts/day, prolonged SB bout duration) increased with increased WFH frequency				
	Multiple linear regression, adjusted				
	Estimated marginal means with 95% CI (based on personal communication)				
		Never WFH	1–2 days WFH	3–4 days WFH	≥5 days WFH
	Time in SB (min/day)	584.5 (574.3–594.8)	625.0 (611.4–638.6)	644.6 (630.4–658.8)	657.0 (644.6–669.5)
	Prolonged SB (min/day)	258.9 (2410.8-277.0)	315.1 (291.2–339.1)	357.9 (332.6–383.2)	374.2 (352.2–369.1)
	Prolonged SB counts (times/day)	4.5 (4.2–4.7)	5.4 (5.1–60.7)	6.0 (5.6–6.3)	6.1 (5.8–6.4)
	Prolonged SB bout duration	56.1 851.4-60.7)	56.8 (50.5–63.1)	59.3 (52.8–65.8)	63.6 (58.0–69.3)
Leskinen et al. (2025) [56]	Higher SB (min/work time and min/day) when WFH compared to WAO				
	Generalised linear models and linear mixed models, adjusted				
	Daily SB for workdays by work mode groups of non-manual workers				
			Usual WP	Hybrid	WFH
	Time in SB in min/day (95% CI)		584 (521–646)	650 (588–712)*	666 (596–712)*
	Time in occupational SB in min/work time (95% CI)		288 (232–343)	343 (287–399)*	337 (273–401)*
	*Significant difference compared to in-office workers.				
	Daily SB for days WFH and WAO among hybrid workers				
				Days WFH	Days WAO
	Time in SB (min/day)			624 (589–658)	594 (560–629)
	Time in occupational SB (min/work time)			341 (319–362)	315 (293–337)
Loef et al. (2022a)^2 ^[36]	SB (sitting ≥8 h/day) increased when WFH				
	Logistic generalised estimating equations analysis, adjusted values				
	SB on workdays during pandemic				
				OR (95% CI)	
	WFH			1.94 (1.83, 2.06)	
	Hybrid workers			1.73 (1.59, 1.88)	
	Usual WP			1 (Ref.)	
Sauter et al. (2025) [63]	SB (min/work time and min/day) increased when WFH compared to WAO, STS (counts/work and counts/day) increased when WFH compared to WAO				
	Linear mixed models, adjusted				
	Estimated marginal means (based on own data)				
				Days WFH (95% CI)	Days WAO (95% CI)
	SB in min/work time			360 (323–396)	314 (278–350)
	STS in counts/work			28.9 (24.7–33.2)	25.4 (21.2–29.6)
	SB in min/day			637 (585–688)	605 (554–657)
	STS in counts/day			57.9 (50.9–65.0)	50.9 (43.8–58.0)
Sers et al. (2023) [9]	SB (SB time, number of SB breaks and short to long SB bouts) is not associated with the working environment				
	Multilevel model analysis, adjusted and descriptive statistics by work environment (WAO vs. WFH)				
				Days WAO mean (SD)	mean (SD)
	SB time (h/day)			9.61 (2.53)	9.92 (2.55)
	Models of SB-related outcomes			WFH	WAO
				b (SE)	
	SB time (h/day)			0.16 (0.37)	Ref.
	SB breaks (no/day)			1.78 (1.84)	Ref.
	Short SB bouts (no/day)			1.95 (1.09)	Ref.
	Short to moderate SB bouts (no/day)			0.05 (0.59)	Ref.
	Moderate-to-long SB bouts (no/day)			−0.36 (0.35)	Ref.
	Long SB bouts (no/day)			0.36 (0.26)	Ref.
	Note: all *p*-values ≥0.05				
Wahlström et al. (2023) [68]	SB does not differ clearly between WFH and WAO				
	Descriptive analysis and multilevel linear mixed models, post-hoc pairwise comparison between WAO and WFH, adjusted				
				WAO days mean (SD)	WFH days mean (SD)
	Sedentary time in h^a^			10.6 (1.9)	10.6 (1.8)
	No statistically significant (*p*<0.05) difference between WAO days and WFH days was detected.				
	^a^personal communication				
Widar et al. (2021) [71]	SB: no difference for time sitting (min/day) in WFH and WAO; more sit-to-stand transitions during WFH hours than during WAO hours				
	Descriptive statistics by working environment (unadjusted) and analysis of variance (adjusted)				
				During work mean^1^	Total time mean^1^
	Sit/lie (min/day)		WFH	324	551
			WAO	312	575
	Stand (min/day)		WFH	78	155
			WAO	73	157
	^1^geometrical mean, SE not reported				
	There was a significant workplace and time interaction effect for the number of sit-to-stand-transitions (ղ2=0.194; p=0.021), with more transitions being made during WFH hours than during WAO hours.				
Studies with high risk of bias (*n*=10)					
Abed Alah et al. (2022) [40]	DSB h/day increased in WFH vs. usual WP				
	Mann-Whitney U-Test, unadjusted				
	Sitting/reclining time difference (h/day) mean ranks: WFH: 586.5; usual WP: 474.7 (*P*<0.001)				
Herbolsheimer et al. (2024) [50]	Change to WFH increased sedentary time				
	Multivariable linear regression models, adjusted				
	WFH was related to increased sedentary time (β=0.21; 95% CI: 0.203, 0.216)^a^				
	^a^personal communication				
Kim et al. (2022) [52]	SB (sitting time min/day) – no markedly difference				
	Descriptive analysis, unadjusted				
				WAO mean (SD)	WFH mean (SD)
	Sitting, min/day			627.8 (98.8)	610.0 (104.6)
Javad Koohsari et al. (2021) [54]	Work-related DST and total DST (h/day) and absolute change of days WFH (before and after first COVID-19 outbreak) are positively associated				
	Multivariable linear regression models, adjusted Associations between absolute changes in WFH days and changes in workers’ domain-				
	specific SB (complete data of 1086 individuals)				
				Work-related Sitting-Time (h/day)	Total Sitting Time (h/day)
				b (95% CI)	b (95% CI)
	Working from home (days/week)			0.16 (0.08, 0.24)*	0.23 (0.11, 0.36)*
	*p<0.05.				
Koyama et al. (2021) [55]	SB: higher chance of increased sitting (≥2h/day) if starting WFH				
	Logistic regression, adjusted				
	Adjusted OR (95% CI) of prolonged sedentary time (≥2hours) according to job category and if WFH during the state of emergency.				
				Usual WP OR (95% CI)	WFH OR (95% CI)
	Blue-collar worker			1.00 Ref.	2.02 (1.05, 3.91)
	Desk worker			1.61 (1.30, 2.00)	3.05 (2.33, 4.01)
	Salesperson			1.91 (1.52, 2.41)	5.15 (3.75, 7.07)
	Desk workers who started WFH had an adjusted OR 1.89 (95% CI 1.34–2.68) *[self-calculated]* for sitting ≥2h/day longer during the state of emergency compared to desk workers who did not.				
Matthews et al. (2022) [59]	SB (sedentary time h/day) increased when WFH				
	Descriptive analysis, unadjusted				
	Pre-pandemic means from 2019, unadjusted				
				WFH mean	Usual WP mean
	Sedentary time (h/day)			10.96	9.13
	SE not reported				
Olsen et al. (2018) [60]	SB (Sitting on a usual workday (min/day) is reduced by one hour when WFH				
	Descriptive results, unadjusted				
	Sitting time (min/day) on workdays WAO and WFH				
				WAO median (IQR)	WFH median (IQR)
	Time spent sitting during work in min/day			450 (420, 480)	450 (0, 480)
	Total time spent sitting in min/day			705 (630, 863)	641 (510, 847.5)
Scurati et al. (2025) [64]	No difference between accelerometric measured SB (min/week) between hybrid workers and workers only WAO				
	Descriptive results, unadjusted				
	SB of workers WFH and WAO compared to workers only WAO				
				Hybrid	WAO
	Sedentary time (min/week)			8080.8±461.7	8111.8±559.2
Thralls Butte et al. (2023) [67]	SB (min/day) increased in WFH; sit-to-stand transitions decreased in WFH				
	Analysis of variance, unadjusted				
			WFH Mean (SD)	WAO Mean (SD)	Hybrid Mean (SD)
	Self-report				
	Sitting in h/day		6.6 (1.8)	6.5(1.3)	6.6 (0.6)
	Measurement via activPAL				
	Sitting in min/day		569 (111)*	477 (46)*	526 (93)
	Standing in min/day		208 (60)	259 (82)	216 (51)
	Sit-to-stand transitions in no/day		45 (16)	51 (17)	40 (9)
	*p<0.05				
Wallmann-Sperlich et al. (2023) [69]	SB (% sitting during working hours) increased with more WFH; less sitting breaks/h with more WFH				
	Pearson correlation, unadjusted				
	Pearson correlation of SB during working hours and proportion of WFH				
	SB			Pearson correlation coefficient (r)	
	Proportion sitting			0.234*	
	Proportion standing			−0.233*	
	Number of sitting breaks per hour			−0.054	
	*p<0.001				

*Abbreviations*: *b* unstandardised beta coefficient, *CI* Confidence interval, *h* Hours, *min* Minutes, *no* Number, *OR* Odds ratio, *SB* Sedentary behaviour, *SD* Standard deviation, *SE* Standard error, *WAO* Working at office, *WFH* Working from home, *WP* Workplace, β beta coefficient

^1^ results of Barone Gibbs et al. (2021) [34] are not reported due to higher risk of bias compared to Holmes et al. (2023) [10]

^2^ Loef et al. (2022a) [36] addressed our question of interest better than Loef et al. (2022b) [37]

##### Accelerometric assessment of ST

Of the 23 studies examining SB, 13 studies used measurements (e.g., using electronic devices that log movement). Of these, seven studies (“low” risk of bias *n* = 2, “almost low” risk of bias *n* = 4, “high” risk of bias *n* = 1) observed increased ST when WFH compared to onsite work [10, 11, 51, 53, 56, 63, 67], and six studies (“low” risk of bias *n* = 1, “almost low” risk of bias *n* = 3, “high” risk of bias *n* = 2) found no change in ST [9, 48, 52, 64, 68, 71].

##### Office worker

Eleven studies [9, 10, 48, 51–53, 60, 63, 64, 67, 68] included only office or desk workers. Of these, five studies (“low” risk of bias *n* = 1, “almost low” risk of bias *n* = 3, “high” risk of bias *n* = 1) revealed an increased ST when WFH compared to WAO [10, 51, 53, 63, 67] and five studies (“low” risk of bias *n* = 1, “almost low” risk of bias *n* = 2, “high” risk of bias *n* = 2) found no change [9, 48, 52, 64, 68]. One study with “high” risk of bias reported decrease of SB when WFH compared to WAO [60].

##### Studies without pandemic bias

Of the studies considering SB, only six provided data obtained before or after the COVID-19 pandemic [11, 51, 53, 60, 64, 71]. Of these studies, three studies (“low” risk of bias *n* = 1, “almost low” risk of bias *n* = 2) observed an increased ST when WFH compared to WAO [11, 51, 53], two studies (“almost low” risk of bias *n* = 1, “high” risk of bias *n* = 1) reported no change of ST [64, 71], and one study with “high” risk of bias indicated a decreased ST [60] when WFH compared to WAO.

##### Meta-Analysis

**Fig. 2:**
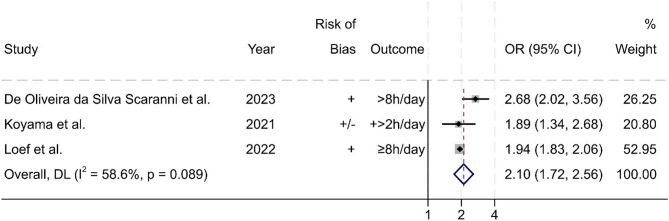
Results of the meta-analysis of odds ratios for sedentary behaviour (*n* = 3). Weights are from random-effects model

A meta-analysis was done with the three studies that reported an estimated OR for either sitting eight or more hours a day [36, 43] or for a daily increase in ST exceeding two hours [55] when WFH compared to usual WP. The meta-analysis estimated a pooled OR of 2.10 (95% CI 1.72 to 2.56; I^2^ = 59%, moderate heterogeneity). This indicates that long sedentary times are two times more likely when WFH compared to usual workplace (Fig. 2).

#### Sitting breaks and sitting bouts

 In total, ten studies [9–11, 46, 53, 63, 67–69, 71] counted sitting breaks (*n* = 7), measured sitting bouts in accumulated sedentary minutes, reported the average length of sitting bouts in minutes, or provided counts of sedentary bouts of varying lengths as outcome parameters.

Tomonaga et al. [11] and Holmes et al. [10] (both with “low” risk of bias) observed fewer sitting breaks when WFH during working hours (sitting breaks/work time) compared to WAO. In contrast, Widar et al. [71] and Sauter et al. [63](both “almost low” risk of bias) reported more sitting breaks when WFH during working hours compared to WAO. Considering an entire day, Thralls Butte et al. [67] (“high” risk of bias) reported fewer sitting breaks (sitting breaks/day) when WFH compared to WAO (not statistically significant), whereas Sers et al. [9] (“almost low” risk of bias) found no difference and the results of Sauter et al. [63] for the entire day showed an increase of STS when WFH compared to WAO. Wallmann-Sperlich et al. [69] (“high” risk of bias) examined the dose-response relationship using sitting breaks per hour, and found the number of sitting breaks decreased with increasing proportion of WFH.

A total of five studies (“low” risk of bias *n* = 1, “almost low” risk of bias *n* = 4) examined sedentary bouts. Holmes et al. [10] (“low” risk of bias) reported more accumulated time spent in long sedentary bouts (SB30 in bouts ≥ 30 min; SB60 in bouts ≥ 60 min) during worktime when WFH compared to WAO. Also, Fukushima et al. [46] (“almost low” risk of bias) reported of averaged longer sedentary bouts during worktime when WFH compared to usual WP. When considering the entire day, Wahlström et al. [68] (“almost low” risk of bias) observed no differences between time spent in short (sitting without interruption for less than 5 min), moderate (sitting without interruption for 5–30 min) or long (sitting without interruption for >30 min) sedentary bouts. Sers et al. [9] (“almost low” risk of bias) found no association between WFH and the number of sedentary bouts of any length (short (≤ 5 min) to long (≥ 40 min) sedentary bouts) during an entire work day. Kitano et al. [53] found increased time in prolonged SB (SB30 in bouts ≥ 30 min), counts of SB-bouts, and longer mean duration of prolonged SB-bouts per day with increased frequency of WFH.

##### Meta-Analysis

**Fig. 3:**
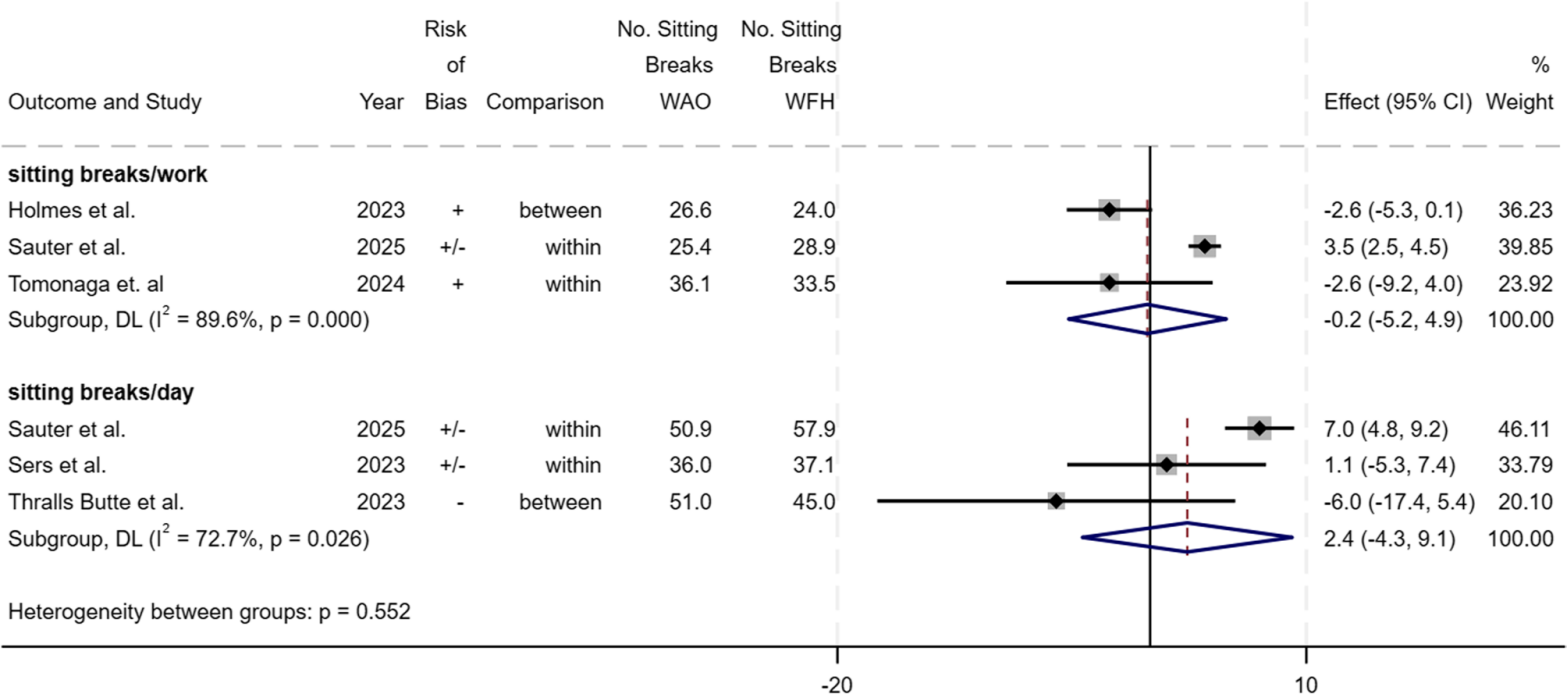
Results of the meta-analysis for sitting breaks/work time (n = 3) and per day (n = 3). Values from within-person and between-person comparisons are indicated in the ‘Comparison’ column. Weights and between subgroup heterogeneity test are from random-effects model. [Deviation of 95% CI from published values possible due to rounding]

 The meta-analysis of the studies of Tomonaga et al. [11], Holmes et al. [10] and Sauter et al. [63] indicated that there is no difference between WFH and WAO in the number of sitting breaks during working hours (sitting breaks/work) (−0.2; 95% CI −5.2 to 4.9; I^2^ = 89.6%), but no statistical significance was reached and heterogeneity was considerable. When the data of Sers et al. [9], Thralls Butte et al. [67] and Sauter et al. [63] were pooled, an estimate of around two more sitting breaks during the entire day (sitting breaks/day) was found for WFH compared to WAO (2.4; 95% CI −4.3 to 9.1; I^2^ = 72.7, substantial heterogeneity) (Fig. 3). The operationalisation of sitting bouts was too diverse to be combined in a meta-analysis.

### Physical activity

Overall, 36 studies examined differences in PA between WFH and onsite work. Of these, four studies had a “low” risk of bias, ten studies had an “almost low” risk of bias, and 22 studies had a “high” risk of bias. In total, 19 studies observed decreased PA when WFH compared to onsite work [9–11, 18, 36, 41, 44, 46, 52, 53, 56–59, 63, 66, 68, 69, 71], ten studies observed no change [40, 42, 43, 45, 48, 54, 61, 64, 65, 70], and five studies observed increased PA when WFH compared to onsite work [38, 47, 49, 62, 67]. Two studies could not identify a clear direction of PA, as PA was increased in one subsample of employees and decreased for the others compared to the usual WP [50] (“high” risk of bias). The other study (“almost” low risk of bias) showed a decrease of light and moderate PA when WFH compared to WAO, but not in vigorous PA [51].

Considering the risk of bias, two of the four studies with a “low” risk of bias showed a decreased PA compared to WAO [10, 11], while the other two found no difference in PA [43, 48] when WFH compared to onsite work. Eight of the ten studies with an “almost low” risk of bias reported decreased PA when WFH compared to WAO [9, 36, 46, 53, 56, 63, 68, 71] when WFH compared to onsite work, while one study reported increased PA [49]. One study reported no clear difference [51]. In the “high” risk of bias category (*n* = 22), nine studies reported decreased PA [18, 41, 44, 52, 57–59, 66, 69] when WFH compared to onsite work, four studies an increase in PA when WFH compared to onsite work [38, 47, 62, 67], and eight studies no difference in PA [40, 42, 45, 50, 54, 64, 65, 70] between WFH and onsite work (Table 4). One study with “high” risk of bias reported no clear difference [50].

**Table 4:** Main results for physical activity (*n* = 36)

Study	Main Results – Physical Activity/Physical Inactivity				
Studies with low risk of bias (*n*=4)					
De Oliveira da Silva Scaranni et al. (2023) [43]	No difference in prevalence of PI (operationalisation based on WHO) in WFH compared to usual WP				
	Logistic regression, adjusted				
	Adjusted OR (95% CI) for PI and domestic PA				
				WFH OR (95% CI)	Usual WP OR (95% CI)
	PI			0.99 (0.75, 1.31)	1 (Ref.)
	Domestic PA			1.29 (0.99, 1.67)	1 (Ref.)
Hallman et al. (2021) [48]	No markedly difference in the distribution of SB, standing and of moving during work or leisure time (moving min/day) between WFH vs. WAO				
	Descriptive analysis, unadjusted				
				WAO days mean (SD)	WFH days mean (SD)
	Total time at work in min/day			512 (165)	486 (205)
	Moving in min/day			37 (17)	36 (27)
	Total leisure time in min/day			468 (128)	461 (159)
	Moving in min/day			70 (34)	62 (30)
	Statistical results of univariate models of compositional data (n= 27) show that behaviours during work and leisure did not change markedly on days WFH compared with days WAO.				
Holmes et al. (2023)^1 ^[10]	Steps/workday decreased in WFH				
	Linear regression, adjusted				
				WAO adjusted β± SE	WFH
	Stepping time (min/workday)			+3.9±2.3	Ref.
	Steps/workday			+695.4±200.5	Ref.
	Note: all *p*-values ≥0.05				
Tomonaga et al. (2024) [11]	PA (MET, time in min standing or in activity) decreased when WFH				
	Multivariate analysis of variance, adjusted				
	Comparison between WFH and WAO including commuting				
				WAO mean^1^ (CI)	WFH mean^1^ (CI)
	Energy consumption in kcal			425.8 (390.0, 461.7)	228.0 (192.1, 263.9)
	Standing or activity time in min			177.0 (161.1, 192.9)	107.2 (91.3, 123.1)
	^1^ indicated as least squares mean				
Studies with almost low risk of bias (*n*=10)					
Fukushima et al. (2021) [46]	PA (workplace activity questionnaire) decreased when WFH compared to usual WP				
	Analysis of covariance, adjusted				
	Time spent in each behaviour during working time				
			Usual WP mean (SE)	WFH mean (SE)	*p*-value
	Light PA in min		146.5 (12.8)	97.3 (13.7)	<0.001
	Moderate to vigorous PA in min		118.0 (10.3)	90.7 (11)	<0.001
Henke et al. (2016) [49]	Tendency for decreased PI (defined as cardiovascular exercise less than 3x/week) among WFH, that means an increase in PA				
	Logistic regression, adjusted				
	PI				
				Beta	OR^a^
	Off-hour WFH			−0.122	0.89
	Prime time WFH		Low (≤8h/month)	−0.189	0.83
			Medium (9-32h/month)	−0.249*	0.78
			High (33-72h/month)	−0.140	0.87
			Very high (≥73 h/month)	−0.015	0.99
	WAO			Reference	
	*p<0.05 ^a^ORs were self-calculated; neither 95% CI nor SE were published				
Kikuchi et al. (2025) [51]	No clear direction: PA (steps/day, LPA, MPA in min/day) decreased, while VPA (min/day) increased when WFH compared to WAO				
	Descriptive results, intraindividual comparison				
				Days WAO	Days WFH
	PA				
	Average of step count per day (SD)			8046 (2586)	3284 (2908)
	LPA in min/day (SD)			197 (70.0)	156.1 (79.1)
	MPA in min/day (SD)			60.7 (20.9)	27.2 (23.9)
	VPA in min/day (SD)			1.6 (4.5)	2.5 (7.7)
Kitano et al. (2024) [53]	PA (LPA, MVPA and total PA in min/day, steps/day) decreased with increasing WFH-frequency				
	Multiple linear regression, adjusted Estimated marginal means with 95% CI				
		Never WFH	1–2 days WFH	3–4 days WFH	≥5 days WFH
	PA				
	LPA (min/day)	251 (241–260)	215.3 (203–228)	208 (195–221)	205 (194–216)
	MVPA (min/day)	55 (52–58)	49.7 (46–54)	37.3 (33–42)	28 (24–32)
	Total PA (min/day)	305.6 (295–316)	265.1 (252–279)	246 (231–260)	233 (221–246)
	Step counts (steps/day)	7200 (6856-7544)	6214.5 (5751-6678)	4414 (3934-4893)	3208 (2790-3625)
Leskinen et al. (2025) [56]	PA (min/day) decreased when WFH				
	Generalised linear models and linear mixed models, adjusted				
	Daily physical activity time for workdays by the work mode group of non-manual workers				
			Usual WP	Hybrid	WFH
	PA		Mean (95% CI)	Mean (95% CI)	Mean (95% CI)
	Total PA (min/day)		387 (330–444)	347 (290–405)*	324 (259–389)*
	Occupational PA (min/day)		412 (349–475)	346 (283–409)*	330 (259–400)*
	Non-occupational PA (min/day)		193 (137–249)	137 (81–194)*	143 (79–207)*
	Daily physical activity for office and remote workdays among hybrid workers				
				Days WFH	Days WAO
				Mean (95% CI)	Mean (95% CI)
	Total PA (min/day)			362 (328–397)	389 (355–424)
Loef et al. (2022a)^2 ^[36]	PA (moderate to vigorous PA ≥150 min/week) decreased if WFH				
	Logistic generalized estimating equations analysis, adjusted values				
	Moderate to vigorous PA ≥150 minutes during pandemic				
				OR (95% CI)	
	WFH			0.93 (0.90, 0.96)	
	Hybrid workers			1.02 (0.98, 1.07)	
	Usual WP			1 (Ref.)	
Sauter et al. (2025) [63]	Decreased number of steps (steps/day and steps/work time) when WFH compared to WAO				
	Linear mixed models, adjusted				
	Adjusted number of steps (based on own data)				
				Days WFH (95% CI)	Days WAO (95% CI)
	Steps in counts/work			2027 (1616–2439)	3515 (3105-3925)
	Steps in counts/day			7434 (6188-8681)	9454 (8203–10705)
Sers et al. (2023) [9]	PA (time in moderate to vigorous PA, steps, MET) decreased when WFH (but number of short PA bouts increased)				
	Descriptive analysis and multilevel model analyses, adjusted				
				Days WAO mean (SD)	Days WFH mean (SD)
	Steps (no/day)			7548 (3944)	6299 (3779)
	Models of PA-related outcomes			WFH	
				B (SE)	
	Light PA time (h/day)			0.09 (0.15)	
	Moderate to vigorous PA time (h/day)			−0.30 (0.08)**	
	PA intensity (MET per day)			−0.05 (0.02)*	
	Steps (no/day)			−1288.1 (449.0)*	
	Short PA-bouts (no/day)			3.60 (1.32)*	
	Short-to-moderate PA-bouts (no/day)			−0.87 (0.64)	
	Moderate-to-long PA-bouts (no/day)			−0.62 (0.23)**	
	Long PA-bouts (no/day)			0.07 (0.16)	
	Note: *p<0.05; **p<0.01.				
Wahlström et al. (2023) [68]	PA (ratio for time not sitting/time sitting) decreased when WFH				
	Multilevel linear mixed models, post-hoc pairwise comparison between WAO and WFH, adjusted				
				NonSit/Sit (ILR2)	
	Day type			B (95% CI)	
	WAO			−0.19^a^ (−0.26, −0.11)	
	WFH			−0.28(−0.37, 0.19)	
	NWD			Ref.	
	^a^significant difference (*p*<0.05) in pairwise comparison of WAO and WFH				
Widar et al. (2021) [71]	PA (walking, running, cycling in min/day) decreased over the entire day when WFH (but increased during work hours)				
	Descriptive analysis, unadjusted				
				WFH mean^1^	WAO mean^1^
	Walking, running, cycling during work in min/day			66	51
	Walking, running, cycling for the total time in min/day			135	143
	^1^geometrical mean; SE not reported				
Studies with high risk of bias (*n*=22):					
Abed Alah et al. (2022) [40]	DPA no difference between WFH and usual WP				
	Mann-Whitney U-Test, unadjusted				
	Exercise time difference (h/day) mean ranks				
	WFH: 536.3; usual WP: 525.4 p=0.537				
Bérard et al. (2021, 2022) [41]	DPA (min/week) decreased when WFH compared to usual WP				
	Logistic regression, adjusted values				
				WFH during lockdown	Usual WP during lockdown
	Adj. OR (95% CI)			1.96 (0.69, 5.58)	Ref.
Cobbold et al. (2024) [42]	No difference in change of total PA between those who worked “less or same” and “more” WFH compared to pre-pandemic WFH				
	Comparison of adjusted estimates of a mixed-effects model				
	WFH compared to pre-pandemic			Wave	Weekly total DPA in min estimate (CI)
	Less or same			2019	Reference
				2021	+46.4 (−30.0, 122.8)
	More			2019	Reference
				2021	+45.3 (5.5, 85.1)
	No 95% CI reported for comparison between “less or same” and “more” group in 2021 (+46.4 vs. +45.3)				
Delanoeije et al. (2024) [44]	PA (PA score) decreased on WFH days compared to days at the usual WP				
	Linear mixed models, adjusted				
	Physical activity score decreased on days WFH (ß=−29.96; *p*<0.001)				
Elangovan et al. (2021) [45]	DPA (exercise/week); no difference in PA				
	Pearson`s Chi-square test, unadjusted				
	Exercise/week			WFH (%)	WAO (%)
	Within 3h			41.2	36.2
	3-7h			10.5	9.7
	>7h			5.5	4.4
	did not do any activity			42.8	49.7
	Chi-square p=0.191, Cramer’s (V)=0.068				
Grubben et al. (2022) [47]	PA: increase in sports participation per increase in hour of WFH per work week				
	Logistic regression, adjusted values				
	Beta = 0.009* SE =0.004				
	Adj. OR 1.01 (95% CI 1.00, 1.02) per hour WFH/week Adj. OR 1.09 (95% CI 1.08, 1.10) per 10 hours WFH/week				
	*results converted to OR (95% CI) by the authors				
Herbolsheimer et al. (2024) [50]	PA no clear direction				
	Multivariable linear and log-binomial regression models, adjusted				
	WFH was related to increased engagement in household chores (β = 0.16; 95% CI 0.159, 0.171), and more recreational activities (β=0.05; 95% CI 0.043, 0.056), employees shifting to WFH, who achieved the WHO guideline pre-pandemic were significantly more likely to fall below these guidelines (OR=2.17; 95% CI 2.04, 2.31), but employees, who did not achieve the guidelines have a higher chance to fulfil those when working remote (OR= 1.47, 95% CI 1.39, 1.56)^a^				
	^a^personal communication				
Ishibashi et al. (2022) [18]	PA: Commuters (usual WP) have much more PA associated with daily trips than teleworkers (WFH) do, irrespective of the time point (before, during, after the pandemic)				
	Descriptive analysis				
Kim et al. (2022) [52]	PA (min/day) – tendency for decrease in WFH; steps/day decreased in WFH				
	Descriptive analysis, unadjusted				
	PA at baseline for days WAO and WFH				
				WAO mean (SD)	WFH mean (SD)
	Light PA, min/day			179.7 (72.8)	175.9 (68.6)
	Moderate to vigorous PA, min/day			58.5 (27.5)	34.1 (33.9)
	Steps, no/day			8215 (2681)	4261 (3526)
Javad Koohsari et al. (2021) [54]	No association between changes of most PA-categories and absolute change of days WFH (exception work-related moderate PA)				
	Multivariable linear regression models, adjusted				
			Work-related vigorous PA (h/day)	Work-related moderate PA (h/day)	Total PA (h/day)
			b (95% CI) *p*-Value	b (95% CI) *p*-Value	b (95% CI) *p*-Value
	WFH (days/week)		0.02 (−0.01, 0.05)	−0.06 (−0.10, −0.02)*	−0.04 (−0.11, 0.03)
	*p<0.05.				
Marenus et al. (2025) [57]	PA (MET-min/week) decreased when WFH				
	Descriptive results, unadjusted				
	Average scores of PA in MET-min/week by remote status				
			Usual WP	Hybrid	WFH
	PA				
	Vigorous PA (SD)		954 (1601)	934 (1130)	852 (1438)
	Moderate PA (SD)		631 (991)	413 (478)	426 (840)
	Walking (SD)		903 (1061)	622 (642)	536 (614)
	Total PA (SD)		2489 (2949)	1970 (1700)	1792 (2233)
Massar et al. (2023) [58]	PA (daily step count) decreased when WFH				
	Linear mixed-effects models, adjusted				
	WFH was associated with lower daily step count compared to the usual WP (−2,471 steps/day).				
Matthews et al. (2022) [59]	PA (different PA behaviours) decreased when WFH				
	Descriptive analysis, unadjusted				
	Pre-pandemic PA from 2019				
				WFH mean	usual WP mean
	Total PA in h/day			5.09	6.99
	Light PA in h/day			3.71	3.86
	Moderate-vigorous PA in h/day			1.39	3.13
	SE not reported				
Moura et al. (2022) [38], Moura et al. (2023) [39]	PI (yes/no) during leisure time decreased when WFH, that means that PA increased when WFH				
	Multivariate logistic regression, adjusted OR (95% CI)				
				March – Aug. 2020 adjusted OR (95% CI)	Oct. – Dec. 2020 adjusted OR (95% CI)
	Usual WP			1.00 (Ref.)	1.00 (Ref.)
	No work			1.29 (0.77, 2.20)	0.97 (0.62, 1.51)
	WFH			0.52 (0.30, 0.89)	0.49 (0.28, 0.84)
	Moura (2022) Moura (2023) reported an adjusted OR of 1.86 for PI (95% CI 1.08–3.23.08.23, b=0.62; 95% CI 0.08–1.17.08.17) for individuals working at the usual WP compared to those reported full or partial WFH.				
Oxenham et al. (2025) [61]	No difference after having starting work in PA (MET-min/day) between WFH and usual WP				
	Descriptive result, adjusted				
	Initial difference after transitioned into work (WFH: 126.42 MET-min/day, 95% CI: −264.45 to 11.61; usual WP: ß=128.81 MET-min/day, 95% CI: 89.46 to 168.16) between WFH and work at the usual WP was not maintained over years.				
Prince et al. (2024) [62]	Higher chance to fulfil Canadian MVPA-recommendations when WFH compared to the usual workplace				
	Logistic regression, adjusted				
	Compared to working at the usual workplace, the chance to fulfil Canadian MVPA-recommendations is 19% higher (OR 1.19 (95% CI 1.00, 1.41)).				
Scurati et al. (2025) [64]	No difference in accelerometric measured PA (LPA, MPA, VPA and total PA min/week) between hybrid workers and workers only WAO				
	Descriptive analysis, unadjusted				
	PA of hybrid worker and WAO				
	Variables			Hybrid	WAO
	Vigorous activity (min/week)			31.4±49.4	19.7±46.8
	Moderate activity (min/week)			940.8±346.8	964.9±428.3
	Light activity (min/week)			562.0±125.2	520.2±123.6
	Total activity (min/week)			1534.2±446.4	1504.8±544.9
Silva et al. (2021) [65]	No difference in PI (PI≤150 min/week PA) in WFH (during and before the pandemic) compared to usual WP				
	Descriptive analysis, unadjusted				
	PI prevalence (95% CI)				
				Before pandemic % (95% CI)	During pandemic % (95% CI)
	Usual WP			70.6% (67.3, 73.6)	88.9% (86.5, 90.9)
	WFH			69.2% (66.7, 71.6)	86.6% (84.6, 88.4)
Suzuki et al. (2025) [66]	Higher proportion of self-rated decrease of PA when WFH compared to usual WP				
	Descriptive analysis, adjusted				
	60.9% of the WFH-group indicated a decrease of PA compared to 29.7% of the workers at the usual WP stated a decrease of PA				
Thralls Butte et al. (2023) [67]	PA (min/day), steps (no/day) and stepping (min/day) increased in WFH				
	Analysis of variance, unadjusted				
			WFH mean (SD)	WAO mean (SD)	Hybrid mean (SD)
	Self-report				
	Aerobic PA in min/day		103 (51)	81 (69)	150 (136)
	Muscle PA in min/day		2.2 (2)*	0.5 (1.2)*	2.0 (1.9)
	ActivPAL				
	Steps in no/day		7289 (3317)	5984 (1556)	7766 (2883)
	Stepping in min/day		87 (36)	78 (20)	88 (25)
	*p<0.05				
Wallmann-Sperlich et al. (2023) [69]	Walking during work (% of working hours) and physically demanding work tasks (% of working hours) decreased with more WFH				
	Pearson correlation, unadjusted				
	Pearson correlation of PA during working hours and proportion of WFH				
	PA behaviour			Pearson correlation coefficient (r)	
	Proportion walking			−0.130**	
	Proportion physically demanding work tasks			−0.076	
	**p<0.01				
Webber et al. (2023) [70]	No difference for PA (≥150 min moderate to vigorous PA per week) related to change of WFH				
	Log-binomial regression, adjusted				
	Meeting aerobic guidelines			Prevalence in % (95% CI)	Adjusted PR (95% CI)
	Never WFH			48.4 (44.3, 52.5)	1.00
	More WFH			60.0 (55.8, 64.1)	1.06 (0.97, 1.17)
	Same WFH			56.6 (51.4, 61.8)	1.07 (0.97, 1.18)
	Less WFH			51.7 (43.1, 60.3)	1.05 (0.90, 1.21)

*Abbreviations*: *MET* Metabolic equivalent, *h* hours, *kcal* kilocalorie, *min* minutes, *no* number, *NWD* Non-working day, *OR* Odds ratio, *PA* Physical activity, *PI* Physical inactivity, *SD* Standard deviation, *SE* Standard error, *WAO* Working at office, *WFH* Working from home, *WP* Workplace, *β* beta coefficient

^1^ results of Barone Gibbs et al. (2021) [34] are not reported due to higher risk of bias compared to Holmes et al. (2023) [10]

^2^ Loef et al. (2022a) [36] addressed our question of interest better than Loef et al. (2022b) [37]

#### Accelerometric assessment of the outcome

Although, 14 studies used accelerometers to measure PA, this resulted in varied outcomes, such as time spent moving or in light/moderate PA or “daily walking, running or cycling”, number of steps or energy consumption in METs. Ten studies [9–11, 52, 53, 56, 58, 63, 68, 71] are in favour for decreased PA when WFH compared to onsite work, two studies [48, 64] found no markedly difference between WFH and WAO, and one study reported [67] increased PA when WFH compared to WAO. One study with “almost low” risk of bias showed no clear direction when comparing WFH with WAO [51].

#### Office worker

Regarding the composition of the working populations, 12 studies included only office or desk workers. Of these, seven studies reported decreased PA when WFH compared to onsite work [9, 10, 44, 52, 53, 63, 68], two studies reported increased PA when WFH compared to WAO [49, 67], and two study reported no markedly difference in PA between WFH and WAO [48, 64]. One study showed a decrease of light and moderate PA and an increase of vigorous PA when WFH compared to WAO [51].

#### Studies without pandemic bias

Six studies provided information on PA differences outside of the pandemic situation (“low pandemic bias”). Three studies observed a decrease in PA when WFH compared to WAO [11, 53, 71], one study indicated no difference between WFH and WAO [64], and one study observed an increase of PA when WFH compared to WAO [49]. One study showed no clear direction [51].

#### Meta-Analysis

**Fig. 4:**
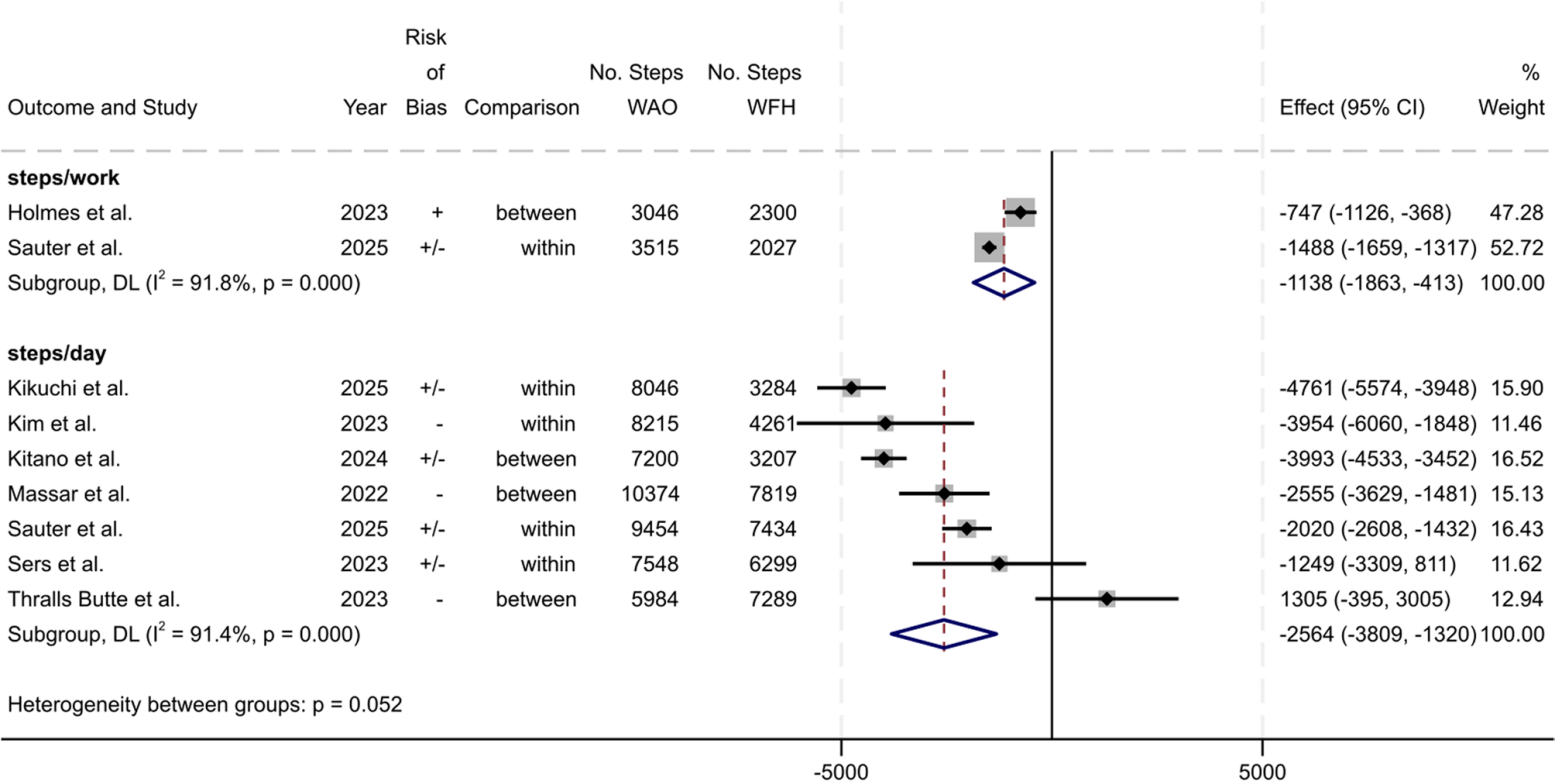
Results of the meta-analysis for steps/work time (*n* = 2) and per day (*n* = 7). Values from within-person and between-person comparisons are indicated in the ‘Comparison’ column. Weights and between subgroup heterogeneity test are from random-effects model. [Deviation of 95% CI from published values possible due to rounding]

The meta-analysis of the seven studies [9, 51–53, 58, 63, 67] that measured the number of steps for the entire day (steps/day) resulted in a pooled mean difference of 2564 fewer steps (mean difference: −2564; 95% CI −3809 to −1320; I^2^ = 91.4%, considerable heterogeneity) when WFH compared to onsite work. Holmes et al. [10] and Sauter et al. [63] measured steps during working hours (steps/work time). Meta-analysis of these two studies estimated 1138 fewer steps (mean difference: −1138; 95% CI −1863 to −413; I^2^ = 91.8%, considerable heterogeneity) when WFH compared to WAO (Fig. 4).

### Dose-response relationships

Eleven studies provided information on possible dose-response relationships. The studies shown in Table 5 examined if the amount or proportion (WFH only, hybrid work) of WFH is associated with the amount of ST (*n* = 7) or PA (*n* = 11). With regard to ST, five studies reported a positive association [36, 46, 53, 54, 69] between WFH and ST, while two studies found no association [56, 67].

**Table 5:** Dose-response relationships between working from home and sedentary behaviour (*n* = 7) and physical activity (*n* = 11)

Author (Year)	Sedentary behaviour	Physical activity	Risk of bias
Fukushima et al. (2021) [46]	Longer sitting time during work in the highest WFH group (76%−100%)	Shorter light PA and moderate to vigorous PA in the highest WFH group (76%−100%)	+/-
Grubben et al. (2022) [47]	-	More sports participation per hour WFH	-
Kitano et al. (2024) [53]	Positive association between WFH frequency and time spent in SB, prolonged SB and daily counts of prolonged SB bouts.	Negative association between WFH frequency and time spent on MVPA, total PA and steps.	+/-
Koohsari et al. (2021) [54]	Positive association between change of work-related SB, total SB (h/day) and absolute change of days WFH	No association between absolute change of days WFH and PA domains, but a negative association between work-related moderate PA	-
Leskinen et al. (2025) [56]	Compared to onsite workers, hybrid and remote workers spent less time during work and during the entire workday sedentary. No difference was found between hybrid and remote workers	Compared to onsite workers, hybrid and remote workers spent less time on PA during work and during the entire workday. No difference was found between hybrid and remote workers.	+/-
Loef et al. (2022a; 2022b) [36] [37]	Compared to workers at the usual workplace, WFH workers and to lesser extent hybrid workers were more often sedentary	Compared to workers at the usual workplace, WFH workers and to lesser extent hybrid workers were more often physical inactive	+/-
Marenus et al. (2025) [57]	-	Compared to workers at the usual WP, hybrid and WFH workers spent less time with PA during the entire day. No difference was found between hybrid and WFH workers	-
Moura et al. (2023) [39]	-	No linear association between the amount of WFH and the chance to PI	-
Scurati et al. (2025) [64]	-	Positive correlation between percentage of WFH and vigorous PA	-
Thralls Butte et al. (2023) [67]	No differences in SB between those who worked hybrid and those who worked exclusively from home	No differences in PA between those who worked hybrid and those who worked exclusively from home	-
Wallmann Sperlich et al. (2023) [69]	WFH proportions are positively related to higher workday (working hours) sitting	WFH proportions are negatively related to workday (working hours) walking	-

*Abbreviations*: *PA* Physical activity, *PI* Physical inactivity, *SB* Sedentary behaviour, *WFH* Working from home

Four of the 10 studies considering PA reported a negative association between WFH and PA [36, 46, 53, 69], while five found no association [39, 54, 56, 57, 67], and two studies reported a positive association [47, 64] between WFH and PA (sports participation and vigorous PA).

## Discussion

Overall, our results indicate that WFH was associated with a potentially unhealthy increase in SB and decrease in PA compared to onsite working. The odds of long(er) ST were doubled and about two fewer sitting breaks during work time were taken when WFH compared to onsite work. Further meta-analyses with high heterogeneity also indicated that WFH was associated with an over 30-minute increase in ST and around 2500 fewer steps per day when compared to onsite work.

**Fig. 5:**
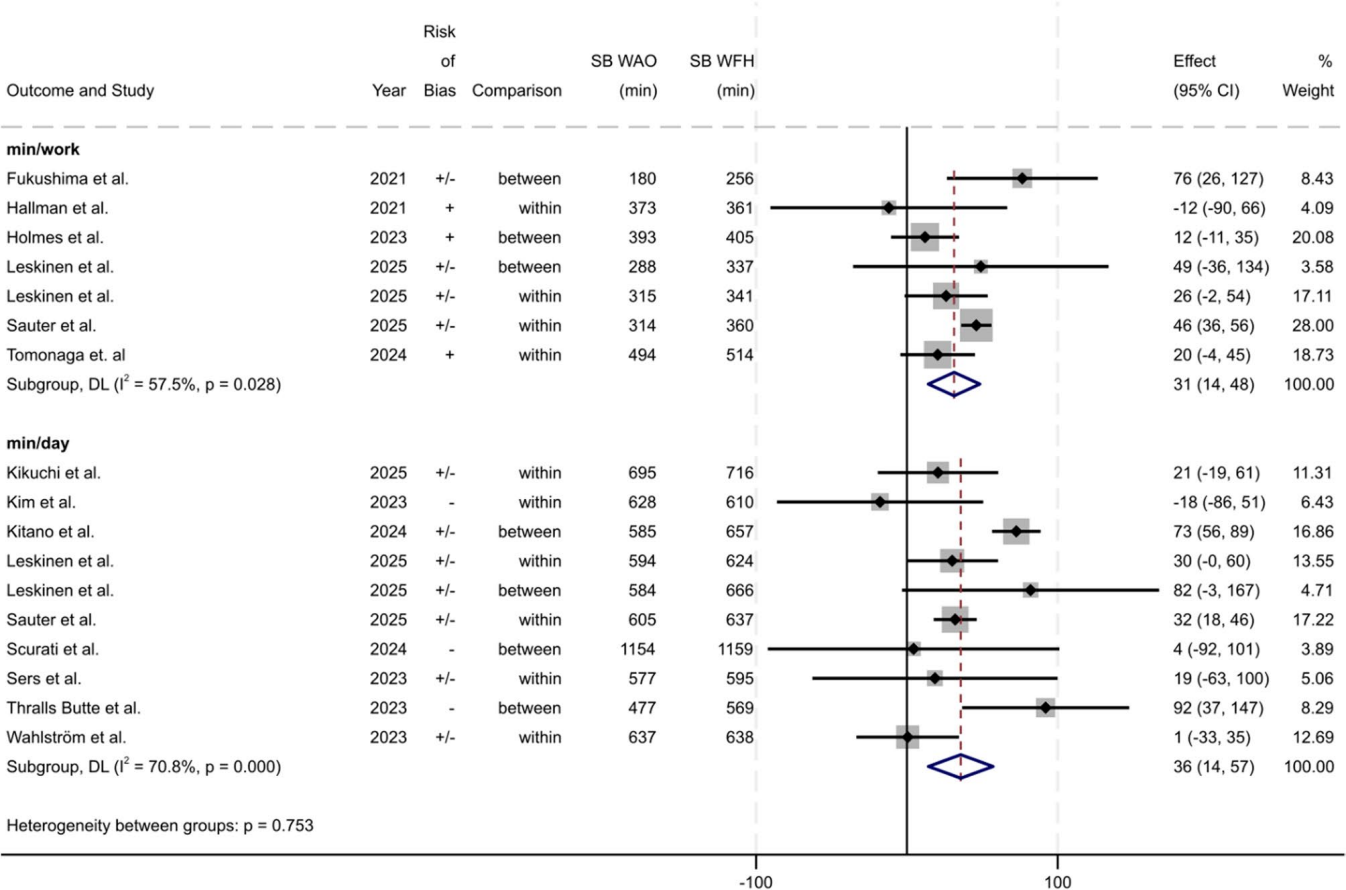
Results of the meta-analysis for sedentary time/work time (n = 6 studies) and per day (n = 9 studies). Values from within-person and between-person comparisons are indicated in the ‘Comparison’ column. Weights and between-subgroup heterogeneity test are from random-effects model. [Deviation of 95% CI from published values possible due to rounding]

The results of the included studies tended to find longer ST on days WFH compared to onsite work. Also, our meta-analysis found the chance for being sedentary for over eight hours per day or increasing ST by two or more hours was doubled when WFH compared to onsite work. The meta-analysis also found mean ST increased by about half an hour when WFH compared to onsite work during work time (31 min, 95% CI 14 to 48; I^2^: 57.5%). According to Blodgett et al. [72], the replacement of about 30 min of moderate PA, light PA, standing or sleep with SB negatively influences health parameters like body max index or high density lipoprotein cholesterol [72]. The estimates of most of the studies included in the meta-analysis found differences less than one hour of ST with exception of Fukushima et al. [46], Thralls Butte et al. [67], Leskinen et al. [56] (interindividual comparison) and Kitano et al. [53] which reported an increase of over one hour when WFH compared to onsite work (Fig. 5). These higher estimates might be explained by different job task (e.g., essential workers) of the comparison group [46, 56], differences in person-related factors (e.g., health status/behaviour) of workers working fulltime from home compared to fulltime onsite workers [53, 67]. Only the study by Kim et al. [52] reported ST/day decreased by around 18 min (95% CI −67 to 31) on days WFH compared to onsite work (WAO) and the study by Hallman et al. [48] found a decrease of ST/work when WFH compared to onsite work (WAO) by about 12 min (95% CI −67 to 42).

Seven studies were rated with low pandemic bias: Olsen et al. [60] observed approximately one hour less ST when WFH compared to WAO (descriptive result). In contrast, Widar et al. [71] found no difference in ST between the two settings. Both studies were conducted prior to the COVID-19 pandemic. Post-pandemic, however, Tomonaga et al. [11], Kitano et al. [53] and Kikuchi et al. [51] reported an increase in ST when WFH compared to onsite work (WAO) for Japanese office workers. Additionally, Kitano et al. [53] reported an increase prolonged ST, prolonged SB-bout duration and increased number of SB-bouts. An Italian study of Scurati et al. [64] reported no differences in accelerometric ST (min/week), but increased self-reported ST (min/day) when WFH compared to WAO (not statistically significant). Pre-pandemic data from Henke et al. [49] showed a trend towards increased cardiovascular exercise when WFH compared to onsite work (WAO). While, Widar et al. [71] observed a reduction in daily walking, running, or cycling time when WFH compared to onsite work (WAO), yet recorded an increase in active time during working hours. Additionally, post-pandemic data from Tomonaga et al. [11] revealed: a marked decrease in energy expenditure and a reduction in activity time, including standing, when WFH compared to onsite work (WAO). Kitano et al. [53] and Kikuchi et al. [51] observed a decrease in light and moderate PA and number of steps per day when WFH compared to onsite work (WAO). Interestingly, Kikuchi et al. [51] found that, vigorous PA slightly increased when WFH compared to WAO. This was not reported by Kitano et al. [53], whereas total time with PA decreased with increasing WFH frequency. Also, post-pandemic subjective data of Scurati et al. [64] also revealed more vigorous PA in hybrid workers than in onsite workers, but this was not confirmed by their accelerometric data. Since there are only four studies with post-pandemic data, with the majority performed in Japan, there is a clear need for further research also in other countries.

Some studies compared office workers with the general working population. The results of these studies may be confounded by the inclusion of non-office workers who are less likely to be able to perform WFH and who may have more physically active work. This might have led to an overestimation of the effect of WFH. However, two studies [46, 55] accounted for this source of confounding in their statistical analysis.

Operationalisation of patterns of SB other than ST (e.g., sitting breaks or sedentary bouts) was very heterogenous, which made it difficult to compare the results. The reported results for sitting breaks were unclear. Results of our meta-analyses, each based on three studies, showed more sitting breaks during the entire day when WFH compared to onsite work (WAO), but no difference during work time between the workplaces. Results were not statistically significant and heterogeneity was high. Sedentary bouts tended to be longer when WFH compared to onsite work, although some studies suggested no difference between workplaces. Sedentary bouts, reported in the studies of Holmes et al. [10], Fukushima et al. [46], Sers et al. [9], Wahlström et al. [68] and Kitano et al. [53] could not be included in the meta-analysis due to heterogenous operationalisation.

Further research should focus on collecting post-pandemic data, preferably from office workers, using accelerometric measurements or validated questionnaires on PA and ST that can also distinguish between PA intensities (light, moderate, and vigorous PA). This distinction and consideration of potential subgroups (e.g., physically inactive individuals) can be important for a more differentiated assessment of workers’ health behaviours when working from home and/or at the office. When, using accelerometric data, including SB variables such as mean sedentary bout length and number of sit-to-stand transitions is recommended. From an occupational health perspective distinction between work time and leisure time seems to be important. In the event that data have been collected from the general working population, it is imperative that this be taken into consideration during the data collection and analysis process. This will allow for the adjustment of potential bias.

Most of studies investigating dose-response relationships between the frequency of WFH and SB suggest more time WFH is positively associated with ST. This needs to be considered by both employees and employers when planning time at different workplaces. Since flexibility to work at different workplaces has advantages (e.g., reduced work privacy conflict [73]).

Our meta-analysis found indications that WFH decreased steps per day by about 2500 steps per day when compared to onsite work (−2564; 95% CI −3809 to −1320; I^2^ = 91.4%). The effect for PA in general is less clear and the operationalisations of the included studies are more heterogenous. This also pertains to the results of studies investigating a dose-response relationship between the amount of WFH and PA. The mixed findings may be explained by the heterogeneity of the definitions and measurement of PA. Assessment of PA varied between accelerometric measurements, validated questionnaires, and single item variables (e.g., self-reports on sport participation without time or intensity estimates). For example, Scurati et al. [64] reported differences between hybrid and onsite workers based on subjective data. This result was not confirmed by accelerometric measurement in this study [64]. Due to differences in physical abilities and preferences, the physical behaviour of individuals may vary more than their SB, and it is difficult to adequately summarise such heterogeneous behaviour in a single summary parameter for physical behaviour. As shown in Herbolsheimer et al. [50] there might be subgroups of employees who profit from the possibility of WFH and others who do not. Regional and seasonal differences may also account for some of the heterogeneity of results. Commuting by bike, bus, or train can contribute to daily physical movement but varies by region and season [74].

Possible reasons for longer ST and less movement when WFH may be longer worktimes, typically shorter distances [46], and the lack of the possibility to meet colleagues in person [19]. Another reason may be the decision of employees to WFH when the tasks are (more) suitable to this environment (e.g., no meetings with clients, only desk-based tasks, high level concentration tasks [75]) resulting in longer ST compared to onsite work. Also, employees may feel social pressure to be even more productive when WFH. This can result in an unhealthy sitting pattern, based on the idea “When I work in a concentrated manner, I have to sit.” [19]. Also, WFH eliminates the need to commute to the workplace. Active commuting increases PA and is associated to a reduction of all-cause mortality [76].

The results of Wilms et al. [20], correspond with ours and also point to longer SB and less PA when WFH compared to onsite work. From the studies included in our review, only the study of Fukushima et al. [46] was included in the review of Wilms et al. [20]. The results of the review may be more directly the results of pandemic restrictions. The results of the meta-analysis of Chaudhary et al. [21] also support our results and describe a significant decrease in PA (mild effect size; Hedge’s g=−0.29; 95% CI −0.41 to −0.18) and an increase in SB (mild effect size; Hedge’s g = + 0.36; 95% CI 0.20 to 0.52) when transitioning to WFH due to COVID-19 pandemic. Also, the studies included by Chaudhary et al. [21] overlap only partially with the studies considered in our review [34, 46, 48] due to the slightly different research questions and search periods. The review of Polspoel et al. [22] only found significant decrease of light PA (mild effect size; Hedge’s g=−0.33; 95%CI −0.59 to −0.08) and no change were observed for total PA, moderate and vigorous PA and SB [22] when WFH during the pandemic. Last search of Polspoel et al. [22] was performed in January 2024 as they focused on the time during COVID-19. This resulted in an overlap of six studies with our review [34, 43, 46, 48, 54, 71]. Furthermore, they considered different PA outcomes (light, moderate and vigorous PA and ST), but no further variables describing SB. Moreover, the systematic review by Sers et al. [23] reported evidence of a negative association between WFH and PA, which is consistent with the findings of our review. In contrast, their results indicated no association between WFH and SB. Sers et al. performed their search in April 2023 and focused on healthy working adults. Therefore, an overlap of five included studies [48, 54, 60, 67, 71] exists. As observed in our systematic review, the majority of the included studies were rated as low quality based on risk of bias assessments.

If future studies can confirm that WFH does indeed reduce the number of steps taken per day by about 2500 steps per day and increase ST by about 30 min per day, this change could be detrimental to health. According to Banach et al. [77] all-cause mortality is reduced about 15% with an 1000-step increment per day and cardiovascular mortality is reduced about 7% with an increase of already 500 steps per day [77]. They also reported that health benefits already appear with a cut-off points of 2337 steps per day for cardiovascular mortality and 3967 steps per day for all-cause mortality. Together with an increase in SB on days WFH, this would increase two risk factors for non-communicable diseases [12, 78]. In addition, when ST is high, the time to compensate for this with PA is limited, even though the need is higher [12].

### Strength, limitations and weaknesses of the review

This systematic review used a comprehensive search and meta-analysis to summarise the body of published results on a current but not yet well researched topic. The primary studies represented a variety of countries with cultural differences in sitting and movement behaviours. We conducted meta-analyses of the reported results, including the objectively measured outcomes, without converting to standardized effect sizes such as Hedges’ g. This provides a more precise and intuitive understanding of how WFH impacts SB and PA. A further strength of the review was the assessment tool developed for the review, which was adapted to the topic and worked for the different study designs included. This assessment tool also took into account that pandemic restrictions may limit the generalisability of some of the primary studies to “non-pandemic” working conditions. We were able to include seven studies without pandemic bias as these studies were either performed before (*n* = 3) or after (*n* = 4) the COVID-19 pandemic. The differential consideration of these studies helps to better understand the influence of WFH on SB and PA independent of pandemic restrictions.

However, most of the studies were performed during pandemic and understanding how WFH impacts SB and PA is challenging. Pandemic conditions may have otherwise influenced SB and PA. Restrictions or fear of infections may have impacted typical onsite work behaviours (e.g., eating with co-workers) and leisure time activities (e.g., team sports, fitness studio visits). Therefore, the generalizability of our results is limited. In addition, the study questions of the “pandemic studies” did not always align well with our study questions. Thus, the extracted data from these studies were often limited to descriptive results or results that did not adjust for confounding factors relevant to our research question. When this was the case, it was reflected in a higher risk of bias rating.

Another challenge, was that many studies investigated not only desk workers, who had the possibility to WFH, but the entire working population. These included blue collar workers, service industry workers, and nurses who usually could not WFH. This may have confounded the results if the type of work was not taken into account in the statistical analyses, as the place of work and the type of work may differ in terms of ST and PA. Thus, the effect of WFH on SB may be overestimated in these studies. In addition, the measurement of WFH varied between studies. While the majority of studies considered WFH as a binary outcome, others attempted to capture the transition to WFH due to pandemic. Few studies quantified the amount of WFH. This heterogeneity and potential misclassification may have affected the results.

Also, the heterogeneity of investigated outcomes and study designs made it difficult to synthesize the study results. We managed to perform meta-analysis for four outcomes with at least two studies, in accordance with Cochrane recommendations [79]. However, in the case of Koyama et al. [55], the outcome differed as they reported an OR for an increase of two or more hours of sitting when WFH instead of an OR of sitting eight or more hours per day. However, their results were consistent with the results of the studies considering the relative odds of sitting eight or more hours per day. Excluding Koyama et al. [55] from the meta-analysis also results in similar results (OR 2.21; 95% CI 1.62 to 3.02). Furthermore, the measure of heterogeneity via I^2^ is challenging in meta-analyses with few studies [80].

### Practical implications

The workplace is an important setting for effective interventions, so it is essential to increase awareness in employees and employers that working environments can influence sitting and moving patterns and thus impact health. For example, Holmes et al. [10] found workers with better access to break rooms or common areas at the workplace took 500 more steps and spent six additional minutes walking when working onsite.

Even after the end of the pandemic situation, many companies and workers continue to use WFH and teleworking to reduce commuting times and promote the balance of work and private responsibilities [81]. Despite the trend of some employers to bring their employees back to the workplace, it is hard to imagine office work without remote/hybrid working options [81, 82]. However, interventions to reduce ST at work have typically been developed for traditional office settings and need to be adapted for WFH. Thus, it is crucial that occupational, safety and health professionals and stakeholders think about the adaptation of workplace health promotion to the home environment [83]. Employees should be advised to use the time they gain by not travelling to work to be physically active as described by Schmidt et al. [83]. They should also keep in mind, that the personal needs for PA vary among employees (e.g., some persons might already use the gained time for physical exercise and others not).

Employees can also be supported by changes of the workplace [84–86] and work organisation [87]. For example, work can be organised in a way that the health promoting offers, such as guided “exercise/stretching breaks” [88, 89] are more likely to be used. Also, planning short time slots between or during long digital meetings to allow employees move leave their desks briefly may also help prevent long sitting bouts. Also, it demonstrates social and leadership support for increasing PA during the workday. Other possibilities are “walk and talk meetings”, which may be promoted with signposted trails around the office and which can be “booked” in the digital calendars [90].

## Conclusions

We found indications that WFH increases ST and decreases the step count on working days compared to onsite working. More post-pandemic research with accelerometric and longitudinal data on SB, including variables describing SB patterns (e.g., mean sedentary bout length and number of STS) and the distinction of PA levels is needed. It is crucial that future research on workers SB and PA when WFH considers homogenous occupational groups (e.g., office workers). It should be taken into account if tasks done at home differ from those conducted onsite to obtain reliable and realistic results. Interventions to promote PA at work will need to be developed or adapted for WFH/hybrid working and evaluated. The chance to be physically active due to the gained time when WFH, should be used to improve health.

## Supplementary Information

Additional file 1: Risk of bias assessment tool (file with used risk of bias tool).

Additional file 2: Data extraction (Table with data extracted from original publications).

Additional file 3: List of excluded studies (Authors and references with reason for exclusion).

## Data Availability

No datasets were generated or analysed during the current study.

## Abbreviations

C: Comparison
CI: Confidence interval
I/E: Intervention/exposure
LCI: Lower confidence interval
MET: Metabolic equivalents of tasks
O: Outcome
OR: Odds ratio
P: Population
PA: Physical activity
PI: Physical inactivity
SB: Sedentary behaviour
SB30: Sedentary bouts longer than 30 minutes length
SB60: Sedentary bouts longer than 60 minutes length
SD: Standard deviation
SE: Standard error
ST: Sedentary time/sitting time
UCI: Upper confidence interval
WAO: Working at the office
WFH: Working from home
WP: Workplace
