# Correction: The impact of working from home on sedentary behaviour and physical activity compared to onsite work in the working population: a systematic review and meta-analysis

**DOI:** 10.1186/s12889-026-26652-6

**Published:** 2026-02-24

**Authors:** Carina Schöne, Martha Sauter, Eva-Maria Backé, Michaela Prigge, Claudia Brendler, Janice Hegewald

**Affiliations:** 1 Unit 3.1 Prevention of Work-Related Diseases, Federal Institute for Occupational Safety and Health, Nöldnerstr. 40-42, Berlin, 10317 Germany; 2 Unit 3.4 Medical Occupational Safety and Health, Occupational Diseases, Federal Institute for Occupational Safety and Health, Nöldnerstr. 40-42, Berlin, 10317 Germany

**Correction: BMC Public Health 25**,** 3963 (2025)**

**https://doi.org/10.1186/s12889-025-24960-x**

Prior to and following publication of the original article [1], the authors reported an error that had been introduced during copy-editing to Table 3. The results contained repetitive decimal points in the rows for Kitano et al. (2024) and Sauter et al. (2025).

The table has since been corrected in the original article.
