# Supplementary material for: The impact of working from home on sedentary behaviour and physical activity compared to onsite work in the working population: a systematic review and meta-analysis
Source: BMC Public Health. 2025 Nov 17;25:3963. doi: 10.1186/s12889-025-24960-x (PMC12621373; doi:10.1186/s12889-025-24960-x)
Supplement: Supplementary file 1 — Additional file 1: Risk of bias assessment tool (file with used risk of bias tool). [file 12889_2025_24960_MOESM1_ESM.pdf]

# Additional file 1: Risk of bias assessment

## General information

Risk of bias tool was developed according to the study question, structure is based on the risk of bias tool described in Bolm-Audorff et al. (2020), criteria have been formulated according to other tools for cross sectional studies (Moola et al. 2017; Downes et al. 2016; National Heart Lung and Blood Institute 2013) and on considerations of the authors (Federal Institute for Occupational Safety and Health, BAuA) on the specific review question.

## Risk of bias tool

| | Risk | Criteria | Comment |
|---|---|---|---|
| **Major risk of bias domains** | | | |
| 1. Study population and recruitment procedure | | | |
| 1.1. Description and suitability of study population | low | ☐ The study subjects and the setting were described in detail or inclusion and exclusion criteria were described (JBI[1])<br>**and**<br>☐ The study sample is suitable for answering our study question (BAuA[2])<br>Note: Employees with sedentary work, who have the possibility to work from home are included | |
| | high | ☐ The study subjects and the setting were not described in detail or inclusion and exclusion criteria were not described (JBI)<br>**or**<br>☐ The study sample is not suitable for answering our study question (BAUA)<br>Note: e.g.: reporting only a mix of paid employees and students is not ideal | |

| | | | |
|---|---|---|---|
| 1.2 Selection process | low | ☐ The selection process (including inclusion/exclusion criteria) was likely to select subjects/participants that were representative of the target/reference population under investigation (panel studies included) (AXIS[3])<br>**and**<br>☐ The participation rate of eligible persons was at least 50% (NIH[4]) or representativeness is demonstrated or per weighting achieved (BAuA)<br>Note: if non-responder analysis was performed indicating that study sample is equal to target population a response rate <50% will be rated as low risk | |
| | high | ☐ The selection process (including inclusion/exclusion criteria) was not likely to select subjects/participants that were representative of the target/reference population under investigation (e.g., a convenience sample will be rated as high risk of bias) (AXIS)<br>**or**<br>☐ The participation rate of eligible persons was lower than 50% (NIH) or response rate or representativeness is not demonstrated (BAuA) | |
| 2. Exposure measurement | | | |
| 2.1. Definition of exposure | low | ☐ The exposure measures (independent variables) were clearly defined and implemented consistently across all study participants (NIH) | |
| | high | ☐ The exposure measures (independent variables) were not clearly defined or not implemented consistently across all study participants (NIH) | |

| | | | |
|---|---|---|---|
| 2.2. Adequacy of comparison group | low | ☐ The comparison group was adequate (BAuA)<br>Note: e.g.: office workers working from home are compared with office workers in the usual workplace at office and there was a relevant difference in time spent in working from home or at the office | |
| | high | ☐ The comparison group was not adequate (BAuA)<br>Note: comparison between office workers and workers not being able to work from home e.g., blue collar workers, will be rated as high risk | |
| 3. Outcome | | | |
| 3.1. Definition of outcome | low | ☐ The subjective outcome measures (dependent variables) were validated (NIH)<br>**or**<br>☐ The measures are objective (e.g., accelerometer) or clear and understandable questionnaires (quantifying time sitting or physical activity) (BAuA)<br>**and**<br>☐ The outcome measures were implemented consistently across all study participants (NIH) | |
| | high | ☐ The subjective outcome measures (dependent variables) were not validated (NIH)<br>**or**<br>☐ The measures are not objective (e.g., accelerometer), not validated or unclear or questionnaires are not quantifying (BAuA)<br>Note: e.g.: “Do you sit more than before?”; if no defined assessment (no question) concerning sitting behaviour or physical activity is reported outcome measurement will be rated as high risk<br>**or** | |

| | | | |
|---|---|---|---|
| | | ☐ The outcome measures were not implemented consistently across all study participants (BAuA) | |
| 3.2. Risk of Recall Bias for the outcome and exposure | low | ☐ No risk of recall bias (BAuA) | |
| | high | ☐ Risk of recall bias (BAuA)<br>Note: e.g.: if the participants were asked about sitting times that were longer than 4 weeks ago, recall bias was rated with high risk | |
| 4. Confounding | | | |
| 4.1. Confounding factors and strategies to deal with confounding factors | low | ☐ Confounding factors are identified or strategies to deal with con-founding factors are stated (JBI), (e.g., within subject comparison or stratification)<br>Note: important confounding factors that should be taken into account are age, sex and occupation type (at least blue collar, or white-collar worker)<br>**or**<br>☐ Not applicable<br>Note: e.g.: within subject design of study | |
| | high | ☐ No major confounding factors are identified and no strategy to deal with confounding was stated (JBI) | |
| 5. Statistical analysis | | | |
| 5.1. Appropriate statistical analysis | low | ☐ Authors used appropriate statistical models to reduce bias (JBI) (e.g., standardization, matching, adjustment in multivariate model, stratification, propensity scoring)<br>**and** | |

| | | | |
|---|---|---|---|
| | | ☐ Appropriate statistical models were considered concerning our question of interest (BAuA) | |
| | high | ☐ Authors did not use adequate statistical models to reduce bias (JBI) (e.g., only raw prevalence is described) | |
| **Minor risk of bias domains** | | | |
| 6. | | | |
| 6.1. Pandemic Bias | low | ☐ No risk of pandemic bias (BAuA)<br>Note: For example, pandemic bias was rated with low if data collection was before the COVID-19 pandemic. | |
| | high | ☐ Risk of pandemic bias<br>Note: if there is a reason to believe (e.g., lockdown measures), that COVID-19 pandemic effects study results, pandemic bias was rated as high risk | |
| 7. Ethics/funding/conflict of interest | | | |
| 7.1. Funding sources or conflicts of interest that may affect the authors' interpretation of the results? | low | ☐ Any funding sources or conflicts of interest that may affect the authors' interpretation of the results are reported (AXIS) | |
| | high | ☐ Any funding sources or conflicts of interest that may affect the authors' interpretation of the results are not reported (AXIS) | |
| 7.2. Ethical approval or consent of participants | low | ☐ Ethical approval or consent of participants was attained (AXIS) | |

| | | | |
|---|---|---|---|
| | | | |
| | high | ☐ Ethical approval or consent of participants was not attained (AXIS) | |

[1] JBI: Joanna Briggs Institute "Checklist for Analytical Cross-Sectional Studies" (Moola et al. 2017)
[2] BAuA: Federal Institute for Occupational Saftey and Health: Criteria was defined by the authors; and considered to be important regarding the study question
[3] AXIS*: Appraisal tool for Cross-Sectional Studies (Downes et al. 2016)
[4] NIH: National Institute of Health "Quality Assessment Tool for Observational Cohort and Cross-Sectional Studies" (NHLBI, 2013)

## References


Bolm-Audorff, Ulrich, Janice Hegewald, Anna Pretzsch, Alice Freiberg, Albert Nienhaus, and Andreas Seidler. 2020. 'Occupational noise and hypertension risk: a systematic review and meta-analysis', *International journal of environmental research and public health*, 17: 6281.

Downes, MJ, ML Brennan, HC Williams, and RS Dean. 2016. "Appraisal tool for Cross-Sectional Studies (AXIS). BMJ Open [Internet]. 2016; 6 (12): 1–7." In.

Moola, S., Z. Munn, C. Fufanaru, E. Aromataris, K. Sears, R. Sfetcu, M. Currie, R. Qureshi, P. Mattis, and P-F. Mu. 2017. 'Systematic reviews of etiology and risk', *Aromataris E. Munn Z (Editors) Joanna Briggs Institute Reviewer's Manual - The Joanna Briggs Institute*.

National Heart Lung and Blood Institute, (NHLBI). 2013. 'Quality Assessment Tool for Observational Cohort and Cross-Sectional Studies '. https://www.nhlbi.nih.gov/health-topics/study-quality-assessment-tools.
