# Supplementary material for: The impact of working from home on sedentary behaviour and physical activity compared to onsite work in the working population: a systematic review and meta-analysis
Source: BMC Public Health. 2025 Nov 17;25:3963. doi: 10.1186/s12889-025-24960-x (PMC12621373; doi:10.1186/s12889-025-24960-x)
Supplement: Supplementary file 2 — Additional file 2: Data extraction (Table with data extracted from original publications). [file 12889_2025_24960_MOESM2_ESM.docx]

Additional file 2: Data extraction

# General Note:

Data was extracted and checked by CP, EB, MS and additionally checked by JH. Data was extracted without decimal places for better clarity. Therefore, it is possible that total percentage deviate from 100% due to rounding error. Wording of the original publication was used.

# Abbreviations:

| ANCOVA | analysis of covariance |
| --- | --- |
| ANOVA | analysis of variance |
| d | Gender divers |
| f | female |
| LPA | light-intensity physical activity |
| h | hours |
| m | male |
| MANOVA | multivariate analysis of variance |
| MET | metabolic equivalent |
| mo | month |
| MPA | moderate physical activity |
| MVPA | moderate to vigorous physical activity |
| n/no/# | number |
| n. a. | not applicable |
| PA | physical activity |
| PB | physical behaviours |
| PI | physical inactivity |
| r | pearson correlation |
| RoB | risk of bias |
| SB | sedentary behaviour |
| VPA | vigorous physical activity |
| WAO | work/working at the office |
| wk | week |
| WFH | work/working from home |
| yrs. | years |

**Table A 1.** Study Characteristics

| **General** | **Study** | **Population** | **Exposure and comparison** | **Outcomes** |
| --- | --- | --- | --- | --- |
| **Abed Alah et al.**  2022  Qatar  no study name  (No underlying cohort)  04/01-28/2/2021 | Study design:  cross-sectional  Measurement:  online anonymous self-administered questionnaire (SurveyMonkey software) which was adopted from other validated and reliable questionnaires  Recruitment method:  convenience sampling (social media platforms of the Hamad Medical Corporation (accessible by the public); snowball sampling (emails and WhatsApp groups) | n=1061 (f: 304 (29%); m: 757 (71%))  Age:  no mean age presented   \| Age in yrs. \| N (%) \| \| --- \| --- \| \| 18-34 \| 429 (40) \| \| 35-54 \| 585 (55) \| \| ≥55 \| 47 (4) \|   Eligibility criteria:  working adults (18+) regardless of type of profession or occupation residing in Qatar during the period of COVID-19 related home confinement measures  Response rate:  n. a. due to sampling | Exposure:  work from home group  n=565 (53%):  82,8% (n=468) do mostly in office work, 17,2% (n=97) do mostly in field work  Comparison:  working regularly group (in their usual workplace)  n=496 (47%):  54,6% (n=271) do mostly office work, 45,4% (n=225) do mostly in field work  Method for measuring exposure and comparison:  question whether participants shifted to work from home or not during home confinement measures | Primary outcome:  perceived changes in physical activity (PA) and sedentary behaviour (SB) since the start of home confinement measures  Definition and assessment PA:  exercise (hours/day) before and during of home confinement measures validated questionnaire (self-reported)  Definition and assessment SB:  sitting/reclining (hours/day) before and during home confinement validated questionnaire (self-reported)  Secondary outcome:  changes in weight, subjective sleep |
| **Barone Gibbs et al.**  2021  USA  **Re**ducing **Se**dentary Behavior on Blood Pressure (RESET BP)  (no underlying cohort)  05-06/2020  *Publication is considered together with Barone Holmes et al. 2023 & Holmes et al. 2025.* | Study design:  longitudinal (as a follow-up of the intervention study RESET BP (NCT03307343))  #waves:  2, before and during COVID shelter at home  Follow-up: during COVID shelter at home  Measurement:  electronic survey  Recruitment method:  RESET BP participants who had completed the trial were recontacted | n=112 (f: 77 (69%); m: 35 (31%))  (n analysed=103)  Age:  mean age: 45.4 yrs.  Eligibility criteria:  desk workers in Pennsylvania with elevated, untreated blood pressure, who self-reported ≥20 h of deskwork and <150 min of moderate-to-vigorous intensity PA per week  Response rate:  RESET BP (intervention study): not reported, 300 have been recruited for baseline^[[1]](#footnote-1)^, 134 completed RESET BP  presented study: 84% (134 invited, 112 baseline and 103 completed) | Exposure:  always remote (defined as ≥50% remote work)  n=15 (14.6%)  Comparison:  never remote (defined as <50% remote work)  n=14 (13.6%)  Other study groups  changed to remote (defined as from <50% to ≥50% remote work)  n=74 (71.8%)  Method for measuring exposure and comparison:  participants were asked to report employment characteristics immediately before and during COVID-19 shelter-at-home to describe changes in work practices (work duration, work-time activity patterns and the social and physical environment of their workplace) | Primary outcome:  difference of the longitudinal changes in SB and PA between the always remote group and the never remote group  Definition and assessment SB:  workday (h/day)  non-workday (h/day)  measured by the validated SB questionnaire  Definition and assessment PA:  moderate activity (min/week),  vigorous activity (min/week),  moderate-to-vigorous activity (min/week);  all measured by the validated Paffenbarger PA questionnaire  Secondary outcome:  sleep, dietary habits, mood disturbance, quality of life, work health |
| **Bérard et al.**  2021^[[2]](#footnote-2)^  France  PSYCOV-CV  (MONALISA)  17/04-10/05/2020 | Study design:  cross-sectional design (population-based cohort)  Measurement:  telephone interviews by trained researchers  Recruitment method:  probability sample recruited from a previous population-based study (MONALISA), where participants were randomly sampled from the French general population | n=536 (f: 279 (52%); m: 257 (48%) based on Bérard, 2021)  Age:  median age: 67 yrs.  Eligibility criteria:  French general population (from south-western France (Toulouse area))  50-89 yrs.  Response rate:  MONALISA^[[3]](#footnote-3)^: 70% in the Lille urban area, 40% in the Bas-Rhin County and 50.2% in the Haute-Garonne County  PSYCOV-CV: 69% (invited: 778, baseline: 536) | Exposure:  job at home during lockdown (e.g., teleworking)  n=74 (13.9%)  Comparison:  job (out of home) without in-person contacts with the public during lockdown (e.g., dustmen)  n=31 (5.8%)  Other study groups:  working with in-person contact with the public (e.g., cashiers, nurses)  n=43 (8.1%)  not working n=386 (72.3%)  Method for measuring exposure and comparison:  no details reported | Primary outcome:  reduced PA since the start of lockdown  Definition and assessment PA:  assessed in minutes/week for sport (e.g., walking, bicycling)  Secondary outcome:  anxiety, depression |
| **Cobbold et al.**  2023  Sydney, Australia  no study name  (Sydney Travel and Health Study (STAHS))  09-11/2019  10-12/2020  10-11/2021 | Study design:  repeat cross-sectional study  #waves: 3  Measurement:  online questionnaires  Recruitment method:  participants were recruited through a commercial consumer panel, with additional participants recruited through email and social media platforms | 2019: n=1937 (f: 1054 (54.4%; m: 878 (45.3%), based on table 1, n=5* not indicated)  Age (2019):   \| Age, years \|  \| \| --- \| --- \| \| 18-24 \| 238 (12.3%) \| \| 25-34 \| 450 (23.2%) \| \| 35-44 \| 465 (24%) \| \| 45-55 \| 438 (22.6%) \| \| >55 \| 346 (17.9%) \|   Eligibility criteria:  needed to be aged 18-70 years old and residing in the greater Sydney area  #invited: unknown  #wave 1 (2019): n=1937  #wave 2 (2020): n=1706  #wave 3 (2021): n=1514  a cohort of 673 individuals from 2019 returned in 2020, 369 from 2019 returned in 2021, 473 from 2020 returned in 2021, while 312 individuals participated in all three waves.  Response rate:  STHAS: n. a. due to sampling  presented study: n. a. due to sampling  *self-calculated | Exposure:  “more” WFH compared to pre-pandemic n=663  Comparison:  “less or same” WFH compared to pre-pandemic  n=1223  Method for measuring exposure and comparison:  in wave 2 (2020) and 3 (2021), participants were asked to record whether they had changed their levels of WFH. Possible answers were “a lot less”, “a little less”, “no change”, “a little more”, “a lot more” or “does not apply” | Primary outcome:  PA  Definition and assessment PA/SB:  PA behaviours were collected using the *Active Australia Survey*, a validated survey instrument which asks participants to record the frequency and duration of PA sessions undertaken in the previous 7 days. Sessions of walking, moderate, and vigorous PA are recorded separately. Moderate and vigorous PA were combined for analysis as moderate–vigorous PA (MVPA).  Secondary outcome:  - |
| **De Oliveira da Silva Scaranni et al.**  2023  Brazil  no study name  (Brazilian Longitudinal Study of Adult Health (ELSA-Brasil))  07/2020-02/2021 | Study design:  cross-sectional study  Measurement:  participants answered questionnaires by cell phone or computer, using an app  Recruitment method:  participants in the second follow-up wave (n=12636), except for those from São Paulo (n=4194), were invited to participate | n=2544 (f: 1297 (51%*); m: 1247 (49%*))  Age:  mean age: 55.2 yrs.  Eligibility criteria:  active working civil servants from teaching and research institutions  Response rate:  ELSA-Brasil:  n. a. due to sampling  (The sample is constituted of volunteers and people who were actively recruited from lists of employees provided by the institutions) ^[[4]](#footnote-4)^  presented study: 67% (invited: 8442; baseline: 5639)  *self-calculated | Exposure:  working from home (WFH)  n=1988 (78.1%*)  Comparison:  no WFH  n=556 (21.9%*)  Method for measuring exposure and comparison:  WFH was assessed with the following question: “Since the beginning of social distancing, have you done work from home?” The options were “no” or “yes”.  *self-calculated | Primary outcome:  SB, leisure time and domestic PA  Definition and assessment SB:  Participants were asked the number of hours they spent  sitting down, reclining or lying down daily on a typical day, the outcome was categorized into “≤ 8h/day” (reference) vs. “> 8h/day” of cumulative sitting time.  Definition and assessment PA:  was measured in two domains: leisure-time and domestic domains with the International PA Questionnaire (IPAQ), long version (frequency and duration of these physical activities performed in minutes/week), based on the leisure-time domain of PA, participants were classified as physically active (reference) and inac­tive (< 150 min/week of moderate PA (MVPA) or < 75 min/week of vigorous PA (VPA) or < 150 min/ week of a combination of the equivalent of both intensities)  Secondary outcome:  - |
| **Delanoeije et al.**  2024  Belgium  No study name  (no underlying cohort)  04-06/2021 and  11-12/2021 | Study design  Repeated cross-sectional  Measurement Periods:  spring (04-06/2021) with six possible waves and autumn with two possible (11-12/2021)  Measurement in each wave:  Daily survey for 10 consecutive workdays  Recruitment methods:  Convenience sample of 457 Belgian employees working from home at least once a week and for at least 1 year | n_total_=360  n_spring_=284 (f:243* (85.6%)  n_autum_=151 (f:125* (82.8%; n=75 of n_spring_)  Age:  Mean age_spring_: 39.04 (SD 19.10)  Mean age_autumn_: 40.60 (SD11.01)  Eligibility criteria:  Work from home at least once a week and working for at least one year  Response rate:  *Spring data:* 387 respondents filled out at least two of the daily surveys (84.7% of those responding at baseline)  Of these respondents, 284 respondents provided full data at T0 and were included in the final sample. Final sample offered 2511 out of 2840 (284* 10 days) possible observations (88.4%).  *Autumn data:* 160 of 176 employees filled out at least 2 daily surveys (response rate: 90.9%). Of these respondents, 151 respondents provided full data at T0 and were included in the final sample. Final sample offered 1298 out of 1510 (151*10 days) possible observations (86.0%).  *self-calculated | Exposure:  day WFH (n_Spring_: 1828*/2511 days, 72.8%; n_autumn_: 905*/1298 days, 69.7%)  Comparison:  Day non-telecommuting (n_Spring_: 417*/2511 days, 16.6%; n_autumn_: 225*/1298 days, 17.3%)  Methods for measuring exposure and comparison:  Respondents indicated in the daily survey that they had worked from home or elsewhere; “…working from home meant executing work tasks that are normally done at the workplace or at home during regular work hours.”  ***self-calculated** | Primary outcome:  PA  Definition and assessment of PA:  Godin Leisure-Time Exercise Questionnaire adapted to the daily level Respondents indicated how many minutes they spent on,  (1) strenuous exercise (e.g., running),  (2) moderate exercise (e.g., fast walking, easy bicycling), and  (3) mild exercise (e.g., yoga, easy walking)”. A total physical activity score was calculated as a composite score of the three categories.  Secondary outcomes:  Loneliness, Job Performance |
| **Elangovan et al.**  2021  India  no study name  (no underlying cohort)  24/06/-03/07/2020 | Study design:  cross-sectional design  Measurement:  online questionnaire (via Research Electronic Data Capture software system)  Recruitment method:  via Whatsapp (as many as possible, no pre-calculated sample size) | n=1023 (f: 483 (47.2%); m: 539 (52.7%); transgender: 1 (0.1%))  Age:  no mean age presented   \| Age in yrs. \| N (%) \| \| --- \| --- \| \| ≤25 \| 124 (12.1) \| \| 26-39 \| 517 (50.5) \| \| 40-59 \| 366 (35.8) \| \| ≥60 \| 16 (1.6) \|   Eligibility criteria:  employees of the Government and private sectors in India (>12+ yrs.)  Response rate:  n. a. due to sampling | Exposure:  WFH  n=383 (37%)  Comparison:  work at office  n=640 (63%)  Method for measuring exposure and comparison:  no details reported | Primary outcome:  changes in PA before and during the lockdown  Definition and assessment PA:  exercise (h per week) in 4 categories ; questions were framed such that the respondents recorded changes in their lifestyle before and during the lockdown  Secondary outcomes:  sleeping habits, food intake habits, perception on weight |
| **Fukushima et al.**  2021  Japan  no study name  (MyVoice Communi-cation database)  28/07-02/08/2020 | Study design:  repeated cross-sectional study design (5^th^ wave of a series of surveys)  Measurement:  online questionnaire  Recruitment method:  via a Japanese internet research company by e-mail (MyVoice Communication) 🡪 fixed participants were followed-up in the 5^th^ survey wave | n=1239 (f: 505 (41%); m: 734 (59%))  Age:  mean age: 44.7 yrs.  Eligibility criteria:  workers living in seven prefectures in the Tokyo Metropolitan Area and working in primary (n=8), secondary (n=251) and tertiary (n=943) industry (self-employed workers were excluded from the analysis)  Response rate:  presented study: not reported  note: MyVoice Communication database: from 1.12 million registered participants (January 2020) 8156 were invited on February 25^[[5]](#footnote-5)^ | Exposure:  WFH (between 1%-100%): n=494 (39.9%)  WFH subgroups by the percentages of WFH:   - 1%-25%: n=112 - 26%-50%: n=105 - 51%-75%: n=48 - 76%-100%: n=229   Comparison:  no WFH (participants who reported 0% work from home): n=745 (60.1%)  Method for measuring exposure and comparison:  participants who had stated their latest job characteristics as full-time jobs, part-time job, and self-employed  participants were asked: “What percentage of your work entails working from home? (0%, if all the work is done at the workplace; 100%, if all the work is done from home)?” | Primary outcomes:  time spent in occupational PA and SB  Definition and assessment PA:  standing (=LPA)  walking (=MPA)  heavy PA (=VPA)  MVPA (calculated by summing up the time spent in MPA and VPA)  SB bout length  all measured using the Work-related PA Questionnaire (WPAQ) measuring proportions of sitting standing and walking and engaging in heavy labour  Definition and assessment SB:  sitting time  measured using the Work-related Physical Activity Questionnaire (WPAQ)  Secondary outcome:  - |
| **Grubben et al.**  2022  The Netherlands  no study name  (Dutch LISS panel)  07/06-27/07/2021 | Study design:  cross-sectional analyses with the data from a longitudinal study (LISS)  Measurement:  Online questionnaires  Recruitment method:  population based weighted random sample from the nationwide Dutch LISS panel | n=1506 (f: 768 (51%); m: 738 (49%))  Age (based on table 1):  no mean age presented   \| Age in yrs. \| N (%) \| \| --- \| --- \| \| 18-34 \| 316* (21) \| \| 35-54 \| 693* (46) \| \| ≥55 \| 497* (33) \|   Eligibility criteria:  workers in paid employment (for at least 8 h/week), working or assisting in a family business, or being an autonomous professional, freelancer or self-employed, aged 18+  Response rate:  Dutch LISS panel^[[6]](#footnote-6)^: about 80% of the eligible persons living in the registered panel households participate in the panel; the monthly response of these participants varies between about 50% and 80%, depending on the questionnaire and month  for presented study: not reported  *self-calculated | Exposure:  WFH (at least 1 h/week)  n=738 (49%)  Comparison:  no WFH (less than 1 h/week)  n=768 (51%)  Method for measuring exposure and comparison:  participants were asked how many hours they spent WFH in a normal week during COVID-19 (April and May 2021) 🡪 based on this information, respondents were categorized who worked from home for at least one hour as WFH and the others as not WFH | Primary outcome:  sports participation  Definition and assessment sports participation:  sports participation in the last 3 months, participants classified in two categories: participating in sport and not participating in sport, subjective interpretation of sports  Secondary outcome:  - |
| **Hallman et al.**  2021  Sweden  no study name  (Flexible Work: Opportunity and Challenge (FLOC) cohort^[[7]](#footnote-7)^)  05-07/2020 | Study design:  cross-sectional (observational field study with within subject design)  Measurement:  collection of questionnaires based and accelerometer data of employees in a municipal division in Sweden  Recruitment method:  invitation of workers from a municipal division in Sweden (Sektor Livsmiljö Gävle), selection of those with interest in accelerometer measurement | n=27 (f: 22 (81%); m: 5 (19%))  Age:  mean age: 43.4 yrs.  Eligibility criteria:  white-collar workers predominantly involved in office-based tasks, working during business hours, and having a permanent full-time employment contract  Response rate:  FLOC: n. a. due to sampling (Purposive convenience sampling of organisations and employees within organisations)  presented study: 17,9% (invited: 484, baseline: 27) | Exposure:  WFH (average: 1.7 days)  n=27 (100%)  Comparison:  working at the Office (WAO) (average: 2.1 days)  n=27 (100%)  within subject comparison  Method for measuring exposure and comparison:  information from company records on work arrangement (flexible time or non-regulated working hours)  question to report if work was done at the office or at home | Outcome:  physical behaviours (PB) during Work: sedentary (sitting/lying), standing, moving (i.e., walking, running, cycling))  PB during leisure time: sedentary, standing, moving  Definition and assessment PB:  time spent sitting/lying (SED), standing and moving (i.e., walking, walking stairs, running, cycling) measured by accelerometer recordings (over 7 days) in min/day during working hours resp. leisure time  classification of time periods (time in bed, working, leisure time) based on diary  Secondary outcome:  sleep |
| **Henke et al.**  2016  USA  no study name  (no underlying cohort)  2010 and 2011 | Study design:  longitudinal study  #waves:  2 (2010 and 2011)  Measurement:  data from Prudential Financial Inc. (employee demographic data, medical claims, WebMD Health Risk Assessment (HRA) data, employee remote connectivity hours) from 2010 and 2011  Recruitment method:  taking data from Prudential Financial Inc. | n=3703 (f: 2296 (62%); m: 1407 (38%))  Age:  no mean age presented  no age distribution presented  about 88% of the sample were younger than 55 yrs.  Eligibility criteria:  active Prudential Financial employees (18-64 yrs.) who had continuous medical enrolment between 2010 and 2011 and completed HRA between 2010 and 2011 with valid nutrition, weight, and exercise values  Response rate:  n. a., due to sampling  note: a typical participation rate for HRA is approximately 78% for Prudential Financial Employees (participation in the HRA is voluntary) | Exposure:  prime time telecommuter (≥51% of the remote hours are during prime work hours (from 6a.m. to 6p.m.))  n=2152 (58.1%)  off-hour telecommuter (≤50% of the remote hours are during prime work hours)  n=747 (20.2%)  Comparison:  non-telecommuter (no remote hours)  n=804 (21.7%)  Method for measuring exposure and comparison:  data from database including employee remote connectivity hours  prime time telecommuters are stratified in 4 intensity levels on the basis of their number of remote connection hours per month:  low intensity (<8 hours): n=696  medium intensity (9-32 hours): n=698  high intensity (33-72 hours): n=420  very high intensity (>73 hours): n=338 | Outcome:  physical inactivity (PI)  Definition and assessment PI:  reporting fewer than 3 days of cardiovascular exercise per week  Secondary outcomes:  obesity, depression, stress, alcohol abuse, poor nutrition, tobacco use, Edington score for overall health risk |
| **Herbolsheimer et al.**  2024  Germany  no study name  (NAKO)  30/04-12/05/2020 | Study design:  cross-sectional study  Measurement:  questionnaire via a web-based survey tool or via mail  Recruitment method:  subsample of the NAKO-sample, participants who consented to be contacted again | n=152,421* (f: 79,411 (52.1%); m: 73010* (47.3%*))  n=104,937 ^a^ (working population)  Age  Mean: 53.7 years  Median: 55 years (IQR: 46-64 years)  Eligibility criteria:  age between 20 and 69 years; proficiency in the German language; residency in one of the 18 study regions.  Response rate:  underlying cohort: NAKO not reported  presented study:77.04%* (#invited: 197,834; #participated: 152,421)  *self-calculated  ^a^ personal communication | Exposure:  switch to remote work  n=42,830* (28.1%)  n=42,184^a^* (40.2%^a^) (working population)  Comparison:  no switch to remote work  n=109,591* (71.9%*)  n= 62,752 ^a^ * (59.8%^a^ *) (working population)  Method for measuring exposure and comparison:  response options, on a multi-choice scale encompassed “switched to remote work”  *self-calculated  ^a^ personal communication | Outcome:  PA and SB  Definition and assessment PA/SB:  changes in PA and SB due to pandemic-related restrictions, inquiring: “How has your physical activity changed due to the coronavirus pandemic in the following domains?”    The following six domains served as separate outcomes: 1) physical activity at work, 2) household activities, 3) recreational activities (e.g., gardening, recreational walking), 4) sports activities (e.g., running, strength training), 5) activities for transport, and 6) sedentary behavior.  The response options included “much less than before” (-2), “a little less than before” (-1), “the same” (0), “a little more than before” (1), and “much more than before” (2).  A subsequent question documented the adherence to the WHO physical activity guidelines (i.e., 150 min of moderate-to-vigorous (MVPA) throughout the week).  Participants stated the number of days they engaged in moderate-to-vigorous physical activity, posing the query, “How many days per week did you usually participate in physical activity for 30 min or more, causing an increased breathing rate?”. This was assessed separately for the time before and during COVID-related restrictions. Individuals were categorized into four groups based on their physical activity trajectories considering both time points: (1) “maintenance category” for those adhering the WHO guidelines at both time points, (2) “non achieving category” for those falling below the recommended PA levels at both time points, (3) “increasing category” for those initially not meeting the recommendations but enhancing activity to reach the guidelines during the restrictions and (4) “decreasing category” for those unable to maintain recommended activity levels.  Secondary outcomes:  - |
| **Holmes et al.**  2023  USA  **Re**ducing **Se**dentary Behavior on Blood Pressure (RESET BP)  no underlying cohort  12/2007-08/2022  *Publication is considered together with Barone Gibbs et al. 2021 & Holmes et al. 2025.* | Study design:  cross-sectional  Measurement:  questionnaire, objective activity monitoring data, diary  Recruitment method:  secondary analysis of baseline data from the RESET BP trial (clinicaltrials.gov identifier: NCT03307343) described elsewhere^[[8]](#footnote-8)^ | n=271 (f: 161 (59.4%); m: 109 (40.2%); other: 1 (0.4%))  Age:  mean age: 45.1 yrs.  Eligibility criteria:  desk workers in Pennsylvania with elevated, untreated blood pressure, who self-reported ≥20 h of occupational sitting time at their desk and <150 min of moderate-to-vigorous intensity PA per week  Response rate:  RESET BP (intervention study): 300 have been recruited for baseline^8^, 134 completed RESET BP | Exposure:  home-based work  n=91 (33.6%*)  Comparison:  office-based work  n=179 (66.4%*)  Method for measuring exposure and comparison:  In-office or home-based work setting was assessed verbally and recorded by the assessment team using the worksite location evaluation  *self-calculated | Outcome:  SB and PA  Definition and assessment: SB and PA:  objectively measured for 7 days at baseline using a thigh-worn activPAL3 micro activity monitor  Objective data were collected during times reported as working, durations of work wear time spent in any SB (total SB), in bouts of SB that were ≥30 min (SB30) and ≥60 min (SB60), standing, stepping, and total monitor wear time, as well as sit-to stand transitions and step counts, were averaged over valid workdays.  Secondary outcome:  - |
| **Holmes et al.**  2025  USA  **Re**ducing **Se**dentary Behavior on Blood Pressure (RESET BP)  no underlying cohort  12/2007-08/2022  *Publication is considered together with Barone Gibbs et al. 2021 & Holmes et al. 2025.* | Study design:  cross-sectional  Measurement:  questionnaire, objective activity monitoring data, diary  Recruitment method:  secondary analysis of baseline data collected from 12/2007-08/2022 for the RESET BP trial described elsewhere^[[9]](#footnote-9)^ | n=275 (f:162 (58.7%), m:112 (40.9%), other:1 (0.04%))  Age:  Mean age: 45.1 (SD:11.6)  Eligibility criteria:  desk workers in Pennsylvania, who self-reported ≥20 h of occupational sitting time at their desk, <150 min of moderate-to-vigorous intensity PA per week, and two clinic BP measurements above normal (either systolic BP between 120–159 mmHg or diastolic BP between 80–99 mmHg). Participants were excluded if they were on glucose-lowering or antihypertensive medication, had diagnosed cardiovascular disease, or were currently or recently pregnant. Participants were also excluded if they were currently using a sit-stand desk and medical clearance from a healthcare provider to participate.  Response rate:  RESET BP (intervention study): 300 have been recruited for baseline^8^, 11 did not indicate worksite as home or office, | Exposure:  WFH  n=93 (33.8%)  Comparison:  Office  n=182 (66.2%)  Methods for measuring exposure and comparison:  We measured primary worksite location by self-report. The participants either answered “Home” or “Office” based on their primary work location. | Primary outcome:  SB, PA  Definition and assessment of SB:  Device-measure SB was captured using the activPAL3 micro (PAL technologies, Glasgow, Scotland), self-reported SB was captured using responses to the validated and reliable SB Work/Non-Workday Questionnaire (SBQ)  Definition and assessment of PA:  Device-measured MVPA was obtained from an ActiGraph GT3X (ActiGraph LLC, Pensacola, FL, USA), self-reported method of measuring MVPA was the validated Paffenbarger PA Questionnaire, LPA as assessed using device-based measures (no self-reported instrument was used)  Secondary outcomes:  Sleep, time watching television |
| **Ishibashi et al.**  2022  Japan  no study name  (no underlying cohort)  03-25/08/2020 | Study design:  repeated cross-sectional study design  Measurement:  online questionnaire and diary (activity time survey)  Recruitment method:  using a subsample of survey data from “Covid-19 lifestyle activity survey” (CLAS) (Unclear how the CLAS-sample was recruited) | n=4484 (f: 1389 (31%); m: 3095 (69%))  Age:  no mean age presented   \| Age in yrs. \| N (%) \| \| --- \| --- \| \| 18-39 \| 1363 (30) \| \| 40-59 \| 2301 (51) \| \| ≥60 \| 820 (18) \|   Eligibility criteria:  second and tertiary industry workers, working >4 hours at all three time points (Weekdays before the COVID-19 pandemic (Before), weekdays during the first declared state of emergency of April 16-May 13 2020 (During) and on Thursday July 30, 2020 (After); 18+years  Response rate:  not reported | respondents divided into groups according to the workstyle (telework or commuting to work) at each time point (Before/During/After COVID19 pandemic, workers were defined as teleworkers, if they were working “at home” for >4h per day  Exposure:  C/C/C continued commuting during the COVID-19 pandemic  n=614 (13.7%)  Comparison:  T/T/T were teleworking before, during and after the COVID-19 pandemic  n=2859 (63.8%)  Other study groups:  C/T/C teleworked only in the “During” period  n=585 (13.0%)  C/T/T started teleworking in the “During” period and did not revert to commuting  n=426 (9.5%)  Method for measuring exposure and comparison:  in the activity time survey, respondents reported “where they were” | Outcome:  PA  Definition and assessment PA:  metabolic equivalent (MET) (based on information on activity and mode of transport) was used to assess the amount of PA related to daily trips (commuting trips and total daily trips)  Secondary outcome:  - |
| **Kikuchi et al.**  2025  Japan  No study name  (no underlying cohort)  05/2023 | Study design:  Observational study  Measurement:  Questionnaires, activity diary and accelerometric measurement  Recruitment method:  Snowball sampling method | N=177 (f:73 (41.2%), m:104 (58.8%))  Age:  Mean age: 34.7 (SD: 9.7)  Eligibility criteria:  Not reported for study participation, exclusion criteria for analysis were insufficient wear time for the accelerometer and working only at the office or at home  Response rate:  n. a. due to sampling | Exposure:  days working from home of 177 participants working at least 2 days from home  Comparison:  days working at the office of 177 participants (within comparison) with at least 2 days at the office  Method for measuring exposure and comparison:  Participants were asked to record their working status in their diaries for eight day. In the diary, they chose daily status from five options, i.e., “WFH only”, “WAO only”, 7  Both WAO and WFH”, “holidays” and “others” | Primary outcome:  SB, PA  Definition and assessment SB:  Time with SB (<1.5 MET) in minutes/day measured via validated accelerometer (active style Pro HJA-750C, Omron Healthcare, Kyoto Japan)  Definition and assessment PA: Count of steps, light PA (1.5-2.9 MET), moderate PA (3.0-5.9 MET), vigorous PA (≥6.0 MET) in minutes/day measured via validate accelerometer (active style Pro HJA-750C, Omron Healthcare, Kyoto Japan)  Secondary outcomes:  - |
| **Kim et al.**  2022  Japan  no study name  (no underlying cohort)  01-03/2021 | Study design:  cross-sectional analysis using data from an intervention study  Measurement:  self-administered questionnaires conducted via an online survey;  triaxial accelerometer  Recruitment method:  via an internal newsletter in an insurance company located in Tokyo or branches in Osaka, Nagoya, Fukuoka, and Sapporo | n=64 (f: 10 (16%); m: 54* (84%))  Age:  mean age: 49.3 yrs.  Eligibility criteria:  Japanese office workers; 20-65yrs  Response rate:  23.4%*: (invited: 274; baseline: 64)  *self-calculated | Exposure:  remote work  Comparison:  office-based work  Method for measuring exposure and comparison:  participants were instructed to keep a daily diary to note among others their type of work (remote or commuting) | Outcome:  PA  Sedentary Time (ST)  Definition and assessment PA:  time in min with moderate-to-vigorous PA (MVPA), light PA (LPA), moderate PA (MPA),  vigorous PA (VPA), steps, assessed by wearing a triaxial accelerometer for 7 consecutive days  Definition and assessment ST:  sitting time assessed by wearing a triaxial accelerometer for 7 consecutive days  Secondary outcome:  - |
| **Kitano et al.**  2024  Japan  No study name  (Meiji Yasuda LifeStyle Study)  04/2022-03/2023 | Study design:  Cross-sectional  Measurement:  Questionnaire and accelerometric measurement  Recruitment method:  Since 2017, this health checkup center has voluntarily offered invitations to selected health insurance associations listed in their client databases (ie, some annual examinees) for optional examinations using an accelerometer to assess daily PA and SB. Accelerometers were subsequently mailed to the examinees that agreed to the measurement. The researchers were not involved in the participant selection for the measurement | N=1133 (f:479 (42.3%); m:654 (57.7%))  Age:  Mean age: 42.5 yrs. (SD: 11.8)  Eligibility criteria:  Paid white-collar workers; completion of an accelerometer test at an annual health checkup; content to participate in the study  Response rate:  84.7%* (1408 individuals out of 1662 individuals, who voluntary took part in the accelerometer examination, consented to participate in the study)  *self-calculated | Exposure:  ≥5 days WFH (n=265)  3-4 days WFH (n=179)  1-2 days WFH (n=186)  Comparison:  Never WFH (n=437)  Method for measuring exposure and comparison  Participants were queried, “In the past month, how many days per week did you work from home?” They responded with continuous values ranging from 0 to 7 days. | Primary outcome:  SB, PA  Definition and assessment SB:  Time with SB (≤1.5 MET) in minutes/day measured via triaxial accelerometer (active style Pro HJA750-C, Omron healthcare) which was worn on their hip; prolonged SB (lasting 30 minutes or more consecutives minutes) (minutes/day), prolonged SB counts (times/day), prolonged SB bout duration (min/times)  Definition and assessment PA:  Time in light-intensity PA (LPA: 1.6-2.9 METs) and moderate- to vigorous-intensity PA (≥3.0 METs) in minutes/day measured via triaxial accelerometer (active style Pro HJA750-C, Omron healthcare)  Secondary outcomes:  - |
| **Koohsari et al.**  2021  Japan  no study name  (no underlying cohort)  22/02/2019 and 08/07/2020 | Study design:  survey (baseline and follow up)  #waves:  2  Measurement:  online questionnaire  Recruitment method:  via invitation e-mail with a link to participate in the survey was sent to 45,659 workers (registered individuals of a Japanese internet research service company) | n=2466 (f:1212 (49.1%); m:1254 (50.9%))  Age at baseline:  39.6 (SD: 10.7)  Eligibility criteria:  worker aged 20 to 59 years  Response rate:  7.0% (#invited: 45,659 #responded: 3,200) | Exposure:  absolut changes in days working from home in 2019 before compared to after the outbreak of COVID-19 in 2020  Comparison:  -  Method for measuring exposure and comparison:  for working days, participants also reported the total number of days they worked at their place of employment or worked from home | Outcomes:  PA/SB  Definition and assessment PA:  Global Physical Activity Questionnaire (GPAQ) was used to measure domain specific physical activities.  Definition and assessment SB:  Japanese 6-item questionnaire with a 1-week recall period was used to evaluate domain -specific sedentary behaviours  Secondary outcome:  - |
| **Koyama et al.**  2021  Japan  no study name  (Japan COVID-19 and Society Internet Survey (JACSIS))  08/04-25/05/2020 | Study design:  cross-sectional  Measurement:  web-based self-reported questionnaires  Recruitment method:  simple random sample of panel data (Internet research agency Rakuten Insight Inc.)  Note: The respondents of the Internet survey were weighted to be closer to a nationally representative sample of Japan | n=11623 (f: 4228 (36%); m: 7395 (64%))  Age:  no mean age presented   \| Age in yrs. \| N (%) \| \| --- \| --- \| \| 15-29 \| 2034 (18) \| \| 30-39 \| 2197 (19) \| \| 40-49 \| 2941 (25) \| \| 50-59 \| 2313 (20) \| \| 60-69 \| 1406 (12) \| \| 70-79 \| 732 (6) \|   Eligibility criteria:  workers (no students, not unemployed, more than 0 hours working time),  Response rate:  underlying cohort: JACSIS not reported  presented study: 12,5% (invited panellists: 224389; baseline: 28000), the survey was closed when the target numbers of respondents for each sex, age, and prefecture category were met | Exposure:  starting to work from home during the state of emergency during COVID-19 pandemic  n=1627 (14.0%)  Comparison:  not starting to work from home during the state of emergency during COVID-19 pandemic  n=9996 (86.0%)  Method for measuring exposure and comparison:  question: starting to work from home during the state of emergency: yes/no | Outcome:  SB  Definition and assessment time spent sedentary:  recall of the average daily time (h/day) spent sedentary in April to May 2020, during the first state of emergency, and in June to the time of the survey in 2020 (after the state of emergency)  self-reported times spent sedentary per day were categorized into the following original 11 categories (assigned average hours per day): none (0), <0.5 (0.25), 0.5 (0.5), 1 (1.0), 2 (2.0), 3 (3.0), 4-5 (4.5), 6-7 (6.5), 8-9 (8.5), 10-11 (10.5), and ≥12 (12) hours per day  Definition and assessment of prolonged sedentary time:  calculated by subtracting the sedentary time after the state of emergency (defined as normal) from the sedentary time during the state of emergency  dichotomized variable (1=≥2 hours of prolonged sedentary time and 0=<2 hours of prolonged sedentary time)  Secondary outcome:  - |
| **Leskinen et al**  2025  Finland  No study name  (Special Turku Coronary Risk Factor Intervention Project (STRIP))  05/2021-05/2022 | Study design:  Cross-sectional (nested to the follow-up of the STRIP cohort)  Measurement:  accelerometric measurements, log-data, web-based questionnaires  Recruitment method:  Overall, 396 individuals,  who successfully wore the accelerometers during the STRIP follow-up at the age of 26 were invited to a 1 week-long accelerometer measurement again at the age of 31 years | n=133 (f: 81 (61%), m:52 (39%)*)  Age:  31 (SD:0)  Eligibility criteria:  who successfully wore the accelerometers during the STRIP follow-up at the age of 26 years; who reported to work either full-time or part-time; had reported their current job title; had at least one valid workday measurement day  *self-calculated  Response rate:  56% of invited agreed to participate (223 of 396 individuals) | Interindividual exposure:  Remote worker  n=17 (12.7%)  Hybrid workers  n=35 (26.3%)  Interindividual comparison:  In-office worker  n=49 (36.8%)  Manual workers  n=32 (24.1%)  Intraindividual exposure:  days working from home of the hybrid workers (n=35)  Intraindividual exposure:  Days working at home of hybrid workers (n=35)  Method for measuring exposure and comparison:  […] participants’ logs in which they recorded information about […] whether or not they had worked remotely on that particular day.  Four groups were created according to the participations’ occupational status and their log data of workdays’ modes during the measurement week | Primary outcome:  SB, PA  Definition and assessment of SB:  Measured sedentary time in minutes via triaxial ActiGraph wActiSleep-BT accelerometer (Actigraph, Pensacola, Florida, USA) on their non-dominantwrist for occupational and non-occupational time, sum of time spent in long sedentary bouts (>30 min) for working hours of each workday, sedentary fragmentation index (transitioning from sedentary to active behaviour  Definition and assessment of PA:  Measure active time in minutes via via triaxial ActiGraph wActiSleep-BT accelerometer (Actigraph, Pensacola, Florida, USA) for occupational and non-occupational time, active fragmentation index (transitioning from active to sedentary behaviour)  Secondary outcomes:  - |
| **Loef et al. (1)**  2022a  The Netherlands  no study name  (Lifelines population cohort/ Lifelines COVID-19 cohort)  03/2020-02/2021 | Study design:  longitudinal study (prospective population-based cohort study)  17 questionnaire rounds; one per month between (March 2020-February 2021)  Measurement:  questionnaires  Recruitment method:  invitation of all active participants (≥18 years) of Lifelines population cohort to Lifelines COVID-19 cohort, Life lines cohort was originally recruited via the General Practitioners | n=33325 (f: 19693* (59%*); m: 13632* (41%*))  note: participants with information on physical activity, of these, 18 379 participants with information on SB were included for the analysis with SB as outcome measure; not all 33 325 workers participated in every questionnaire round, percentages are based on the following numbers of included workers per round:  01: n=24,702; 02: n=24,060;  03: n=21,644; 04: n=21,995;  05: n=20,563; 06: n=18,940;  07: n=18,003; 08: n=15,782;  09: n=14,996; 10: n=13,203;  11: n=1,375; 12: n=15,108;  13: n=15,171; 14: n=14,447; 15: n=14,452; 16: n=13,297; 17: n=13,939  Age:  mean age location workers: 50.4 yrs.  Mean age home workers:48.5 yrs.  Mean age hybrid workers: 48.8 yrs.  Eligibility criteria:  Dutch workers (if they worked >75% of the rounds in which they participated in the Lifelines population cohort, and if they, worked >75% of the time on location and/or from home in the rounds in which they worked);  18-67 yrs.  Response rate:  underlying cohort: Lifeline Cohort: not reported  presented study: 55% completed at least one questionnaire round (invited: 140145; baseline: 76421)  presented study: not reported  *self-calculated | Exposure:  home workers  (those who indicated to work from home)  n=8473 (25.4%)  hybrid workers  (those who indicated to work at location as well as from home)  n=8809 (26.4%)  Comparison:  location workers  (those who indicated to work at the usual location and/or at multiple sites for their job)  n=16043 (48.1%)  Method for measuring exposure and comparison:  participants were asked what they currently did in their daily life (student; work; on disability; unemployed; retired; maternity leave; other) 🡪 those who answered “I work” were asked to indicate their current work situation from one or more of the following responses: I work from home; I am laid off but am still being paid; I am laid off and am no longer being paid; I continue to work at the usual location (e.g., office, factory, construction site); I continue to work at multiple sites for my job; I am forced to take sick leave or vacation time; other | Outcome:  PA/SB  Definition and assessment PA:  Participants were asked questions about duration of moderate- and vigorous-intensity PA based on the Dutch PA Guidelines 2017: “how many minutes of (moderately) intense activity did you do (e.g., walking, biking or running) in the last 7 days (rounds 1–6)/ 14 days (rounds 7-17)?”. In rounds 1 and 2, they were also asked: “how many minutes of (moderately) intense activity they performed each week before the COVID-19 pandemic?”.  Responses could be one of five categories (<50 min; 50–100 min; 100–150 min; 150–180 min; >180 min in the last 7 days (rounds 1-6) or <100 min; 100–200 min; 200–300 min; 300–360 min; >360 min in the last 14 days (round 7-17)).  Definition and assessment SB:  in round 6, participants (n=18940) were asked: “How much time they spent sitting on average per day in the past 7 days and before the COVID-19 pandemic. (Answer categories: <1h; 1; 2h; 3h; 4h; 5h; 6h; 7h; 8h; 9h; 10h; 11h; 12h; >12 h) This question was based on the International PA Questionnaire Short Form. Questions  were asked for work- and weekend days separately. In rounds 11 and 14–17, they were asked about time spent sitting per day in the past 14 days  Secondary outcome:  - |
| **Loef et al. (2)**  2022b  The Netherlands  no study name  (Lifelines population cohort/ Lifelines COVID-19 cohort)  03/2020-01/2022 | Study design:  longitudinal study  #waves:  24 questionnaire rounds  March 2020-January 2022  Measurement:  Questionnaires were sent out on a (bi)weekly basis until July 2020, and on a monthly basis from July 2020 onwards.    Recruitment method:  all adult participants from the Lifelines population cohort with known email addresses were asked to participate in the Lifelines COVID-19 cohort (n=140145) | n=28586 (f: not reported; m: not reported)  Age:  mean age home workers: 50.9 yrs.  Mean age hybrid workers: 49.3 yrs.  Mean age location workers: 49.1 yrs.  Eligibility criteria:  Dutch workers (if they worked >75% of the rounds in which they participated in the Lifelines population cohort, and if they, worked >75% of the time on location and/or from home in the rounds in which they worked);  18-67 yrs.  Available information on work situation, PA and musculoskeletal pain  Response rate:  underlying cohort: not reported  presented study:  response rates of the different rounds varied between 28 and 49% | Exposure:  home workers (those who indicated to work from home)  n=5971  hybrid workers (those who indicated to work at location as well as from home)  n=9126  Comparison:  location workers (those who indicated to work at the usual location)  n=13489  Method for measuring exposure and comparison:  Participants were asked what they currently did in their daily life (student; work; on disability; unemployed; retired; maternity leave; other) 🡪 those who answered “I work” were asked to indicate their current work situation from one or more of the following responses: I work from home; I am laid off but am still being paid; I am laid off and am no longer being paid; I continue to work at the usual location (e.g., office, factory, construction site); I continue to work at multiple sites for my job; I am forced to take sick leave or vacation time; other. | Outcome:  PA/SB  Definition and assessment PA:  Participants were asked “how many minutes of (moderately) intense activity did you do (e.g., walking, biking or running)” in the last 7 days (rounds 1–6) or 14 days (round 7 onwards). Answer categories were <50; 50–100; 100–150; 150–180; >180min in the last 7 days or <100; 100–200; 200–300; 300–360; >360min in the last 14 days.  Answers were dichotomized into being physical active less or more than 150min/week  Definition and assessment SB:  participants were asked how much time they on average spent sitting per work day in the last 7 days (round 6) or 14 days (round 11 onwards). Answer categories were <1; 1; 2; 3; 4; 5; 6; 7; 8; 9; 10; 11; 12; >12 h (until round 17) or <1; 1–3; 4–6; 7–9; 10–12; >12 h (from round 19 onwards). Answers were dichotomized into sitting >9 vs. ≤9 h per work day based on the average sitting time of the Dutch population  Secondary outcome:  musculoskeletal pain |
| **Marenus et al.**  2025  USA  No study name  (no underlying cohort)  Not reported | Study design  Cross sectional  Measurement:  Qualtrics questionnaires  Recruitment methods:  Participants were recruited via Prolific using screening filters for current employment status and work setting (onsite, hybrid, remote). A balancing filter was used to achieve an approximately even gender distribution. No additional quotas for race, ethnicity, or income were applied. As such, the sample represents a convenience sample rather than a fully representative cross-section of the U.S. workforce. | n=520 (f:253* (48.6%), m: 258* (59.6%*), d:9* (1.7%*)), based on table 1  Age:  39 years (SD: 11 years)  Eligibility criteria:  Not indicated  Response rate:  Not indicated  *self-calculated | Exposure:  Remote  n=122 (23%)  Hybrid  n=160 (31%)  Comparison:  Onsite  n=238 (46%)  Methods for measuring exposure and comparison:  Self-reported workplace status (onsite, hybrid, remote) | Primary outcome:  PA  Definition and assessment of PA:  Physical Activity (PA). The International Physical Activity Questionnaire (IPAQ)-ShortForm is a 7-item measure to assess the amount of time spent at various levels of PA intensity. Participants were asked to recall the days, hours, and minutes spent on vigorous PA (VPA), moderate PA (MPA), and walking in the last week.PA scores are indicated by the MET-minutes per week. The IPAQ scoring guidelines classify individuals as "highly" active if they engage in at least 1500 MET-minutes/week of vigorous PA or 3000 MET-minutes/week of TPA. Those who engaged in less than 3000 MET-minutes/week of TPA, but more than 600 MET-minutes/week of total PA are considered "moderately" active. Individuals below 600 MET-minutes/week of total PA are classified as "low" active.  Secondary outcomes:  Workplace culture of health, psychological wellbeing, work productivity |
| **Massar et al.**  2022  Singapore  no study name  (Health Insights Singapore (hiSG))  08/2021-01/2022 | Study design:  longitudinal study as part of an intervention study  #waves:  3 study phases  Measurement:  measured PA via consumer fitness tracker (Fitbit Versa 2, Fitbit Inc., San Francisco, CA, US) and […] logs of their work arrangements (WFH, WFO, no work) via an ecological momentary assessment (EMA) phone application.  Recruitment method:  hiSG-Study: Registered volunteers are selected to gather a sample group of participants with a good mix of demographics and lifestyles, as well as health attitudes and perceptions to reflect various segments of the population to best benefit the nation (source: <https://hpb>.gov.sg/healthy-living/hisg, not reported in the article) | n=213 (f: 79* (37.1%*); m:134 (62.9%*))  Age  Mean: 35.5±4.4 years  Eligibility criteria:  Singapore citizens and Permanent Residents aged 18+;  a Singapore-registered (+65) mobile number and personal smart phone (iOS 15 or Android 9 and above) (source: <https://hpb>.gov.sg/healthy-living/higs, not reported in the article)  Response rate:  underlying cohort: not reported  presented study: not reported  #recruited: 225  #included in the baseline characteristics: 213  #included phase 1: 207  #included phase 2: 179  #included phase 3: 174  *self-calculated | Exposure:  Days working from home (WFH)  Phase 1: n= 815*  Phase 2: n=653*  Phase 3: n=518*  Comparison:  Days working in the office and other locations (WFO)  Phase 1: n=598*  Phase 2: n=344*  Phase 3: n=478*  Days with no work  Phase 1: n=600*  Phase 2: n=675*  Phase 3: n=538*  Method for measuring exposure and comparison:  Evening EMA with the question “Where did you work today?” Possible answers were “home”, “office”, “other”, “did not work today”. Work from office/other location were further combined into a single category (WFO) for analysis purposes.  *self-calculated | Outcomes:  PA  Definition and assessment PA:  Daily step count was used as an indicator of physical activity and measured by a consumer fitness tracker (Fitbit Versa 2, Fitbit Inc., San Francisco, CA, US)  Secondary outcome:  sleep |
| **Matthews et al.**  2022  USA  no study name  (AmeriSpeak)  16/10-11/11/2019 and 03/11/-15/11/2020 | Study design:  longitudinal panel survey  (Note: designed as longitudinal study, but due to results for the review research question as cross-sectional design labelled and only cross-sectional data were used)  #waves:  2  Follow Up:2019, 2020  Measurement:  online questionnaire  Recruitment method:  general sample of the AmeriSpeak (probability-based panel, designed to represent the US Population) were invited  study-specific weights were calculated and also adjusted to external population | n=1635 (f: 722 (44.2%*); m: 913 (55.8%*))  Age:  no mean age presented   \| Age in yrs. \| N (%) \| \| --- \| --- \| \| 20-39 \| 654 (39) \| \| 40-64 \| 758 (45) \| \| 65-76 \| 232 (16) \|   Eligibility criteria:  20-75 years of age in 2019, who could complete online surveys  Response rate:  underlying cohort: AmeriSpeak: not reported  presented study:  baseline: 19% (invited: 15153; baseline: 2877)  Follow-up: 69% (invited: 2574 (participants from 2019, who were still in AmeriSpeak panel); follow-up: 1788)  *self-calculated | Exposure:  Working from home  n=400 (20.8%)  Comparison:  Not working from home  n=697 (41.0%)  Method for measuring exposure and comparison:  Question on work from home status with the answer categories “yes, all or some of the time” or “no, not working from home” | Outcome:  PA/SB  Definition and assessment PA/SB:  participants reported PA by selecting from 170 predefined activities organized in 14 major categories using the self-administered ACT24 previous-day recall, to simplify scoring only their primary activities should be reported);  PA behaviours included: light, moderate and vigorous intensity activities.  Follow-up questions assessed the duration of the activity, body position, and other details  SB was defined as sitting/reclining with little energy expenditure while not in-bed/sleeping.  Secondary outcome:  - |
| **Moura et al.**  2022  Brazil  COVID Inconfidentes  (no underlying cohort)  10-12/2020 | Study design:  cross-sectional study (population-based epidemiological household survey)  Measurement:  face to face interviews using a structured questionnaire in an electronic format  Recruitment method:  conglomerate sampling in three stages: census sector (selected with probability proportional to the number of households), household (selected from a systematic sampling), and resident (randomly selected through the application Sorteador de Nomes®)  the sample weight of each selected unit (census sector, household, and individual) was calculated to correlate with the 2019 population projections (DATASUS) | n=1750 (f: 917* (52.4%); m: 833* (47.6%))  Age:  no mean age presented   \| Age in yrs. \| N (%) \| \| --- \| --- \| \| 18–34 \| 614* (35.1) \| \| 35–59 \| 802* (45.8) \| \| ≥ 60 \| 334* (19.1) \|   Eligibility criteria:  adults (≥18 years) with permanent residence in the urban areas of Ouro Preto and Mariana, cognitive ability and venous access for serological testing.  Response rate:  not reported  *self-calculated | Exposure:  those who were working at the time of data collection partially or completely from home were classified as “work from home”  n= 353* (20.2%)  Comparison:  those, who perform all work activities in the work environment were classified as “no work from home”.  N= 560* (32.0%)  n= 837* (47,8%) did not work  *total numbers self-calculated  Method for measuring exposure and comparison:  question on work routine at the time of data collection | Outcome:  leisure time physical inactivity measured before the pandemic (March 2020), and during march to August 03 to 07/2020 and during October to December 10-12/2020)  Definition and assessment:  Individuals who self-reported participating in PA during leisure time were classified as physically active, and those who said they did not participate in PA during leisure time were classified as physically inactive.  Secondary outcome:  - |
| **Moura et al.**  2023  Brazil  COVID Inconfidentes  (no underlying cohort)  10-12/2020 | Study design:  cross-sectional study (population-based epidemiological household survey)  Measurement:  face to face interviews using a structured questionnaire in an electronic format  Recruitment method:  conglomerate sampling in three stages: census sector (selected with probability proportional to the number of households), household (selected from a systematic sampling), and resident (randomly selected through the application Sorteador de Nomes®).  The sample weight of each selected unit (census sector, household, and individual) was calculated to correlate with the 2019 population projections (DATASUS) | n=1750 (f: 917* (52.4%); m: 833* (47.6%))  Age:  no mean age presented   \| Age in yrs. \| N (%) \| \| --- \| --- \| \| 18–34 \| 614* (35.1) \| \| 35–59 \| 802* (45.8) \| \| ≥ 60 \| 334* (19.1) \|   Response rate:  not reported  *self-calculated | Exposure:  full WFH:  n=1397* (79.8%, 95% CI 75.2-83.7)  partial WFH:  n=158* (9.0%, 95% CI 7.1-12.2)  Comparison:  not WFH  n= 196* (11.2%, 95% CI 8.7-14.4)  Method for measuring exposure and comparison:  Participants were asked: “Currently, how is your work routine regarding location?” Possible answers were: (1) all work activities are being performed at my home; (2) part of the activities are performed in the traditional work environment, that is some days at home and other at the workplace”; (3) all work activities are being performed in my work environment.  *self-calculated | Outcome:  leisure-Time PA  Definition and assessment:  PA practice was assessed by self-report. The weekly frequency (0-7 days) was multiplied by the duration (in minutes per day) to quantify the weekly volume of PA of the individuals. Next, volunteers were categorized according to the guidelines established by the WHO and the Physical Activity Guide for the Brazilian Population, being classified as physically inactive (<150 min/wk of moderate PA, <75 min/wk of vigorous activity or who did not achieve an equivalent combination of both intensities) and physically active (≥150 min/wk of moderate PA, ≥75 min/wk of vigorous activity or who did not achieve an equivalent combination of both intensities), based on the compendium indicating intensity of PA  Secondary outcome:  - |
| **Olsen et al.**  2018  Australia  no study name  (no underlying cohort)  time of data collection is not reported | Study design:  intervention study,  single group pre-post design  Measurement:  online survey four weeks before and six weeks after policy implementation  Recruitment method:  information session was held at the workplace, all staff received an e-mail, volunteers from a single business unit | n=24 (f: 15 (62%); m: 9 (38%))  Age:  mean age: 40 yrs.  Eligibility criteria:  volunteers from a single business unit at a financial services organization  Response rate:  46% of the invited participants completed assessments pre- and post-policy implementation (invited: 50; baseline: 24) | Exposure/Intervention:  introduction of flexible work policy: employees were mandated to work at least one day per week at home (pre-post-comparison)  Comparison:  working without flexible work policy (Pre: 4 weeks before implementation)  Additional analysis:  Exposure  behaviour after introduction of intervention on a usual day in homeoffice  Comparison  behaviour after introduction of intervention on a usual workday at the office  n=24 (within subject comparison)  Method for measuring exposure and comparison:  self-reported by the participant | Outcome:  PA/SB  Definition and assessment PA:  self-reported time spent in PA  as the sum of time (min/week) spent in moderate, walking  (Both transport and exercise/recreation) and vigorous activity, using items from the Active Australia survey (adapted version of the questionnaire): Reporting of the total number of sessions (frequency) and total time (duration) spent: walking for recreation or exercise for at least 10 minutes at a time; walking to get to or from places for at least 10 minutes at a time; in vigorous PA (e.g., jogging, cycling, aerobics); in other moderate intensity activities over the past week  Definition and assessment SB:  self-reported sitting time on days working at home or at the usual workplace;  overall sitting time was determined as the sum of daily time spent in five domains (work, travel, television, computer use for leisure, and other leisure)  Secondary outcome:  - |
| **Oxenham et al.**  2025  United Kingdom  No study name  (UK Household Longitudinal Study (UKHLS)  2015-2017 (wave 7) -  2022-2023 (wave 13) | Study design  Longitudinal  Measurement:  Questionnaires  Recruitment methods:  Multi-stage sampling in each wave of the UKHLS^[[10]](#footnote-10)^ | n=3302 (f: 1890 (57.2%), m:1412 (42.8))  Age:  mean age: 21.5 (SD 3.3)  Eligibility criteria:  We included in these analyses all UKHLS participants who started work between the ages of 16 and 30, and responded at least once before and once after starting work to the health behaviour questions in waves 7–13. We did not exclude participants based on health status, or family status, to allow our findings to represent the  entire young adult population.  Response rate:  Not indicated for the waves of UKHLS | Exposure:  work from home  n=128* (3.9%)  Comparison:  Office work  n=2663 (80.6%)  Methods for measuring exposure and comparison:  Self-reported “work location” at first wave of data collection with possible answers “At home”, “At your employer’s premises” (office work) or “Driving or travelling around, At one or more other places, or Other” (not considered within the scope of this review) | Primary outcome:  PA  Definition and assessment of PA:  Three variables from the validated international Physical Activity Questionnaire were used to assess participants’ weekly physical activity habits […]: moderate, vigorous and walking activity. The responses were combined using the formula provided by the IPAQ guidelines to generate a weekly MET-minutes/week.  Secondary outcomes:  diet, sleep |
| **Prince et al.**  2024  Canada  No study name  (Canadian Community Health Survey (CCHS))  06/2021-02/2022 | Study design  Cross-sectional  Measurement:  Self-reported survey  Recruitment methods:  Not indicated, using data of a single wave of the annual CCHS (nationally representative sample of household-dwelling Canadians aged 12+ years living in the 10 province and three territories) | n=10913 (f:5426 (49.7%*), m:5471 (50.1%*), d:16 (0.2%*), based on table 1  Age:  No mean age presented   \| 18-34 \| 2561 (23.5%*) \| \| --- \| --- \| \| 35-49 \| 3794 (34.8%) \| \| 50-64 \| 3539 (32.4%) \| \| 65-75 \| 1019 (9.3%) \|   Eligibility criteria:  Age between 18-75 years, reported working at a job or business in the last week and with complete data on covariates and movement behaviours  Response rate:  Not indicated for CCHS  *self-calculated | Exposure:  Telework  n= 3449* (31.6% [95% CI: 30.0-33.1])  Comparison:  Fixed workplace  n=6188* (56.7% [95%CI: 55.0-58.3]  Other groups:  Non-fixed workplace (n=1288* (11.8% [CI 95%: 10.8-12.7])  Methods for measuring exposure and comparison:  Respondents were asked: “In the past 30 days, in which of these locations did you work the most hours?” Response options included “At a fixed location outside the home (e.g., office building, factory)”, “Outside the home with no fixed location (e.g., driving, makingsales calls)”, “At home (e.g., main residence, cottage)” and ‘Absent from work’. From this, a three-level exposure variable was created, classifying the first three response options as: (1) fixed workplace; (2) non-fixe workplace, and (3) telework, respectively. Those who responded as “absent from work” were excluded.  *self-calculated | Primary outcome:  PA  Definition and assessment of PA:  Respondents were asked about the number of minutes they completed in the previous 7 days for a minimum of 10 continuous minutes in different types of moderate-to-vigorous intensity physical activity (MVPA). Self-reported average minutes per week spent in total and domain-specific MVPA was estimated for transportation, recreational, and occupational/household PA. The proportion who reported no PA was also estimated. The sum of MVPA from all domains totaling =150 min/week was used to assess adherence to the PA recommendation from the 24-H Guidelines and the sum of MVPA from recreation and transportation domains totaling =150 min/week was used to assess adherence to the PA =recommendation based on AT+REC only.  Secondary outcomes:  Screen time, sleep duration, adherence to the Canadian 24h Movement Guidelines |
| **Sauter et al.**  2025  Germany  SITFLEX-1  (no underlying cohort)  07-11/2021 | Study design:  Cross-sectional  Measurement:  Questionnaires, daily diaries and accelerometric measurement  Recruitment method:  The company was recruited by approaching and gaining the support of managers, staff council members, occupational safety and health services and other representatives. Then, all employees at the location (N=6451) were informed about the study via emails and online information sessions. The on-site medical officer invited employees to participate. | n=102 (f: 27 (26.5%), m: 75* (73,5%*))  Age:  Mean age: 38.9 yrs. (95%CI: 37.0-40.7)  Range: 20-60 yrs.  Eligibility criteria:  inclusion criteria: desk-based work, age >18 years, ability to stand and walk, planned to work ≥ 2 days at home and ≥ 2 days at the company during the measurement phase;  exclusion criteria: pregnancy or skin sensitivities or plaster allergies  Response rate:  115 of 6451 (1.8%*)  *self-calculated | Exposure:  days working from home (n= 256) of 102 participants  Comparison:  days working at the office (n=219*) of 102 participants (within comparison)  Method for measuring exposure and comparison:  In their daily diaries, workers were asked: “Where did you work most of the time today?”. Possible answers were: “at the company”, “mobile work at home”, “other mobile work” (workplace).  *self-calculated | Primary outcome:  SB, PA  Definition and assessment SB:  Sedentary time […] and STS during work time and total time awake were measured objectively with the activPAL3  Definition and assessment PA:  […] time spent [...] being physically active, as well as count of steps were measured objectively with the activPAL3  Secondary outcomes:  - |
| **Scurati et al.**  2024  Italy  no study name  (no underlying cohort)  01-05/2023*  *personal communication | Study design:  Cross-sectional  Measurement:  Surveys and accelerometric measurement  Recruitment method:  The participants were employees of the University of the present study’s authors and some private companies in Milan and the hinterland. The call for participation was publicized through dissemination of the study’s information to employee associations and, in the private companies, by mail in agreement with the employers. | n=56 (f: 30 (54%); m: 26 (46%)), based on table 1  Age:  mean age: 40.5 yrs. (SD:11.8)  Eligibility criteria:  1) have an office job; 2) have a working week with at least 6  hours of daily sedentary work; and 3) be in good health and  without conditions that could limit physical activity  Response rate:  not indicated | Exposure:  Hybrid workers (at least four hours per week must have been spent working remotely at home)  n=25 (45%)  Comparison:  on-site workers  n=31 (55%)  Method for measuring exposure and comparison:  The ordinary participants’ working habits, namely the number of working days and hours spent regularly in remote (at home) or on-site working, were surveyed to categorize them as hybrid and on-site worker […] To be categorized as a hybrid worker for this study, at least four hours per week must have been spent working remotely at home. | Primary outcome:  SB, PA  Definition and assessment SB:  Sitting activity (min/day) assessed by IPAQ  Sedentary time (min/week) measured by accelerometer (Axivity AX3)  Definition and assessment PA:  Total, vigorous, moderate, walking activity in MET assessed by IPAQ  Total, vigorous, moderate and light activity in min/week measured by accelerometer (Axivity AX3)  Secondary outcomes:  Physical efficiency, self-efficacy, general well-being, job satisfaction, workload, recovery status, clinical parameters, eating habits, sleep quality |
| **Sers et al.**  (2023)  Germany (area of Karlsruhe)  no study name  (no underlying cohort)  time of data collection is not reported | Study design:  observational study  Measurement:  accelerometric measurement of PA via Move4 and ecological momentary assessment (EMA) via a smart phone provided by the study  Recruitment method:  Not reported | n=55 (f:34 (64.2%), m:19 (34.5%) values based on table 1)  Age:  35.9 +/-10.5 years  Eligibility criteria:  predominantly office-based work; having the possibility to WFH and WAO; no restrictions in performing daily activities (i.e., no disease or injury)  Response rate:  not reported  #recruited: 64  #included in the analysis: 55 | Exposure:  days working from home of 55 participants  n=141 (51.1%*)  Comparison:  days working at the office of 55 participants (within comparison)  n=135 (48.9%*)  Method for measuring exposure and comparison:  location of work (home vs. office) was specified with the first e-diary prompt of the EMA)  *Self-calculated | Outcomes:  PA/SB  Definition and assessment PA:  Based on the parameters “body position” and “energy expenditure” (MET), as well as in line with the international definition of SB and published thresholds to further specify PA into LPA and MVPA, we classified each minute of the data file obtained by Move4 accelerometric measurement as either sleep, LPA, MVPA or SB. Moreover, we categorized SB and PA time in accordance with previous research into bouts (periods of uninterrupted SB/PA time) as follows:  (a) ≤5 min = short bouts,  (b) 5–19 min = short-to-moderate bouts,  I 20–39 min = moderate-to-long bouts and  (d) ≥ 40 min = long bouts.  We aggregated all parameters either as sum or mean per day  Secondary outcome:  - |
| **Silva et al.**  2021  ConVid – Behavior Survey  (no underlying cohort)  24/04-24/05/2020 | Study design:  cross-sectional study  Measurement:  retrospective online survey with a virtual questionnaire  Recruitment method:  chain sampling procedure (researchers invite others from their social network) obeying a stratification by sex, age level and educational level) | n=39 693  (f: 19053 (48%); m: 20640 (52%))  Age:  no mean age presented  no age distribution presented  Eligibility criteria:  Brazilian adults (18+)  note: sample was weighted according to characteristics from the Brazilian National Household Sample, considering population in each state, education, age, sex, and prevalence of chronic diseases, aiming to let the sample nationally representative.  Response Rate:  n. a. due to sampling method | Exposure:  home office  Comparison:  work in normal routine  Method for measuring exposure and comparison:  working status during the pandemic was used (normal, home office, no working for any reasons) | Outcome:  PA/Physical inactivity before and during COVID-19 pandemic  Definition and assessment PA:  PA was assessed by questionnaire: “How many days a week did you practice any type of physical exercise or sport? (Do not consider physical therapy)”. Possible answers were: (a) less than 1; (b) 1-2; (c) 3-4; or (d) 5 or more. For those reporting PA practice, it was also asked: “How long did this activity last?”. Possible answers were: (a) less than 30 minutes; (b) 30-45 minutes; (c) 46-60 minutes; or (d) more than  60 minutes.  Physical inactivity was classified as less than 150 minutes/week of PA (calculated using the median point of frequency and duration in each category)  Secondary outcome:  - |
| **Suzuki et al.**  2025  Japan  No study name  (no underlying cohort)  04-05/2020 | Study design:  Cross-sectional  Measurement:  Questionnaires    Recruitment methods:  not indicated; patients with obstructive sleep apnea (OSA) who were treated with continuous positive airway pressure (CPAP) were enrolled in this study | n=666 (f:568 (85.3%*), m: 98 (14.7%*), based on figure 1, 663 were included in the analysis  Age:  61.4 years (SD 12.8) based on figure 1  Eligibility criteria:  Exclusion criteria:  (I) age <20 years; (II) treatment for sleep-disordered breathing, including the use of oral appliances and upper airway surgery during the study; (III) pollen nasal allergy; (IV) primary insomnia and other sleep disorders; and (V) inability to answer questions due to cognitive impairment  Response rate:  Not indicated  *self-calculated | Exposure:  Fully WFH  n=178 (26.8% of 663 persons) hybrid working  n=207 (31.2% of 663 persons)  fully WFH and hybrid working were categorized in the homegroup (n=385*, 58.0%*)  Comparison:  Commuting (without telecommuting at home)  n=121 (18.3% of 663 persons)  Other groups:  Unemployed  n=157 (23.7% of 663), not included in the analysis for workers  Method for measuring exposure and comparison:  Patients were queried: “During first state of emergency for the coronavirus disease 2019 (COVID-19) pandemic, your work style was ...” Possible answers were: “Work from home (WFH)”, “WFH and commuting”, “commuting” or “unemployed”.  *self-calculated | Primary outcome:  PA  Definition and assessment of PA:  Participants were queried: “how has your life changed during the COVID-19 pandemic?”, Exercise: “decreased”, “unchanged”, increased”  Secondary outcomes:  Amount of food, alcohol consumption, sleep time, subjective stress, CPAP (continuous positive airway pressure) adherence |
| **Thralls Butte et al.**  2023  USA  no study name  (no underlying cohort)  time of data collection not reported | Study design:  cross-sectional study  Measurement:  accelerometric measurement via activPAL™ for at least 4 days,  self-reported online survey  Recruitment method:  via e-mail and social media flyer announcements at various university functions and business contacts | n=53 (f: 28 (56%); m: 25 (44%))  Age:  mean age: 41.0 yrs.  Eligibility criteria:  adult (24-64 yrs.); employees with full time (≥40h/week); sedentary jobs  Response rate:  n. a. due to sampling | Exposure:  working from home  n=19 (40%)  Comparison:  working from an office  n=14 (30%)  Other study groups  working from home and from office  n=11 (22%)  other  n=3 (6%)  Method for measuring exposure and comparison:  self-report | Outcome:  PA/SB  Definition and assessment PA:  levels for moderate-to-vigorous aerobic, muscular strengthening and flexibility PA were assessed using the self-report Godin PA Questionnaire; aerobic activity was summarized to minutes per week, while resistance and flexibility training were summarized as days per week.  Definition and assessment SB:  objectively measured by activPAL™ device (ActivPAL™ micro-3, Glasgow, Scotland)  Number of sit-to-stand (STS) transitions, number of steps, and time (min/d) spent stepping, sitting, and standing for each day, sitting time (h(day) was also self-reported  Secondary outcome:  posture low back pain (LBP) |
| **Tomonaga et al.**  2024  Japan  no study name  (no underlying cohort)  06/2022-03/2023 | Study design:  cross-sectional study  Measurement:  triaxial accelerometer measurement over 5 days, questionnaires asking for worktime, place of telecommuting and lifestyle factors  Recruitment method:  white collar worker from a large company in Japan were invited to participate | n=94 m: (100%))  participants for analysis  Age:  44.6±11.5  Eligibility criteria:  white collar workers from a manufacturing company; being engaged in both work in office and work from home,  Response rate  60%, 250 from 413 agreed to participate | Exposure  work from home  n=94  Comparison  work in office  n=94  Method for measuring exposure and comparison  daily diary to distinguish days working at the office and days working from home.  On work-in-office days, they were  asked to wear the device from the time they left for work until they returned  home. On work-from-home days, they were asked to wear the device during  their working hours. The working hours included the lunch breaks. | Outcome:  PA during working hours (including lunch break) and PA during working hours (including lunch break and commuting time)  Definition and assessment PA:  metabolic equivalents (METs) in every minute and energy consumption, measured by Active style Pro HJA-750C; Omron Healthcare, Kyoto, Japan  Definition and assessment SB:  total sitting and standing time, sedentary breaks, sitting for longer than 30 min or 60 min, measured by Active style Pro HJA-750C  Secondary outcome:  - |
| **Wahlström et al.**  2024  Sweden  no study name  (Flexible Work: opportunities and challenges (FLOC) cohort)  10-12/2020, 05-06/2020 and 11-12/2020 | Study design:  cross-sectional study  Measurement:  accelerometric measurement of PA and SB over 7 days and questionnaires on physical activity, health and psychosocial factors at work  Recruitment method:  invitation of workers of 3 organisations in mid Sweden, one large industrial company and two sectors of a municipality | n=165 (f: 98 (59.4%), m: 67 (40.6%))  Age:  42.5 (SD 10.7)  Eligibility criteria:  Office workers with permanent fulltime contracts, who worked in dayshifts and reported to work hybrid  Response rate:  underlying cohort: not reported  presented study: not reported | Exposure  working at home  n=165 with 311 measured days  Comparison  working at the office  n=165 with 298 days  Method for measuring exposure and comparison  each workday, participants reported if work was performed predominantly, defined as at least 50% of the working day, at home (categorized as WFH) or in the office (categorized as WAO). | Outcome  PA and SB  Definition and assessment SB and PA:  sitting and non-sitting (standing, walking, stair walking, running, and cycling) behaviors, including discriminating between sitting uninterruptedly for different bout durations  measurement by using a thigh-worn accelerometer (Axivity AX 3, Axivity Ltd., Newcastle, UK)  Secondary outcome:  - |
| **Wallmann-Sperlich et al.**  2023  Germany  no study name  (no underlying cohort)  03-05/2021 | Study design:  cross-sectional study  Measurement:  questionnaire-based telephone survey  Recruitment method:  based on the ADM (“Arbeitskreis der deutschen Markt- und Sozialforschungsinstitute”) pool for telephone samples, sample stratified according to age and gender, weighted for the German population | n=575 (f: 258 (54.8%); m: 317 (55,2%))  Age:  mean age: 44.7 yrs.  Eligibility criteria:  working, indicating, that their work in the homeoffice is more than 0%; aged 18+  Response rate:  overall study sample: 6,7% (invited: not reported; baseline: not reported) | Exposure:  currently working in home office, average proportion of homeoffice, regarding weekly working hours  Comparison:  not working in home office  Method for measuring exposure and comparison:  participants were asked if they currently work from home with the answer options ‘yes’, ’no’, ’I work from home as well as from the office’  participants who affirmed working partially or completely from home were asked regarding the proportion of homeoffice work | Outcome:  SB, PA  Definition and assessment Sitting and PA:  Occupational Sitting and PA questionnaire (OSPAQ) and Global PA Questionnaire (GPAQ)  Definition and assessment breaks in sitting:  ‘How many breaks from sitting (such as standing up, stretching, or taking a short walk) during one hour of sitting would you typically take at work?’ with the answer options ‘5 or more, 4, 3, 2,1, 0’.  Secondary outcome:  - |
| **Webber et al.**  2024  USA  no study name  (SummerStyles)  21/05-6/06/2022 | Study design:  cross-sectional study  Measurement:  questionnaires  Recruitment method:  panel members are randomly recruited via a probability-based sampling strategy according to home address, resulting in a representative sample of the noninstitutionalized US population with approximately 60,000 panelist; the survey randomly invited 5990 participants | n=2391 (f: 1978 (45.3%), m: 1313 (54.7%))  Age:   \| Age, years \| n (weighted %) \| \| --- \| --- \| \| 18-44 \| 1,052 (55.6) \| \| 45-64 \| 1,091 (37.1) \| \| ≥65 \| 248 (7.3) \|   Eligibility criteria:  Adult participant and working (with complete dataset)  Response rate:  Underlying cohort: SummerStyles 69.3% (n=5,990 invited, n=4,151 responded)  Presented study: not reported  Note:  Working participants of SummerStyles: n=2,462 (59,3%* of 4,151 and n=2,391 included in the analysis, due to full dataset)  *self-calculated | Exposure:  more telework  n=789 (32.0%)  Comparison:  stable telework (incl. never telework n=856 (37.5%), or the same amount n=545 (22%)) resulting in  n=1,401* (59.5%*)  less telework  n=201 (8.5%)  Method for measuring exposure and comparison:  Participants were asked:  “In the past year, compared to the year before the COVID-19 pandemic, have you changed the amount you do the following activities?” “Teleworking or working remotely” with the possible answers: “I do more of this activity,” “I do the same amount of this activity,” “I do less of this activity,” or “I have never done this activity”.  *self-calculated | Outcomes:  PA  Definition and assessment PA:  Participants were asked: “In the past year, compared to the year before the COVID-19 pandemic, have you changed the amount you do the following activities?” It then listed the following three physical activities: (1) “Walking o biking to travel to and from places” (i.e., active transportation); (2) “Using parks, trails, or greenways for physical activity”; (3) “Being physically active or exercising for any reason” (i.e., any PA).  Furthermore, leisure time aerobic PA during a “usual week” was assessed based on Questions from the National Health Interview Survey. Weekly duration of moderate-to-vigorous PA (MVPA) was calculated as the sum of vigorous minutes multiplied by two and moderate minutes. According to the Physical Activity Guidelines for Americans, Second Edition, which defines recommended aerobic PA as reporting ≥150 min/week of MVPA, participants were categorized into meeting the guidelines or not.  Secondary outcome:  - |
| **Widar et al.**  2021  Sweden  no study name  (underlying cohort^[[11]](#footnote-11)^)  08/2018-06/2019 | Study design:  cross-sectional study  Measurement:  five consecutive workdays (Mo-Fr) of accelerometery measurements (AX3; diary to distinguish workplace (home or office) and time (work or leisure)  Recruitment method:  there was a previous questionnaire study, where respondents were asked about interest in participating | n=23 (f: 12 (52%); m: 11 (48%))  Age:  mean age: 47.2 yrs.  Eligibility criteria:  academic teaching and research staff (engaged in teaching and/or research ≥50% of their working time), regularly practice in telework, and do so at least one day during measurement period  Response rate:  underlying cohort: 24 Swedish universities were invited, 6 of them agreed (25%)  392 academics responded to the survey, yielding a response rate of 14% (7–21%  across the universities)  presented study: from 111 respondents, 108 were eligible and 23 agreed to participate | Exposure:  telework  (work performed during regular workhours from the participant´s home)  n=23  Comparison:  office  (work performed during regular workhours at the conventional workplace)  n=23  Method for measuring exposure and comparison:  Participants were asked if they work partially or completely from home or if they work from the office, they were also asked about their current average proportion of home office. | Outcome:  PA/SB  Definition and assessment PA:  number of sit to stand transitions during work and leisure time per day measured by accelerometer  Definition and assessment SB:  averaged time (min) spent in SB (i.e., sitting, lying) per day  measured by accelerometer  Secondary outcome:  psychophysiological reactivity: pulse recordings (heart rate, heart rate variability), cortisol concentration in saliva, stress, fatigue, recuperation |

**Table A 2.** Extracted Results

| **Study information** | **Statistical methods and confounding** | **Results** | **Comments** |
| --- | --- | --- | --- |
| **Abed Alah et al.**  2022  Qatar  no study name  (No underlying cohort)  04/01-28/2/2021 | Statistical methods:  Wilcoxon Signed Rank Test (to test the differences in sitting/reclining, and exercise times as expressed before and during the home confinement measures) and Mann-Whitney U test (to compare ordinal and not normally distributed continuous variables between those who worked from home (WFH) and those who worked at their usual place of employment).  Confounder:  not considered | Differences in Multiple Lifestyle Aspects (Presented as a Difference Between Before and During Home Confinement Measures) Between the Work from Home and Working Regularly Groups (based on Table 3)   \|  \| **WFH Group (n=565)** \| **Working regularly group (n=496)** \|  \| \| --- \| --- \| --- \| --- \| \| Variable \| Mean Ranks \| Mean Ranks \| p-value** \| \| **Sitting/reclining time difference (h/d)*** \| 586.5 \| 474.7 \| <0.001 \| \| **Exercise time difference (h/d)** \| 536.3 \| 525.4 \| 0.537 \|   *total time, not just during work  **using Mann-Whitney U test; p-values of <0.05 were considered significant   - comparing the differences in the before and during home confinement measures between the WFH and working regularly groups, higher proportions of participants reported greater increase in sitting/reclining times among the WFH group (p<0.001) - no significant difference was found with regard to exercise time between the groups (Table 3) 🡪 presented as mean ranks and Mann-Whitney U test (p values of <0.05 were considered significant)   note: supplementary table is not considered, because only changes before and during lockdown are considered | Funding:  none  Conflicts of Interest:  the authors declare no conflict of interest for this article  Overall RoB:  high (-) |
| **Barone Gibbs et al.**  2021  USA  no study name  (Reducing Sedentary Behavior on Blood Pressure (RESET BP))  05-06/2020 | Statistical methods:  ANCOVA (to compare changes in measures ‘before COVID-19’ to ‘during COVID-19’ across the three groups defined by change in remote work practices) and Kruskal–Wallis test (for heavily skewed moderate-to-vigorous intensity PA data)  Confounder:  not considered | Longitudinal change on SB and PA by remote working category (based on Table 4)   \|  \| **Remote work practices** \| **always remote (n=15)** \| **changed to remote (n=74)** \| **never remote (n=14)** \| **p-value** \| \| --- \| --- \| --- \| --- \| --- \| --- \| \| **SB** \| workday, h/day \| 0.8 (6.9) \| 0.5 (4.0) \| 0.5 (4.1) \| NS \| \| non-workday, h/day \| 2.6 (4.9) \| 0.9 (2.7) \| 1.3 (3.2) \| p <0.05 \| \| **PA** \| moderate activity, min/week \| -5 [60, 180] \| 0 [-60, 65] \| 0 [0, 45] \| NS \| \| vigorous activity, min/week \| 0 [0, 0] \| 0 [0, 0] \| 0 [0, 0] \| NS \| \| moderate-to-vigorous activity, min/week \| 35 [-60, 180] \| 10 [-64, 90] \| 0 [0, 45] \| NS \|   the difference between the always remote and never remote group is relevant for the research question of interest; data are reported as mean (SD) or median [25th, 75th percentile]; sample size varies from 88 to 103; NS – non significant  workers who were remote before and during the pandemic had the greatest increase in non-workday SB with greater declines in physical functioning (Table 4)   - always remote group had a higher increase in SB on workday and non-workday never remote group - always remote group had a higher decline in moderate activity than the never remote group - no difference between both groups in vigorous activity   always remote group had a higher increase in moderate-to-vigorous activity than the never remote group | Funding:  The National Institutes of Health (R01HL147610, R01HL134809 and P30AG024827).  Conflicts of Interest:  the authors declare no conflict of interest for this article  Overall RoB:  high (-) |
| **Bérard et al.**  2021, 2022^[[12]](#footnote-12)^  France  PSYCOV-CV  (MONALISA)  17/04-10/05/2022 | Statistical methods:  multivariate logistic regression and a stepwise selection procedure  Confounder:  each Odds Ratio (OR) was adjusted for female gender, rural home, job | Factors independently and significantly associated with decrease in PA of ≥15 min/week during the COVID-19 lockdown (17/03/2020 to 10/05/2020, France) (based on Table 1)   \|  \| **Factors independently associated with decrease in PA (N=194/536)** \| \| \| --- \| --- \| --- \| \|  \| Adjusted Odds-Ratio [95% Confidence Interval] \| p-value \| \| Job (out of home) without in-person contact with the public during lockdown (e.g., dustmen) \| 1.00 \|  \| \| Job at home during lockdown (e.g., teleworking) \| 1.96 [0.69-5.58] \| 0.209 \|   The adjusted Odds of having a decrease in PA is 1.96 higher in persons who are working at home during lockdown compared to persons who have a job (out of home) without in-person contact (results not significant). | Funding:  This study was funded by a grant from the Toulouse University Hospital, local grant 2020, Nr.RC31/20/0135.  Conflicts of Interest:  the authors declare no conflict of interest for this article  Overall RoB:  high (-) |
| **Cobbold et al.**  2023  Sydney, Australia  no study name  (Sydney Travel and Health Study (STAHS))  09-11/2019  10-12/2020  10-11/2021 | Statistical methods:  Linear mixed-effects regression models were used to determine minutes of PA in 2020 and 2021, relative to 2019 as the reference value, including participant ID as a random effect.  Confounder:  age, gender, education, and neighbourhood walkability | Mixed-effects models of weekly self-reported PA in 2020 and 2021, compared to 2019 (based on table 3)   \|  \|  \| **Weekly total PA, min** \| **Weekly walking PA, min** \| **Weekly MVPA, min** \| \| --- \| --- \| --- \| --- \| --- \| \| Sample stratification \| Wave \| Estimate [95% CI] \| Estimate [95% CI] \| Estimate [95% CI] \| \| WFH compared to pre-pandemic \|  \|  \|  \|  \| \| Less or same \| 2019 \| Ref. \| Ref. \| Ref. \| \| 2020 \| -14.7 (-82.4 to 53.0) \| -.2 (-33.0 to 32.6) \| -13.5 (-61.1 to 34.1) \| \| 2021 \| 46.4 (-30.0 to 122.8) \| 8.1 (-28.2 to 44.4) \| 39.5 (-14.5 to 93.4) \| \| More \| 2019 \| Ref. \| Ref. \| Ref. \| \| 2020 \| -13.5 (-50.1 to 23.1) \| 3.8 (-17.2 to 24.9) \| -14.0 (-41.2 to 13.2) \| \| 2021 \| **45.3 (5.5 to 85.1)** \| 21.4 (-1.0 to 43.9) \| 26.9 (-2.4 to 56.3) \|  - in the “less or same” and the “more” working from home groups, weekly total PA increased in 2021 about 45 min - in the “more” working from home group, weekly walking PA increased in 2021 by about 21 min compared to 8 min in the “less or same” working from home group - in the “less or same” working from home group, weekly MVPA in min increased in 2021 about 40 min compared to 27 min in the “more” working from home group | Funding:  No funding was received for this research.  Conflicts of Interest:  The authors declare no conflicts of interest. Chris Rissel is an Editorial Board member of HPJA and a co-author of this article. To minimise bias, they were excluded from all editorial decision-making related to the acceptance of this article for publication.  Overall RoB:  high (-) |
| **De Oliveira da Silva Scaranni et al.**  2023  Brazil  no study name  (Brazilian Longitudinal Study of Adult Health (ELSA-Brasil))  07/2020-02/2021 | Statistical methods:  logistic regression models, expressed as odds ratios (OR) and 95% confidence intervals (95%CI) to assess the association between WFH and SBs, leisure-time and domestic PA  Confounder:  age, sex, schooling, race/ethnicity, marital status, per capita income, smoking | Association between WFH, SBs, physical inactivity (leisure time), and domestic PA during the COVID-19 pandemic (based on Table 2)   \|  \|  \| **Sitting time > 8 h/day**  **OR (95% CI)** \| **Physical inactivity (leisure time)**  **OR (95% CI)** \| **High domestic physical activity**  **OR (95%CI)** \| \| --- \| --- \| --- \| --- \| --- \| \| Model 1 \| Does not perform WFH \| 1 \| 1 \| 1 \| \| Performs working from home \| **4.04**  **(3.24, 5.04)** \| **0.65**  **(0.52, 0.82)** \| **0.72**  **(0.59, 0.87)** \| \| AIC \| 3331.2 \| 2906.7 \| 3518.8 \| \| Model 2 \| Does not perform WFH \| 1 \| 1 \| 1 \| \| Performs working from home \| **2.98**  **(2.33, 3.81)^a^** \| 0.93  (0.72, 1.22)^b^ \| 1.14  (0.91, 1.43)^b^ \| \| AIC \| 3257.9 \| 2839.6 \| 3333.3 \| \| Model 3 \| Does not perform WFH \| 1 \| 1 \| 1 \| \| Performs working from home \| **2.68**  **(2.02, 3.56)^c^** \| 0.99  (0.75, 1.31)^d^ \| **1.29**  **(0.99,**  **1.67)^e^** \| \| AIC \| 2644.8 \| 2579.5 \| 2715.0 \|   WFH: work from home; OR: odds ratios; CI: confidence interval; AIC: akaike information criterion  Model 1: unadjusted model  ^a^Model 2: model 1 + age + schooling  ^b^Model 2: model 1 + age + sex + schooling + income  ^c^Model 3: model 1 + age + schooling + pre-COVID sitting time  ^d^Model 3: model 1 + age + sex + schooling + income + pre-COVID leisure-time PA  ^e^Model 3: model 1 + age + sex + schooling + income + pre-COVID domestic PA  values in bold indicate statistical significance   - WFH displayed significantly higher rates of “accumulated sitting time greater than eight hours/day” and lower rates of “physical inactivity” and “high domestic PA” (pre- and post-COVID-19) when compared to participants not doing WFH (Table 2) - After adjusting for covariables, participants performing WFH showed three-fold significantly higher odds for “accumulated sitting time greater than eight hours a day” and 29% higher odds (with borderline significance) of high domestic PA compared to those not performing WFH (Table 2) - There was no association between WFH and leisure-time PA. (Table 2) - Age was an effect modifier of WFH on leisure-time (p=0.00984) and domestic (p=0.035412) PA (Table 3) - Older people working from home showed 84% higher odds of physical inactivity (OR=1.84, 95% CI: 1.07–3.16) and 92% higher odds of high domestic PA (OR=1.92, 95%CI: 1.12–3.27), compared to older people performing regular professional work (Table 4, not shown). | Funding:  Supported by Brazil’s National Research Council (CNPq) with Grants 405551/2015-0 BA, 405544/2015-4 RJ, 405552/2015-7 MG,  405543/2015-8 ES and 405545/2015-0 RS. RHG and MJMF are researcher  fellows of the National Research Council (CNPq) and also supported by a research grant (Scientists from our state) from the Research Support  Foundation of the State of Rio de Janeiro (FAPERJ), Brazil.  Conflicts of Interest:  The authors declare that they have no conflicts of interest.  Overall RoB  low (+) |
| **Delanoeije et al.**  2024  Belgium  No study name  (no underlying cohort)  04-06/2021 and 11-12/2021 | Statistical method:  Linear mixed models (two-level model)  Confounder:  Dog caregiver, outdoor access, animal caregiver, partner, education level, gender, age, social support, emotionality, season | Random Coefficent Modeling Results to Predict Daily Physical Activity (based on table 4)   \|  \| **Model 1^a^** \| \| \| **Model 4^b^** \| \| \| \| --- \| --- \| --- \| --- \| --- \| --- \| --- \| \|  \| ß \| SE \| p \| ß \| SE \| p \| \| **Telework day (1=yes)** \| 4.08 \| 3.29 \| 0.215 \| -29.96 \| 8.47 \| 0.000 \| \| **Dog caregiver (1=yes)** \|  \|  \|  \| −14.02 \| 10.89 \| 0.198 \| \| **Outdoor access (1=yes)** \|  \|  \|  \| −20.26 \| 9.19 \| 0.028 \| \| **Dog x Telework day** \|  \|  \|  \| 25.12 \| 7.63 \| 0.001 \| \| **Outdoor Access x Telework day** \|  \|  \|  \| 19.33 \| 8.20 \| 0.019 \| \| N=357 persons and 3310 data points. SE = standard error,  ^a^ adjusted for dog caregiver, outdoor access, animal caregiver, partner, education level, gender, age, social support, emotionality, season,  ^b^ adjusted for dog caregiver, outdoor access, animal caregiver, partner, education level, gender, age, social support, emotionality, season, dog caregiver X Telework day, Outdoor access X Telework day \| \| \| \| \| \| \|   Physical activity score significantly decreased on telework days for people without a dog or outdoor access by (ß: -29.96, p<0.001) | Funding:  This work was supported by the Internal Funds KU Leuven under Grant 3H200323 and the Research Foundation Flanders under Grant 12B0522N awarded to Joni Delanoeije.  Conflict of Interest:  The authors report there are no competing interests to declare.  Overall RoB:  high (-) |
| **Elangovan et al.**  2021  India  no study name  (no underlying cohort)  24/06/-03/07/2020 | Statistical methods:  Pearson’s chi-square test (to test the differences in lifestyle changes between WAO and WFH) and Cramer’s (V) test (to test the strength of the relationship between two variables)  Confounder:  not considered | Proportion of participants with different amounts of PA (h of exercise/week) working from home (WFH) and working at office (WAO) (based on Table 2)   \| **Variables** \| **WFH%** \| **WAO%** \| **total%** \| **p-value** \| **Effect size** \| \| --- \| --- \| --- \| --- \| --- \| --- \| \| **Exercise/week:** \|  \|  \|  \|  \|  \| \| within 3h \| 41.2 \| 36.2 \| 38.1 \| 0.191 \| 0.068 \| \| 3-7h \| 10.5 \| 9.7 \| 10.0 \| \| >7h \| 5.5 \| 4.4 \| 4.8 \| \| did not do any activity \| 42.8 \| 49.7 \| 47.1 \|   No significant difference was found in proportion of participants with different hours of exercise between the WFH and WAO group. (Table 2) | Funding:  not reported  Conflicts of Interest:  not reported  Overall RoB:  high (-) |
| **Fukushima et al.**  2021  Japan  No study name  (MyVoice Communication database)  28/07-02/08/2020 | Statistical methods:  chi-squared (for continuous and categorical variables)  mean time spent in SB, LPA, MVPA, and SB bout length was compared not only between the no WFH and WFH groups but also among WHF subcategories  ANCOVA was performed by adjusting for confounder listed below  for all analyses, P<0.05 were considered as statistically significant  Confounder:  age, gender, smoking status, drinking frequency, body mass index, residential area, educational attainment, self-rated health, job characteristics, industrial classification and working time | Comparison of time and proportion of each behaviour during working time between the no WFH and WFH groups (based on Table 2)   \|  \| **Adjusted model (according to ANCOVA)** \| \| \| \| \| \| \| --- \| --- \| --- \| --- \| --- \| --- \| --- \| \|  \| **Time spent in each behaviour during working time (min)** \| \| \| **Proportion of each behaviour per**  **working time (%)** \| \| \| \|  \| No WFH  (n=676) mean (SE) \| WFH  (n=477) mean (SE) \| p-value \| No WFH  (n=676) n (%) \| WFH  (n=477) n (%) \| p-value \| \| **SB** \| 179.7 (17.4) \| 256.2 (18.7) \| <0.001 \| 37.1 (3.8) \| 53.3 (4.0) \| <0.001 \| \| **LPA** \| 146.5 (12.8) \| 97.3 (13.7) \| <0.001 \| 34.6 (2.9) \| 24.0 (3.1) \| <0.001 \| \| **MVPA** \| 118.0 (10.3) \| 90.7 (11) \| <0.001 \| 27.8 (2.3) \| 21.8 (2.5) \| <0.001 \| \| **SB bout length** \| 24.0 (3.2) \| 33.6 (3.5) \| <0.001 \| - \| - \|  \|   Results for adjusted model (ANCOVA):   - during working time, sedentary time and proportion of SB was significantly higher in the WFH group than in the no WFH group (Table 2) - significantly shorter LPA and MVPA times (%) were reported in the WFH group than in the no WFH group (Table 2) - significant difference in SB bout length between the WFH and no WFH groups (Table 2)   Dose-Response-Analysis (see Fig 1)   - excluded works who were unable to work from home (e.g., essentials workers) - among the WFH subgroups, longer SB time and shorter LPA and MVPA times were observed in the highest WFH group (WFH 76%-100%) compared to the WFH 1%-25% and 26%-50% subgroups (all p< 0.05) - compared with the less WFH groups, there were significant trends (all p-values for trend were <0.05) for longer SB time and sitting bout length, and shorter LPA and MVPA time in the higher WFH groups - SB time in the highest WFH group (WFH 76%-100%:300.5 [37.5] min) was the longest compared with SB in the other groups (WFH 1%-25%, 26%-50%, 51%-75%, 235.2 [38.4], 243.5 [38.8], 258.5 [40.2] min respectively) - LPA and MVPA were significantly shorter in the WFH 76%-100% subgroup than in the WFH 1%-25% and 26%-50% subgroups, but not the WFH 51%-75% subgroup (LPA; WFH 1%-25%, 26%-50%, 51%-75%, and 76%-100%: 123.6 [24.1], 115.3 [24.4], 103.6 [25.3], and 82.8 [23.6] min, MVPA; WFH 1%-25%, 26%-50%, 51%-75%, and 76%-100%: 95.3 [22.1], 95.2 [22.3], 91.9 [23.1], and 66.2 [21.6] min)SB bout lengths in both the WFH 51%-75% and 76%-100% subgroups were significantly longer than those in the WFH 1%-25% subgroup (WFH 1%-25%, 26%-50%, 51%-75%, and 76%-100%: 32.7 [8.6], 39.0 [8.7], 44.5 [9.0], and 42.2 [8.4] min) | Funding:  supported by a grant from the Meiji Yasuda Life Foundation of Health and Welfare  Conflicts of Interest:  the authors declare no conflict of interest for this article  Overall RoB:  almost low (+/-) |
| **Grubben et al.**  2022  The Netherlands  no study name (Dutch LISS panel)  07/06-27/07/2021 | Statistical methods:  Pearson correlations of all outcome variables and predictors  ANOVA (adjusted by all covariates to test the influence of WFH on several outcomes relating to sports participation)  adjusted scores from multiple classification analyses (MCA) within ANOVA  calculation of Eta^2^ (effect size based on the proportion of variance of a variable)  logistic regression (supplement)  Confounder:  sex, age group, weekly contracted working hours, having a child (if so: the age of those children), hampered by COVID-19, agreement with the statement that sufficient sports provisions were available in their neighbourhood, educational level and economic deprivation | - WFH and sports participation were positively correlated (r=0.185, p=0.000):   - significant differences in sports participation between workers who did and did not work from home: 53% of those WFH participated in sports (p>0.001 (two tailed)), 42% of those not WFH participated in sports 🡪 substantial positive relationship between WFH and sports participation during the COVID-19 pandemic (Eta2=0.185 (large effect)) (Table 3)   - there was no difference in the results between women and men - positive association between WFH and individual sports participation (r=0.194, p=0.000), sports participation in the public space (r=0.194,) and sports participation at home (r=0.099, p=0.000) 🡪 individuals who worked from home indeed engaged more in sports, individually, at home and in the public space, than individuals who did not work from home (Table 3)   Estimates from logistic regression analysis (Supplement 2)   \|  \| **Sports participation** \|  \| \| --- \| --- \| --- \| \|  \| B \| SE \| \| **Working from home (ref.=no)** \| 0.009* \| 0.004 \|   Source: LISS (2021). N=1506; *p < 0.05  Participating in sports was more prevalent in workers working from home; working more hours from home increases the chance of participating in sport (Supplement S2). | Funding:  Netherlands Organisation for Health Research and Development, grant number: 10430032010016.  Conflicts of Interest:  the authors declare no conflict of interest for this article.  Overall RoB:  high (+) |
| **Hallman et al.**  2021  Sweden  no study name  (Flexible Work: Opportunity and Challenge (FLOC) cohort)^[[13]](#footnote-13)^  05-07/2020 | Statistical methods:  a repeated-measures MANOVA of compositional data using type of day (two levels, WAO vs. WFH) as a within subject factor and all six isometric log ratios (ILRs) as dependent variables (to compare the 24-h time-use composition between WAO and WFH days)  mean differences with 95% confidence intervals for each of the six ILRs were calculated separately  $\eta_{p}^{2}$ was used as a measure of effect size (benchmark values of 0.099, 0.0588, and 0.1379 indicating small, medium, and large effect sizes)  Confounder:  not applicable | Arithmetic means with standard deviation between workers (SD) of time (minutes/day) spent in the investigated physical behaviors (PB) during all observed days, days working at the office (WAO days) and days working from home (WFH days) (based on Table 2)   \|  \| **All days mean (SD)**  **in min/day** \| **WAO days mean (SD)**  **in min/day** \| **WFH days mean (SD)**  **in min/day** \| \| --- \| --- \| --- \| --- \| \| **total time at work** \| 499 (185) \| 512 (165) \| 486 (205) \| \| **sedentary** \| 367 (101) \| 373 (86) \| 361 (116) \| \| **standing** \| 95 (63) \| 102 (63) \| 88 (63) \| \| **moving** \| 37 (22) \| 37 (17) \| 36 (27) \| \| **total leisure time** \| 465 (144) \| 468 (128) \| 461 (159) \| \| **sedentary** \| 257 (61) \| 258 (50) \| 256 (71) \| \| **standing** \| 142 (51) \| 141 (44) \| 143 (58) \| \| **moving** \| 66 (32) \| 70 (34) \| 62 (30) \|   Time spent in physical behaviours only differed to a minor extent between WFH and WAO. The most notably point is that days WFH showing 12 min less sedentary time at work, and 7 min less time moving during leisure than days WAO (Table2).  Statistical results from the univariate models on time use as isometric log ratios (ILR); n=27 (repeated measures MANOVA) (based on Table 3)   \| **Effect of day type** \|  \| **F-value** \| **P-value** \| $\boldsymbol{\eta}_{\boldsymbol{p}}^{\boldsymbol{2}}$***** \| \| --- \| --- \| --- \| --- \| --- \| \| Behaviours at work: \|  \|  \|  \|  \| \| ILR_3_: sedentary/non-sedentary \| 0.79 \| 0.38 \| 0.03 \| \| ILR_4_: standing/moving \| 0.02 \| 0.89 \| 0.00 \| \| Behaviours at leisure \| ILR_5_: sedentary/non-sedentary \| 0.48 \| 0.49 \| 0.02 \| \|  \| ILR_6_: standing/moving \| 1.14 \| 0.30 \| 0.04 \|   * $\eta_{p}^{2}$=partial eta squared  “ILR3 and ILR4 represent time at work spent in sedentary relative to non-sedentary behaviors (ILR3), and – within non-sedentary behaviors – time spent standing relative to time spent moving (ILR4).”  $ILR3=\sqrt{\frac{2}{3}}\ln\frac{work SED}{\sqrt[2]{workSTAND x workMOVE}}$  $ILR4= \sqrt{\frac{1}{2}}\ln\frac{workSTAND}{workMOVE}$  $ILR5= \sqrt{\frac{2}{3}}\ln\frac{leisSED}{\sqrt[2]{leisSTAND x leisMOVE}}$  $ILR6= \sqrt{\frac{1}{2}}\ln\frac{leisSTAND}{leisMOVE}$   - Days WFH and days WAO differed in their overall 24-h time-use composition (F=2.45; p=0.06; $\eta_{p}^{2}$=0.41). - WFH is clearly associated with more time spent sleeping, but has no marked influence on the proportion of time spent SB or standing or moving at work and at leisure time. | Funding:  FORTE (2019–01257); Open Access funding by University of Gävle.  Conflicts of Interest:  the authors declare no conflict of interest for this article  Overall RoB:  low (+) |
| **Henke et al.**  2016  USA  no study name  (no underlying cohort)  2010 and 2011 | Statistical methods:  used one-way analyses of variance to determine whether any differences in all study variables by telecommuter status each year were significant  estimation of general linear mixed models (GLMMs) with logit link and binomial distribution to predict risk status (high risk or not at risk) for each health risk outcome  Confounder:  all employee characteristics: including age, sex, race/ethnicity, job grade, management status, diagnosis cost grouper risk score (measure for clinical severity) and work location (note: No reporting or adjustment for working hours) | Percentage of employees with high risk for physical inactivity (less than 3x per week cardio) by telecommuting category, 2011 (based on Table 3)   \| Non-telecommuters \| Off-hour telecommuters \| prime time telecommuters \| P \| \| --- \| --- \| --- \| --- \| \| 40.4 \| 39.1 \| 38.0 \| 0.4597 \|  - Non-telecommuters had the highest percentage of employees at risk for physical inactivity. - The differences across the three telecommuter categories for physical inactivity were not significant at the .05 probability level.   Relationship Between Telecommuting Category and risk or physical inactivity, adjusting for Employee Characteristics (based on Table 5)   \| **Longitudinal Model (time nested within person)** \| \| \| **Physical Inactivity Beta-Coefficients** \| \| --- \| --- \| --- \| --- \| \| Intercept \| \| \| -0.308 \| \| telecommuting status \| Off-hour telecommuter \|  \| -0.122 \| \| Prime time telecommuter \| Low (≤8 h/mo) \| -0.189 \| \| Medium (9-32 h/mo) \| -0.249 (P<0.05) \| \| High (33-72 h/mo) \| -0.140 \| \| Very high (≥73 h/mo) \| -0.015 \| \|  \| Non-telecommuter (reference) \|  \|   Model also included management status, region and a diagnosis cost grouper risk score   - Prime time telecommuters working 9 to 32 hours per month (medium intensity) had a significantly lower risk for physical inactivity compared with non-telecommuters (35% vs. 41%). For groups working less or more hours in telework, results were not significant. | Funding:  not reported  Conflicts of Interest:  not reported  Overall RoB:  almost low (+/-) |
| **Herbolsheimer et al.**  2024  Germany  no study name  (NAKO)  30/04-12/05/2020 | Statistical methods:  multivariable linear regression models examined variations in several  physical activity domains and sedentary behavior  Confounder:  baseline physical activity, individual characteristics, social influences, and environmental characteristics, Log-binomial regressions were additionally adjusted for unemployment, living alone, regional number of covid-cases at time of the interview, month of interview | - In multivariable linear regression models, remote work was related to increased sedentary time (β=0.21; 95% CI: .203; .216), increased engagement in household chores (β = 0.16; 95% CI: .159; .171), and more recreational activities (β=0.05; 95% CI: .043; .056).^a^ - Employees shifting to remote work were significantly more likely to fall below these guidelines (OR = 2.17; 95% CI: 2.04; 2.31), while a distinct segment of remote workers began to follow them (OR = 1.47 95% CI: 1.39; 1.56), indicating a heterogeneous impact of remote work on physical activity. ^a^   ^a^personal communication | Funding:  Open Access funding enabled and organized by Projekt DEAL. This project was conducted with data from the German National Cohort (NAKO Gesundheitsstudie, NAKO) (www.nako.de). The NAKO is funded by the Federal Ministry of Education and Research (BMBF) [project funding reference numbers: 01ER1301A/B/C, 01ER1511D, and 01ER1801A/B/C/D], federal states of Germany, and the Helmholtz Association  Conflicts of Interest:  The authors declare no competing interests.  Overall RoB:  high (-) |
| **Holmes et al.**  2023  USA  no study name  (Reducing Sedentary Behavior on Blood Pressure (RESET BP))  12/2017-08/2022 | Statistical methods:  linear regression analyses to determine cross-sectional associations of the physical and social workplace environment characteristics with SB and PA.  stratified analysis repeating all regression models for a home-based strata and office-based strata  associations were considered statistically significant if p ≤0.05.  Confounder:  work wear time, age, BMI, and gender | Associations of Worksite Location and Durations of SB (SB all, SB30 in bouts ≥30min; SB60 in bouts ≥60min) and PA During the Workday (n=271) (based on Table 2).   \|  \| **SB (Minutes Per Workday)** \| **SB30 (Minutes Per Workday)** \| **SB60 (Minutes**  **Per Workday)** \| **Standing Time (Minutes Per Workday)** \| **Stepping Time (Minutes Per Workday)** \| \| --- \| --- \| --- \| --- \| --- \| --- \| \| **Home (n=91)** \| Reference \| Reference \| Reference \| Reference \| Reference \| \| **Office (n=179)** \| −17.2±8.4** \| −39.1±12.8** \| −41.3±11.8** \| 13.0±7.1* \| 3.9±2.3 \|   Results are reported as adjusted β ± standard error with adjustment for age, BMI, gender, and work wear time.  *0.05 ≤ p ≤ 0.10; **p ≤ 0.05.  Associations of Worksite location with Steps and Sit-to-Stand Transitions (n=271) (based on Table 5).   \|  \| **Steps Per Workday** \| **Sit-to-Stand Transitions Per Workday** \| \| --- \| --- \| --- \| \| **Home (n=91)** \| Reference \| Reference \| \| **Office (n=179)** \| 695.4±200.5* \| 2.1±1.3 \|   Results are reported as adjusted β ± standard error with adjustment for age, BMI, gender, and work wear time.  *p ≤0.05.   - Those, who worked at an office spent less work time in SB (−17.2 min, p=0.04), SB30 (−39.1 min, p < 0.01), and SB60 (−41.3 min, p < 0.01) when compared with those, who worked at home (Table 2). - In addition, office-based workers accumulated more steps during work hours than those working from home (+695.4 steps, p < 0.01) (Table 5).  \| **Activity levels during working hours (based on Supplemental Content 3. Participant characteristics).** \| \| \| \| --- \| --- \| --- \| \|  \| **Office-based (*n*=180)** \| **Home-based (*n*=91)** \| \|  \| **Mean (SD) or *n* (%)** \| **Mean (SD) or *n* (%)** \| \| SB, minutes per day \| 393.4 (±78.9) \| 405.4 (±94.9) \| \| SB30, minutes per day \| 226.7 (±101.4) \| 262.7 (±113.4) \| \| SB60, minutes per day \| 111.3 (±89.5) \| 151.5 (±99.8) \| \| Standing, minutes per day \| 88.3 (±60.6) \| 73.1 (±51.5) \| \| Stepping minutes per day \| 35.0 (±19.2) \| 30.6 (±18.3) \| \| Sit-to-stand transitions, per day \| 26.6 (±10.7) \| 24.0 (±10.9) \| \| Total steps, per day \| 3046.5 (±1703.0) \| 2299.5 (±1394.4) \| \| Total work time, per day (hours) \| 8.6 (±1.2) \| 8.5 (±1.4) \| \| Workdays with monitor wear \| 4.6 (±1.1) \| 4.9 (±1.2) \| \| SD, standard deviation; SB, sedentary behaviour; SB30, duration of time spent in bouts of SB ≥30 minutes; SB60, duration of time spent in bouts of SB ≥60 minutes. \| \| \| | Funding:  supported by the National Institutes of Health (R01 HL134809 and UL1TR001857).  Conflicts of Interest:  The authors declare that they have no conflicts of interest.  Overall RoB  low (+) |
| **Holmes et al.**  2025  USA  No study name  (**Re**ducing **Se**dentary Behavior on Blood Pressure (RESET BP))  12/2007-08/2022 | Statistical method:  t-tests compared means between WFH and office worksite location groups  Confounder: wear time (for device-based measures of PA and SB), age, and work sector | Comparison of PA by Worksite Location (based on table 3)   \| **Device-measured activity (n=258)** \| \| \| \| \| \| --- \| --- \| --- \| --- \| --- \| \|  \| **Office (n=170)** \| **WFH (n=88)** \| **Difference (WFH vs. Office)** \| **p-value** \| \| **MPA (min/week)** \| 243.9 (9.8) \| 213.8 (14.8) \| -30.1 (17.3) \| 0.08 \| \| ***adjusted*** \| - \| - \| **19.0 (18.4)** \| **0.30** \| \| **VPA (min/week)** \| 14.6 (2.4) \| 13.9 (3.2) \| -0.7 (4.0) \| 0.87 \| \| ***adjusted*** \| - \| - \| **0.8 (4.3)** \| **0.85** \| \| **MVPA** \| 259.5 (10.8) \| 228.9 (17.0) \| -30.6 (19.3) \| 0.11 \| \| ***adjusted*** \|  \|  \| **-17.7 (20.6)** \| **0.39** \| \| **Self-reported leisure time activity (n=258)** \| \| \| \| \| \|  \| Office (n=169) \| WFH (n=89) \| Difference (WFH vs. Office) \| p-value \| \| **MPA (min/week)** \| 66.9 (7.1) \| 65.2 (8.4) \| -1.7 (11.5) \| 0.88 \| \| ***adjusted*** \| - \| - \| **0.6 (12.4)** \| **0.96** \| \| **VPA (min/week)** \| 3.5 (1.1) \| 4.9 (2.1) \| 1.4 (2.1) \| 0.51 \| \| ***adjusted*** \| - \| - \| **1.6 (2.5)** \| **0.51** \| \| **MVPA** \| 70.1 (7.2) \| 70.3 (8.5) \| 0.2 (11.7) \| 0.98 \| \| ***adjusted*** \|  \|  \| **2.9 (12.7)** \| **0.82** \| \| **Device-measured light activity (activPAL + ActiGraph) (n=258) - LPA** \| \| \| \| \| \|  \| Office (n=170) \| WFH (n=88) \| Difference (WFH vs. Office) \| p-value \| \| **Standing (hour/day)** \| 3.63 (0.09) \| 3.20 (0.14) \| −0.43 (0.16) \| 0.01 \| \| ***Adjusted*** \| - \| - \| **−0.25 (0.17)** \| **0.14** \| \| **LPA w/o standing (hour/day)** \| 0.91 (0.03) \| 0.84 (0.04) \| −0.07 (0.05) \| 0.16 \| \| ***Adjusted*** \| - \| - \| **−0.04 (0.05)** \| **0.51** \| \| **LPA + Standing (hour/day)** \| 4.55 (0.11) \| 4.04 (0.17) \| −0.51 ± 0.20 \| 0.01 \| \| ***Adjusted*** \| - \| - \| **−0.29 ± 0.20** \| **0.16** \|   *Data are reported as least square means (standard errors) with adjustment for age, work sector, and objective monitor wear time (where applicable). LPA, light physical activity; MPA, moderate physical activity; MVPA, moderate to vigorous physical activity; VPA, vigorous physical activity.*   - Neither device-measured nor self-reported MPA, VPA nor MVPA were different across groups in unadjusted or adjusted models (all p>0.05)   Comparison of SB by Worksite Location (based on table 4)   \| **Device-measured (activPAL) (n=261)** \| \| \| \| \| \| --- \| --- \| --- \| --- \| --- \| \|  \| Office (n=172) \| WFH (n=89) \| Difference (WFH vs. Office) \| p-value \| \| **SB (hour/day)** \| 11.02 (0.12) \| 11.11 (0.18) \| 0.09 (0.20) \| 0.66 \| \| ***Adjusted*** \| - \| - \| **0.37 (0.21)** \| **0.08** \| \| ***SB60 (hour/day)*** \| 3.21 (0.14) \| 3.75 (0.24) \| 0.54 (0.25) \| 0.03 \| \| ***Adjusted*** \| - \| - \| **0.77 (027)** \| **0.01** \| \| **Self-reported (SBQ) (n=261)** \| \| \| \| \| \| **Total SB (hour/day)** \| 9.97 (0.25) \| 10.15 (0.36) \| 0.18 (0.43) \| 0.68 \| \| ***Adjusted*** \| - \| - \| **0.38 (0.46)** \| **0.41** \|   *Data are reported as least square means (standard errors) with adjustment for age, work sector, and objective monitor wear time (where applicable). rec., recreational; SB, sedentary behavior; SB60, duration of time spent in bouts of sedentary behavior ≥60 minutes; SBQ, Sedentary Behavior Work/Non-Workday Questionnaire.*   - WFH workers accumulated significantly greater time in prolonged sitting (SB60-minute bouts) as assessed by device-measured SB60 compared to office workers in the adjusted model | Funding:  This research was supported by the National Institute of Health R01HL134809, R01 HL147610, and UL1TR001857  Conflict of Interest:  The authors have declared no competing interest.  Overall RoB:  low (-) |
| **Ishibashi et al.**  2022  Japan  no study name  (no underlying cohort)  03-25/08/2020 | Statistical methods:  descriptive analysis  Confounder:  not considered, stratification for age and gender | - commuters have much more PA associated with daily trips than teleworkers do, irrespective of the time point (before, during, after the pandemic) (Figure 5) - commuting behaviour is a driving factor of the amount of PA associated with daily trips for workers (Figure 5) | Funding:  JSPS Grant-in-Aid for Scientific Research (20H02265)  Conflicts of Interest:  The authors declare no conflict of interest for this article.  Overall RoB:  high (-) |
| **Kikuchi et al.**  2025  Japan  No study name  (no underlying cohort)  05/2025 | Statistical method:  Paired t-test in overall participants and then in stratified analysis by individual factors  Confounder:  Gender, age, living arrangement, commuting mode | Based on table 1, table 3 and personal communication: Average number of steps per day and daily time of sedentary behavior (SB) by working location (n=177)   \|  \| **WAO** \| **WFH** \| **p-value** \| \| --- \| --- \| --- \| --- \| \| **PA** \|  \|  \|  \| \| Average of step count (per day) \| 8046 (2586) \| 3284 (2908) \| <0.001 \| \| LPA in min/day (SD) \| 197 (70.0) \| 156.1 (79.1) \| <0.001 \| \| MPA in min/day (SD) \| 60.7 (20.9) \| 27.2 (23.9) \| <0.001 \| \| VPA in min/day (SD) \| 1.6 (4.5) \| 2.5 (7.7) \| <0.05 \| \| **SB** \|  \|  \|  \| \| SB in min/day (SD) \| 694.9 (121.9) \| 715.5 (147.3) \| <0.001 \|  - The average number of steps taken was 8046 steps/day for WAO and 3284 steps/day for WFH, indicating that workers engage in 59.2% (4,792 steps/day) less activity in WFH than in office work - Compared to WAO, a 6.6% higher proportion of SB and 3.4% lower proportions of LPA and MPA were observed (p<0.001) | Funding:  Hiroyuki Kikuchi was supported by the Grant-in-Aid for Scientific Research (C) 23K10597 from the Japan Ministry of Education, Culture, Sports, Science and Technology. This work was supported by the Ministry of Health, Labor and Welfare Japan (Program Grant Number 22JA1005.)  Conflicts of Interest:  the authors declare no conflict of interest for this article  Overall RoB:  almost low (+/-) |
| **Kim et al.**  2022  Japan  no study name  (no underlying cohort)  01-03/2021 | Statistical methods:  baseline characteristics as means (with standard deviations) for continuous variables or as frequencies and percentages for categorical variables  Confounder:  not considered | Sub-domain analyses on commuting day and remote working day (n=34) (based on Table 4).   \|  \| Week 0 \| \| --- \| --- \| \| **PA outcomes on commuting working day** \|  \| \| MVPA, min/day (SD) \| 58.5 (27.5) \| \| LPA, min/day (SD) \| 179.7 (72.8) \| \| MPA, min/day (SD) \| 58.1 (27.6) \| \| VPA, min/day (SD) \| 0.4 (0.8) \| \| Steps, steps/day (SD) \| 8215 (2681) \| \| **Sedentary Time on commuting working day** \|  \| \| ST, min/day (SD) \| 627.8 (98.8) \| \| **PA outcomes on remote working day** \|  \| \| MVPA, min/day (SD) \| 34.1 (33.9) \| \| LPA, min/day (SD) \| 175.9 (68.6) \| \| MPA, min/day (SD) \| 32.9 (33.7) \| \| VPA, min/day (SD) \| 1.5 (5.5) \| \| Steps, steps/day (SD) \| 4261 (3526) \| \| **Sedentary Time on remote working day** \|  \| \| ST, min/day (SD) \| 610.0 (104.6) \|   notes: Data are presented as mean (standard deviation) for continuous variables or number (%) for categorical variables at week 0; LPA, light PA; MPA, moderate-to-vigorous PA; MVPA, moderate-to-vigorous PA; ST, sedentary time; VPA, vigorous PA.  a statistical analysis of the difference between behaviour on days on commuting days and days in remote work at week 0 was not performed | Funding:  supported in part by JST SPRING (JPMJSP2124) and the Advanced Research Initiative for Human High Performance, University of Tsukuba.  Conflicts of Interest:  K.N. and M.M. were employed by MS&AD InterRisk Research & Consulting, Inc. The remaining authors declare that they have no conflicts of interest  Overall RoB  high (-) |
| **Kitano et al.**  2024  Japan  No study name  (Meiji Yasuda LifeStyle Study)  04/2022-03/2023 | Statistical method:  multiple linear regression analysis  Confounders:  Age, gender, education, number of children, job type, employment status, weekly working hours, BMI, relf-rated health, psychological distress, stage of change for exercise and eating habits and accelerometer wear time | Estimated marginal means with 95% CI (based on personal communication)   \|  \| **Never WFH** \| **1-2 days WFH** \| **3-4 days WFH** \| **≥5 days WFH** \| \| --- \| --- \| --- \| --- \| --- \| \| **PA** \|  \|  \|  \|  \| \| LPA (min/day) \| 250.5 (241.2-259.8) \| 215.3 (203.0-227.7) \| 208.2 (195.2-221.1) \| 205.2 (193.9-216.4) \| \| MVPA (min/day) \| 55.1 (52.0-58.1) \| 49.7 (45.6-53.8) \| 37.3 (33.0-41.5) \| 27.9 (24.2-31.6) \| \| Total PA (min/day) \| 305.6 (295.3-315.8) \| 265.1 (251.5-278.7) \| 245.5 (231.3-259.6) \| 233.0 (220.6-245.5) \| \| Step counts (steps/day) \| 7200.0 (6855.7-7544.2) \| 6214.5 (5750.8-6678.2) \| 4413.9 (3934.4-4893.3) \| 3207.5 (2790.4-3624.5) \| \| **SB** \|  \|  \|  \|  \| \| Time in SB (min/day) \| 584.5 (574.3-594.8) \| 625.0 (611.4-638.6) \| 644.6 (630.4-658.8) \| 657.0 (644.6-669.5) \| \| Prolonged SB (min/day) \| 258.9 (240.8-277.0) \| 315.1 (291.2-339.1) \| 357.9 (332.6-383.2) \| 374.2 (352.2-369.1) \| \| Prolonged SB counts (times/day) \| 4.5 (4.2-4.7) \| 5.4 (5.1-5.7) \| 6.0 (5.6-6.3) \| 6.1 (5.8-6.4) \| \| Prolonged SB bout duration (min/time) \| 56.1 (51.4-60.7) \| 56.8 (50.5-63.1) \| 59.3 (52.8-65.8) \| 63.6 (58.0-69.3) \| \| Adjusted for age, gender, education, number of children, job type, employment status, weekly working hours, BMI, self-rated health, psychological distress, stage of change for exercise and eating habits and accelerometer war time \| \| \| \| \|  - Regardless of PA intensity, individuals who work from home even 1 to 2 days per week engaged in less PA than those who did not (P < .05). - Time spent on MVPA, total PA and step counts decreased as the weekly frequency of WFH increased. - More frequent WFH was associated with more time spent in SB, prolonged SB, and daily counts of prolonged SB bouts (P < .05). | Funding:  This work was partially  supported by the Japan Society for the Promotion of Science Grant-in-Aid  for Young Scientists (17K13238; 18K17930; 20K19701), a Grant-in-Aid  for Scientific Research (19K11569), and a Health Labor Sciences Research  Grant (22JA0501).  Conflict of Interest:  Not indicated  Overall RoB:  almost low (+/-) |
| **Koohsari et al.**  2021  Japan  no study name  (no underlying cohort)  22/02/2019 and 08/07/2020 | Statistical methods:  multivariable linear regression models were used to estimate the associations between changes in work style and changes in sedentary behaviours and physical activities  Confounder:  Age, sex, marital status, highest education, and gross annual household income and change in working days before and after the outbreak of COVID-19. | Associations between absolute changes in working from home days and changes in workers’ domain-specific sedentary behaviours (complete data of 1086 individuals) (based on table 3)   \|  \| **Car Sitting time (h/d)** \| **Public Transportation Sitting (h/d)** \| **Work-related Sitting-Time (h/d)** \| **TV Viewing Time (h/d)** \| **PC Use Sitting time (h/d)** \| **Other Leisure Sitting Time (h/d)** \| **Total Sitting Time (h/d)** \| \| --- \| --- \| --- \| --- \| --- \| --- \| --- \| --- \| \|  \| b (95% CI)  p-Value \| b (95% CI)  p-Value \| b (95% CI)  p-Value \| b (95% CI)  p-Value \| b (95% CI)  p-Value \| b (95% CI)  p-Value \| b (95% CI)  p-Value \| \| **Working from home (d/week)** \| -0.04  (-0.06,  -0.01) 0.003* \| -0.00  (-0.02, 0.02) 0.862 \| 0.16 (0.08, 0.24) 0.000 * \| 0.05  (-0.00, 0.10) 0.059 \| 0.04  (-0.01, 0.09) 0.115 \| 0.00  (-0.03, 0.04) 0.889 \| 0.23 (0.11, 0.36) 0.000 * \|   b: unstandardized regression coefficients; CI: confidence interval. All models were adjusted for age, sex, marital status, highest education, gross annual household income, changes in working days, and baseline sedentary behaviours. *p<0.05.   - An increase in working from home days was significantly associated with increases in work-related sitting time and total sitting time. However, an increase in working from home days was also associated with a decrease in car sitting time. - No significant associations were identified between changes in work style and changes in public transportation sitting, TV viewing time, PC use sitting, or other leisure sitting time.   Associations between absolute changes in working from home days and changes in workers’ domain-specific physical activities (complete data of 1315 individuals) (based on table 4).   \|  \| **Work-related Vigorous Physical Activity (h/d)** \| **Work-related Moderate Physical Activity (h/d)** \| **Transport-Related Physical Activity (h/d)** \| **Leisure Vigorous Physical Activity (h/d)** \| **Leisure Moderate Physical Activity (h/d)** \| **Total**  **Physical Activity (h/d)** \| \| --- \| --- \| --- \| --- \| --- \| --- \| --- \| \|  \| b (95% CI)  p-Value \| b (95% CI)  p-Value \| b (95% CI)  p-Value \| b (95% CI)  p-Value \| b (95% CI)  p-Value \| b (95% CI)  p-Value \| \| **Working from home (d/week)** \| 0.02 (-0.01, 0.05) 0.147 \| -0.06 (-0.10, -0.02) 0.004 * \| 0.02 (-0.01, 0.04) 0.124 \| 0.01 (-0.01, 0.02) 0.440 \| -0.00 (-0.02, 0.02) 0.897 \| -0.04 (-0.11, 0.03) 0.256 \|   b: unstandardized regression coefficients; CI: confidence interval. All models were adjusted for age, sex, marital status, highest education, gross annual household income, changes in working days, and baseline physical activities. *p<0.05.   - An increase in working from home days was associated with a decrease in work-related moderate physical activity.   There were no significant associations between the change in working from home days and changes in work-related vigorous physical activity, transport-related physical activity, leisure vigorous physical activity, leisure moderate physical activity, or total physical activity. | Funding:  T.N. was supported by the JSPS KAKENHI (#20H00040). K.O. is supported by a Grant-in-  Aid for Scientific Research (No. 20H04113) from the Japan Society for the Promotion of Science.  Conflict of Interest:  The authors declare no conflict of interest.  Overall RoB:  high (-) |
| **Koyama et al.**  2021  Japan  no study name  (Japan COVID-19 and Society Internet Survey (JACSIS))  08/04-25/05/2020 | Statistical methods:  inverse probability weighting adjusted estimates (rather than simple Internet survey estimates) are presented as the main results (to correct for selectivity)  intergroup comparisons using chi-squared tests (categorical variables)  logistic regression analyses to calculate the odds ratios (ORs) and 95% confidence intervals (CI) for prolonged sedentary time  two-way analysis of variance (to clarify the interaction between job types and start working from home on prolonged sedentary time ≥2 hours)  statistical significance was set at p <.05  Confounder:  multivariate-adjusted model included the following covariates: sex, age, prefecture, household income, education, chronic conditions, and working hours | Participant characteristics according to sedentary time during and after the state of emergency in 2020 (based on Table 1).   \| **Starting to work from home during the state of emergency** \| **sedentary time (h/d) during the state of emergency**  **Mean (SD)** \| **sedentary time (h/d) after the state of emergency**  **Mean (SD)** \| \| --- \| --- \| --- \| \| no \| 3.73 (3.15) \| 3.83 (3.20) \| \| yes \| 5.62 (3.37) \| 5.36 (3.39) \|  - Those who started work from home during the state of emergency had the longest extended sedentary time, with an average extended sedentary time of 16 minutes.   Distributions of prolonged sedentary times during and after the state of emergency in 2020 (based on Table 2).   \| **Starting to work from home during the state of emergency** \| **Prolonged sedentary time <2h**  **weighted n* (%)** \| **Prolonged sedentary time ≥2h**  **weighted n (%)** \| \| --- \| --- \| --- \| \| no \| 9371 (93.8) \| 622 (6.2) \| \| yes \| 1421 (87.2) \| 209 (12.8) \|   *weighted to be closer to a nationally representative sample of Japan   - Regarding work environment factors, prolonged sedentary time was reported in 12.8% of those respondents who started work from home during the state of emergency, including 9.7% of salespersons, and 7.7% of desk workers. - Those who started to work from home during the state of emergency tended to belong to the group with prolonged sedentary time >2hours (Table 2).   The multivariate-adjusted logistic analysis for the association of work environment factors with the prolonged sedentary time adjusted for sex, age, prefecture, household income, education, chronic conditions and working hours (Table 3)   \|  \|  \| **multivariate OR** \| **95% CI** \| \| --- \| --- \| --- \| --- \| \| **Job type** \| blue-collar worker \| Reference \|  \| \| desk worker \| 1.56 \| 1.27-1.91 \| \| salesperson \| 2.03 \| 2.64-2.51 \| \| **Starting to work from home during the state of emergency** \| no \| Reference \|  \| \| yes \| 2.14 \| 1.78-2.57 \|  - The adjusted OR of prolonged sedentary time was significantly higher in those who started work from home than in those who did not start work from home (OR: 2.14, CI: 1.78-2.57). (Table 3). - The adjusted OR of prolonged sedentary time was significantly higher in desk workers (OR: 1.56, CI: 1.27-1.91) and sales persons (OR: 2.03, CI: 1.64-2.51) than in blue-collar workers. - The interaction between job types and start working from home had a significant effect on prolonged sedentary time ≥2 hours (*P=*.002) (Table 3).   Adjusted ORs (95% CIs) of prolonged sedentary time by the combined effect of starting working from home and job type. (Figure 1)   \|  \| **not starting working from home OR (95% CI)** \| **starting working from home OR (95% CI)** \| \| --- \| --- \| --- \| \| **blue-collar worker** \| 1.00 (reference) \| 2.02 (1.05-3.91) \| \| **desk worker** \| 1.61 (1.30-2.00) \| 3.05 (2.33-4.01) \| \| **salesperson** \| 1.91 (1.52-2.41) \| 5.15 (3.75-7.07) \|   Compared with blue-collar workers who did not start working from home (the reference group), blue-collar workers, desk workers, and salespersons who started working from home had 2.0-fold, 3.1-fold, and 5.2-fold higher ORs of prolonged sedentary time. | Funding:  Research Support Program to Apply the Wisdom of the University to tackle COVID-19 Related Emergency Problems, University of Tsukuba, and Health Labour Sciences Research Grant, Grant/Award Number: 19FA1005 and 19FG2001; Japan Society for the Promotion of Science, Grant/Award Number: 17H03589, 18H03062, 18H03107, 19K10446 and  19K10671; JSPS Grant-in-Aid for Young Scientists, Grant/Award Number: 19K19439  Conflicts of Interest:  The authors declare no conflict of interest for this article.  Overall RoB:  low (+) |
| **Leskinen et al.**  2025  Finland  No study name  (Special Turku Coronary Risk Factor Intervention Projekt (STRIP))  05/2021-05/2022 | Statistical methods**:**  generalized linear  models (inter-individual difference) and linear mixed models (intra-individual difference days working from home and days working at the office of hybrid workers)  Confounder: sex, education, employment status, marital  status, perceived health, shift work and accelerometer wear time (inter-individuale difference); accelerometer wear time (intra-individual difference) | Daily physical activity and sedentary time for workdays by the work mode groups of non-manual workers (based on table 2)   \|  \| **In-office worker** \| **Hybrid workers** \| **Remote worker** \| \| --- \| --- \| --- \| --- \| \| **PA** \| Mean minutes (95% CI) \| Mean minutes (95% CI) \| Mean minutes (95% CI) \| \| Workday’s total PA \| 387 (330-444) \| 347 (290-405)* \| 324 (259-389)* \| \| Workday’s occupational PA \| 412 (349-475) \| 346 (283-409)* \| 330 (259-400)* \| \| Workday’s non-occupational PA \| 193 (137-249) \| 137 (81-194)* \| 143 (79-207)* \| \| **SB** \|  \|  \|  \| \| Workday’s total SED \| 584 (521-646) \| 650 (588-712)* \| 666 (596-712)* \| \| Workday’s occupational SED \| 288 (232-343) \| 343 (287-399)* \| 337 (273-401)* \| \| Workday’s non-occupational SED \| 372 (330-415) \| 387 (344-429) \| 400 (351-449) \| \| All model adjusted for sex, education, employment status, marital status, perceived health, shift work and accelerometer wear time  *significant difference compared with in-office worker \| \| \| \|   Daily total, occupational and non-occupational physical activity and sedentary time for office and remote workday among hybrid workers (based on table 3)   \|  \| Office workday \| Remote workday \| Difference \| \| --- \| --- \| --- \| --- \| \| Worktime \|  \|  \|  \| \| PA \|  \|  \|  \| \| Daily total PA \| 389 (355 to 424) \| 362 (328 to 397) \| -27 (-54 to 1) \| \| Occupational PA \| 160 (138 to 182) \| 135 (113 to 157) \| -26 (-53 to 2) \| \| Worktime activity bout length \| 3.4 (2.7 to 4.1) \| 2.6 (1.9 to 3.3) \| -0.8 (-1.9 to 0.2) \| \| Worktime activity bout number (n)* \| 51.9 (46.9 to 57.0) \| 51.7 (46.6 to 56.8) \| -0.2 (-5.2 to 4.8) \| \| Worktime PA fragmentation index \| 0.36 (0.32 to 0.40) \| 0.42 (0.38 to 0.46) \| 0.076 (0.002 to 0.11) \| \| Non-occupational PA \| 245 (219 to 271) \| 247 (221 to 273) \| 2 (-19 to 23) \| \| Sedentary time (SED) \|  \|  \|  \| \| Daily total SED \| 594 (560 to 629) \| 624 (589 to 658) \| 30 (-0.4 to 59) \| \| Occupational SED \| 315 (293 to 337) \| 341 (219 to 362) \| 26 (-19 to 23) \| \| Worktime sedentary bout length \| 7.0 (5.4 to 8.5) \| 7.4 (5.9 to 9.0) \| 0.4 (-0.9 to 1.8) \| \| Worktime SSE fragmentation index \| 0.19 (0.16 to 0.22) \| 0.16 (0.13 to 0.19) \| -0.03 (0.08 to 0.008) \| \| Worktime long sedentary bout sum \| 84 (57 to 110) \| 85 (59 to 111) \| 2 (-29 to 32) \| \| Non-occupational SED \| 323 (297 to 349) \| 321 (295 to 347) \| -2 (-29 to 19) \|   **Interindividual differences:**   - […], the in-office worker accumulated 1 hour less occupational sedentary time compared with the hybrid and remote workers. - Workdays’ PA and SB did not differ between the hybrid and remote workers - Compared with in in-office workers, who commute and work at the office every workday, both hybrid and remote workers accumulated less PA during their workdays and did not reach the activity level of in-office workers until the late evening hours   **Intraindividual differences:**   - Compared with the office days, the remote workday accumulated less PA and more sedentary time, especially during worktime, although the difference only approached significance. - The occupational PA tended to be lower for remote compared with office workdays (mean difference -26 min, 95% Ci -53 to 2, p=0.06), with s significantly higher activity fragmentation, that is the probability for transitioning from active to sedentary state during the remote working hours (42% vs. 36%, p=0.04) | Funding:  Financial support was received from the Academy of Finland (grant  numbers: 206374, 294834, 251360, 275595, 307996, 322112, 332030, 361780);  the Juho Vainio Foundation; the Finnish Foundation for Cardiovascular Research; the Finnish Ministry of Education and Culture; the Finnish Cultural Foundation; the Sigrid Jusélius Foundation; State Research Funding; the Yrjö Jahnsson Foundation; the Finnish Medical Foundation; and the Turku University Foundation.  Conflict of Interest:  The authors declare no conflict of interest.  Overall RoB:  almost low (+/-) |
| **Loef et al. (1)**  2022a  The Netherlands  no study name  (Lifelines population cohort/ Lifelines COVID-19 cohort)  03/2020-02/2021 | Statistical methods:  independent-samples t-test and the chi-square test were used (to gain insight into differences in the characteristics of the study population by overall work situation (location/ home/hybrid workers) during the COVID-19 pandemic  p-values <0.05 were considered statistically significant  logistic generalized estimating equations (GEE) analysis with an exchangeable correlation structure was used to study the longitudinal association between work situation and PA and work situation and SB  Confounder:  age, sex, educational level, country of birth, household composition, occupation, occupational class, employment contract, general health, and testing positive for COVID-19, moderate-to-vigorous-intensity activity/SB before the COVID-19 pandemic | Adjusted effect estimates between work situation and PA (n=33,325). Reference group=location workers [CI=confidence interval; OR=odds ratio]. (based on Table 2)   \| **PA outcome measures** \| **Home workers** \| \| **Hybrid workers** \| \| \| --- \| --- \| --- \| --- \| --- \| \| OR \| 95% CI \| OR \| 95% CI \| \| Current moderate-to-vigorous-intensity activity during pandemic (≥150 minutes vs. <150 minutes per week) \| 0.93 \| 0.90-0.96 \| 1.02 \| 0.98-1.07 \|   p<0.05   - Home workers were less likely to perform ≥150 minutes/week of moderate-to-vigorous-intensity activity during the pandemic than location workers [odds ratio (OR) 0.93, 95% confidence interval (CI) 0.90–0.96]; no differences were observed between hybrid and location workers.   Adjusted effect estimates between work situation and SB (N=18,379). Reference group=location workers (based on Table 3).   \| **SB outcome measures** \| **Home workers** \| \| **Hybrid workers** \| \| \| --- \| --- \| --- \| --- \| --- \| \| OR \| 95% CI \| OR \| 95% CI \| \| Current SB on workdays during the pandemic (sitting ≥8 hours vs. <8 hours per day) \| 1.94 \| 1.83–2.06 \| 1.73 \| 1.59-1.88 \|   P<0.05   - Home workers had 1.94 (95% CI 1.83–2.06) times more odds to sit ≥8 hours on workdays during the pandemic than location workers after adjustment for covariates. - Hybrid workers had 1.73 (95% CI 1.59–1.88) times more odds to sit ≥8 hours on workdays during the pandemic than location workers after adjustment for covariates. | Funding:  funded by the COVID-19 program of the Dutch Ministry of Health, Welfare and Sport that was conducted by the Dutch National Institute for Public Health and the Environment  Conflicts of Interest:  The authors declare no conflicts of interest.  Overall RoB:  almost low (+/-) |
| **Loef et al. (2)**  2022b  The Netherlands  no study name  (Lifelines population cohort/ Lifelines COVID-19 cohort)  03/2020-01/2022 | Statistical methods:  structural equation modelling (SEM) was used to estimate the different paths of the mediation models  multilevel SEM was conducted with logistic regression adjusting for the dependency of observations within an individual over time  Confounder:  age, sex, education level, country of birth, household composition, occupation and occupational class, employment contract, general health, PA before the pandemic, and SB before the pandemic | - Home workers (OR=0.87, 95% CI=0.83–0.91), but not hybrid workers (OR=1.04, 95%-CI=0.98–1.10), were less likely to be physically active for ≥150min per week than location workers in the total study population (see study results page 07). - Home workers (OR=2.82, 95%-CI=2.56–3.09; n=14,333) and hybrid workers (OR=2.44, 95%-CI=2.18–2.74; n=14,333) were more likely to be sedentary for >9 h per workday than locations workers (see study results page 07). | Funding:  funded by the COVID-19 program of the Dutch Ministry of Health, Welfare and Sport that was conducted by the Dutch National Institute for Public Health and the Environment.  Conflicts of Interest:  The authors declare that they have no conflicts of interest.  Overall RoB:  almost low (+/-) |
| **Marenus et al.**  2025  USA  No study name  (no underlying cohort)  Not reported | Statistical method:  Descriptive analysis, A multivariate analysis of variance (MANOVA) was performed to examine differences in outcome variables among three workplace groups. ANOVAs were run to examine the effect of remote work with Bonferroni correction.  Confounder:  Age, gender, industry, job class, race/ethnicity, income level, education status | Average scores of PA in MET-min per week by remote work status (based on table 2)   \|  \| **Onsite** \| **Hybrid** \| **Remote** \| \| --- \| --- \| --- \| --- \| \| **PA** \|  \|  \|  \| \| Vigorous PA (SD) \| 954 (1601) \| 934 (1130) \| 852 (1438) \| \| Moderate PA (SD) \| 631 (991) \| 413 (478) \| 426 (840) \| \| Walking (SD) \| 903 (1061) \| 622 (642) \| 536 (614) \| \| Total PA (SD) \| 2489 (2949) \| 1970 (1700) \| 1792 (2233) \|   Results of ANCOVAS analyzing differences in PA by workplace status (onsite, hybrid, or remote) (based on table 3)   \|  \| **F** \| **Partial η2** \| **p-value** \| \| --- \| --- \| --- \| --- \| \| **PA** \|  \|  \|  \| \| Vigorous PA (SD) \| 0.23 \| 0.001 \| 0.793 \| \| Moderate PA (SD) \| 4.33 \| 0.020 \| 0.020 \| \| Walking (SD) \| 9.19 \| 0.040 \| 0.001 \| \| Total PA (SD) \| 4.11 \| 0.020 \| 0.017 \| \| Note: Models controlled for age, gender, income, race, ethnicity, education status, and tenure at workplace \| \| \| \|   Results of post hoc tests for significant ANCOVAs (based on table 4)   \|  \| **t** \| **p-value** \| \| --- \| --- \| --- \| \| **Walking** \|  \|  \| \| Hybrid – Onsite \| -3.15 \| 0.005 \| \| Hybrid – Remote \| 0.68 \| 0.675 \| \| Onsite – Remote \| 3.69 \| 0.000 \| \| **Total PA** \|  \|  \| \| Hybrid – Onsite \| -2.19 \| 0.005 \| \| Hybrid – Remote \| 0.41 \| 0.910 \| \| Onsite – Remote \| 2.50 \| 0.034 \|  - There are significant differences in walking (F=9.58, p<0.001) and in total PA (4.19, p=0.016) by workplace - There were no differences in vigorous PA or moderate PA by workplace status. - Post hoc test revealed that onsite employees had significantly higher levels of walking than hybrid (t=-3.14, p=0.005) and remote employees (t=3.68, p<0.001). - Post hoc test also revealed that onsite employees had higher levels of total PA than hybrid (t=-2.31, p=0.005) and remote employees (t=2.50, p= 0.034) - There was no [significant] difference in walking or total PA between hybrid and remote employees. | Funding:  Funding Sources: This work was supported by the University of Michigan Rackham Graduate Student Research Grant. N.C. received consultancy fees from an NIH grant from University of South Carolina.  Conflict of Interest:  The authors have declared no competing interest.  Overall RoB:  high (-) |
| **Massar et al.**  2022  Singapore  no study name  (Health Insights Singapore (hiSG))  08/2021-01/2022 | Statistical methods:  linear mixed-effects models were used to evaluate the effect of work location and study phase […] on outcome measures. Work location and study phase (1–3) were modelled as fixed effects, while participant ID was modelled as a random effect […].  Confounder:  Considered in a control analysis: age, gender, education, family status (having children vs. no children), intervention group (Control vs. intervention). For each demographic/group variable a main effects and its interaction with work arrangement were included in the models. | For daily step count, a significant main effect of Work arrangement was found (F=31.00, p<0.001). Paired comparisons indicated that both WFO day and No-work days were associated with significantly more daily steps (~10,000) than WFH (~7,000). This effect was present across all study phases, as was evident from the absence of a Phase main effect (p=0.10) or a Work arrangement x Phase interaction (p=0.96).  [Results of the control analysis showed] for all objective sleep and activity metrics, the main effect of Work arrangements remained significant (all F’s>8.26, p’s<0.0003). However, the effects of work arrangement were not significantly altered by inclusion of  demographics in the models.  Corresponding data of figure 3 (personal communication)   \|  \| **WAO** \| \| **WFH** \| \| **No work** \| \| \| --- \| --- \| --- \| --- \| --- \| --- \| --- \| \| **Steps** \| Mean \| SEM \| Mean \| SEM \| Mean \| SEM \| \| **Phase 1 Aug 2021** \| 9778 \| 380 \| 7215 \| 366 \| 9972 \| 346 \| \| **Phase 2**  **Oct - Nov 2021** \| 9526 \| 443 \| 7159 \| 374 \| 9613 \| 351 \| \| **Phase 3**  **Jan 2022** \| 10374 \| 389 \| 7891 \| 386 \| 10374 \| 356 \| | Funding:  The hiSG study was developed and supported by the Health Promotion Board, Singapore. Personnel for the data analysis were additionally supported by grants from the National Medical Research Council, Singapore (NMRC/STaR/015/2013 and STaR19May-001), funds for the Centre for Sleep and Cognition, Yong Loo Lin School of Medicine, and the Lee Foundation awarded to Prof. Michael Chee.  Conflicts of Interest:  The authors declare that the research was conducted in the absence of any commercial or financial relationships that could be construed as a potential conflict of interest.  Overall RoB:  high (-) |
| **Matthews et al.**  2022  USA  no study name  (AmeriSpeak)  16/10-11/11/2019 and 03/11/-15/11/2020 | Statistical methods:  calculation of mean of each time variable  Confounder  not considered | Daily behaviour 2019 and 2020 by occupational and work from home status; presented as mean (based on table 4)   \| **Daily behaviours** \| **Work from home**  **(n=400)** \| \| **Not working from home**  **(n=697)** \| \| \| --- \| --- \| --- \| --- \| --- \| \|  \| **2019** \| **2020** \| **2019** \| **2020** \| \| Sedentary time(h/d) \| 10.96 \| 10,6 \| 9.13 \| 9 \| \| Total PA (h/d) \| 5.09 \| 5,1 \| 6.99 \| 6,9 \| \| Light PA (h/d) \| 3.71 \| 3,5 \| 3.86 \| 3,9 \| \| Moderate-vigorous PA (h/d) \| 1.39 \| 1,6 \| 3.13 \| 3,0 \|  - Working from home is associated with longer sedentary time and less total PA. - Sedentary time during work from home seems to be longer compared to not working from home. | Funding:  This research was supported by the NIH/NCI Intramural Research Program (Z99 CA999999).  Conflicts of Interest:  The authors have declared that no competing interests exist.  Overall RoB  high (-) |
| **Moura et al.**  2022  Brazil  COVID Inconfidentes  (no underlying cohort)  10/2020-12/2020 | Statistical methods:  multivariate logistic regression  Confounder:  age and sex | Association of physical inactivity (PI) during two moments (M1 and M2) of the COVID-19 pandemic-effect of work routine during social restriction (based on Table 3)   \|  \| **PI (M1)**  **(March to August 2020)** \| \| **PI (M2)**  **(October to December 2020)** \| \| \| --- \| --- \| --- \| --- \| --- \| \|  \| OR (95% CI) \| p \| OR (95% CI) \| p \| \| **No work from home** \| 1.00 (Ref) \|  \| 1.00 (Ref) \|  \| \| **No work** \| 1.29 (0.77–2.20) \| 0.325 \| 0.97 (0.62–1.51) \| 0.880 \| \| **Work from home** \| **0.52** (0.30–0.89) \| 0.019 \| **0.49** (0.28–0.84) \| 0.010 \|   Adjusted for sex and age  Individuals working from home were less likely to be physically inactive (M1: OR=0.52; 95% CI: 0.30–0.89; M2: OR=0.49; 95% CI: 0.28–0.84), considering the individuals in normal work routine as reference. | Funding:  Federal University of Ouro Preto (UFOP)  [PROPPI/UFOP no18/2022], Brazilian Council for Scientific and Technological Development (CNPq).  Conflicts of Interest:  The authors declare that they have no competing interests.  Overall RoB:  high (-) |
| **Moura et al.**  2023  Brazil  COVID Inconfidentes  (no underlying cohort)  10/2020-12/2020 | Statistical methods:  univariate and multivariate logistic regression  Confounder:  sex, age, family income, symptoms of depression or anxiety, BMI, work, and work schedule | Univariate Regression Between Physically Active and Inactive Individuals (based on table 1)   \| **Variable** \| **Total (row%)** \| **Physically Active in col% (95% CI)** \| **Physically Inactive in col% (95% CI)** \| **OR (95% CI)**  **for PI** \| \| --- \| --- \| --- \| --- \| --- \| \| **Work routine** \|  \|  \|  \|  \| \| Not WFH \| 79.8 (75.2-83.7) \| 27.1 (22.2-32.6) \| 72.9 (67.4-77.8) \| 1.00 \| \| Partial WFH \| 9.0 (7.1-12.2) \| 50.7 (37.1-64.2) \| 49.3 (35.8-62.9) \| **0.36 (0.19-0.68)** \| \| Full WFH \| 11.2 (8.7-14.4) \| 42.7 ( \| 57.3 (46.9-67.2) \| **0.50 (0.30-0.83)** \|   Data in bold represent statistically significant differences.  N=1,750, 95% CI, 95% confidence interval, OR, odds ratio, WFH, work from home   - Individuals who worked partially or fully WFH were less likely to be physically inactive PI - Individuals who reported no WFH had a 1.86 times greater chance of PI at leisure (OR=1.86; 95% CI: 1.08-3.23; b=0.62; 95% CI: 0.08-1.17) compared with those who reported full or partial WFH (adjusted analysis) | Funding:  This study was supported by the Federal University of Ouro Preto (UFOP, PROPPI/UFOP n°03/2023), Brazilian Council for Scientific and Technological Development (CNPq), Coordination for the Improvement of Higher Education Personnel-Brazil (CAPES, 9/2020; n°88887.504994/2020e00), Foundation for Research Support of the State of Minas Gerais (FAPEMIG, n°001/2021; APQ 02445–21), and finance code 001 for PhD student scholarship.  Conflicts of Interest:  None declared.  Overall RoB:  high (-) |
| **Olsen et al.**  2018  Australia  No study name  (no underlying cohort)  time of data collection is not reported | Statistical methods:  overall time in PA was compared with national recommendations (at least 150 min/week) to determine the proportion of individuals meeting guidelines  Wilcoxon signed rank test was used to assess change in self-reported weighted time spent in PA, and in self-reported total time spent sitting, from before to after the policy implementation  Confounder:  not applicable due to within subject comparison | Self-Reported Time Spent in Physical Activity (min/week) N=24 (based on Table 2)   \|  \| **Pre (4 weeks before implementation)** \| **Post (6 weeks after implementation)** \|  \| \| --- \| --- \| --- \| --- \| \|  \| Median (IQR*) \| Median (IQR) \| ΔP** \| \| **Weighted moderate PA (min/week)** \| 375 (131–750) \| 330 (110–757) \| 0.770 \|   *interquartile range  **Wilcoxon signed rank test   - Majority of participants did meet the guideline of at least 150 weighted minutes of PA per week at both time points (pre=81%, post=71%). - There was no significant difference in weighted moderate PA between pre- and post-assessments, Z=-0.29, P> 0.05, indicating that PA does not change after implementation of flexible work policy).   Self-Reported Time Spent Sitting (min/d) on a Usual Workday at Home and Usual Workday at Office N=24 (based on Table 3)   \|  \| **Sitting on a Usual Day When Working at Office** \| \| \| **Sitting on a Usual Day When Working at Home** \| \| \| \| --- \| --- \| --- \| --- \| --- \| --- \| --- \| \|  \| Pre \| Post \|  \| Pre \| Post \|  \| \|  \| Median (IQR) \| Median (IQR) \| ΔP \| Median (IQR) \| Median (IQR) \| ΔP \| \| **Work** \| 420 (377–480) \| 450 (420–480) \| 0.158 \| 30 (0–442.5) \| 450 (0–480) \| 0.358 \| \| **Total time spent sitting** \| 646.5 (576–721) \| 705 (630–863) \| 0.007 \| 60 (0–644.5) \| 641 (510–847.5) \| 0.042 \|  - Overall sitting time on a usual workday at home increased after the intervention (pre-Median=60 minutes, post Median=641 minutes) which demonstrates that individuals had commenced working from home in line with the flexible work policy. - The domain with the highest time spent sitting (post) was ‘‘work’’ on both a usual workday at home and usual workday at the office. - There were significant increases in total sitting time on a usual workday at the office, Z=-4.16, P>0.001 and on a usual workday at home, Z=2.02, P>0.05. | Funding:  none reported  Conflicts of Interest:  The authors declare no conflicts of interest.  Overall RoB:  high (-) |
| **Oxenham et al.**  2025  United Kingdom  No study name  (UK Household Longitudinal Study (UKHLS)  2015-2017 (wave 7) -  2022-2023 (wave 13) | Statistical method:  Multilevel linear models with robust standard errors with interaction terms  Confounder:  Sex, ethnicity, parental education status, NS-SEC of their first job and own education status | Changes in MET-min per day over time before and after starting work, showing adjusted interactions with different individual and job characteristic. Transition tin work shows change in intercept immediately after starting work and change in slope after starting work shows longer-term trends in MET-min per day each year after starting work   \|  \|  \| Transitions in work [95%CI] \| Change in slope after starting work [95% CI] \| \| --- \| --- \| --- \| --- \| \| Work location \| Office \| 128.81 [89.46, 168.16] \| -27.39 [-44.58, -10.19] \| \| Home \| -126.42 [-264.45, 11.61]] \| 39.85 [-13.89, 93.59] \| \| Interaction p-value \| <0.001 \| 0.019 \|   Working from home was associated with an initial decrease in physical activity (=-126.42, [-264.45 to 11.61] MET-min/day), whereas those who worked in an office/employer’s premises showed an initial increase (ß=128.81[89.46 to 168.16] MET-min/day) although this difference was not maintained. | Funding:  This work was funded by the NIHR [Work and Health Research Programme, grant number NIHR206285]. The views expressed are those of the authors and not necessarily those of the NIHR or the Department of Health and Social Care. EMW is funded by a Career Development Award from the UK Medical Research Council [grant number MR/T010576/1]. TB was funded by the Cambridge MRC Doctoral Training Partnership [grant number MR/N013433/1] and the Elizabeth McDowell Studentship at Newnham College, Cambridge. The contribution of EvS was supported by the Medical Research Council [grant number MC_UU_00006/5].  Conflict of Interest:  The authors have declared no competing interest.  Overall RoB:  high (-) |
| **Prince et al.**  2024  Canada  No study name  (Canadian Community Health Survey (CCHS))  06/2021-02/2022 | Statistical method:  Descriptive statistics including means and 95% CI, Chi-square and ANOVA, multivariate logistic regression; statistically significant results were identified when p-values  were <0.01 for categorical (to adjust for multiple comparisons)  and 0.05 for continuous variables, and when the 95% CIs did not include  zero or one, depending on the estimate.    Confounder: age group, gender, household education, racial/cultural background, BMI category, multimorbidity, self-rated general health, self-rated mental health, COVID-19 vaccinations status, work stress and work hours | PA in minutes per week by work location, working adults aged 18 to 75 years (based on supplementary table 2)   \|  \| **Fixed workplace**  **% (95 CI)** \| **Telework**  **% (95 CI)** \| **Non-fixed workplace**  **% (95 CI)** \| **Between- groups p-value** \| \| --- \| --- \| --- \| --- \| --- \| \| MVPA-Recommendations (all) (%) \| 60.7 (58.7, 62.7) \| 65.2 (62.5, 68.0) \| 69.9 (65.8, 73.9) \| 0.0002† \| \| MVPA-Recommendations (recreation and transportation domains) \| 41.9 (39.8, 44.0) \| 52.0 (48.9, 55.0) \| 44.0 (39.5, 48.5) \| <.0001*‡ \| \| Mean total PA \| 472.4 (437.4, 507.4) \| 341.1 (318.5, 363.7) \| 720.8 (647.1, 794.6) \| 0.0032† \| \| Median total PA \| 209.8 (188.7, 230.9) \| 238.1 (216.5, 259.7) \| 411.5 (344.7, 478.2) \| \| Mean transportation PA \| 17.7 (14.3, 21.0) \| 14.6 (13.0, 16.3) \| 22.1 (16.4, 27.7) \| 0.0153* \| \| Mean recreational \| 17.0 (15.7, 18.2) \| 21.1 (19.1, 23.1) \| 18.2 (15.1, 21.2) \| <.0001*‡ \| \| Mean occupational and household \| 32.8 (29.5, 36.2) \| 13.0 (11.3, 14.7) \| 62.7 (53.5, 72.0) \| <.0001*†‡ \| \| No physical activity – all domains (%) \| 16.9 (15.3, 18.5) \| 11.8 (10.0, 13.5) \| 17.6 (14.1, 21.2) \| 0.0002*‡ \| \| *Fixed workplace significantly different from telework. †Fixed workplace significantly different than non-fixed workplace. ‡Telework significantly different from non-fixed workplace. \| \| \| \| \|   Compared to working outside of the home at either a fixed or non-fixed location telework had the lowest proportion reporting no PA (telework: 11.8%, fixed: 16.9%, non-fixed: 17.6%), the highest volumes of recreational PA (telework: 21.1, fixed: 17.0, non-fixed: 18.2 min/day) […]  Unadjusted and adjusted associations between work location and meeting the individual recommendations, working adults aged 18-75 years (based on table 2)   \|  \| Unadjusted OR (95% CI) \| Adjusted OR^a^ (95% CI) \| \| --- \| --- \| --- \| \| **MVPA-recommendation (all) – meeting recommendation** \|  \|  \| \| Fixed workplace \| Ref. \| Ref. \| \| Telework \| 1.22 (1.05, 1.41) \| 1.19 (1.00, 1.41) \| \| Non-fixed-workplace \| 1.50 (1.21, 1.86) \| 1.46 (1.15, 1.85) \| \| **MVPA-recommendation (AT+Rec) – meeting recommendation** \|  \|  \| \| Fixed workplace \| Ref. \| Ref. \| \| Telework \| 1.50 (1.29, 1.74) \| 1.36 (1.15, 1.61) \| \| Non-fixed-workplace \| 1.09 (0.89, 1.34) \| 1.27 (1.01, 1.59) \|   Compared to a fixed workplace, teleworkers were significantly more likely to meet the MVPA-recommendation (AT+Rec) (adjusted odds ratio=1.36, 95% CI: 1.15-1.61) | Funding:  No external funding was received for this work.  Conflict of Interest:  The authors declare that they have no conflicts of interest to disclose.  Overall RoB:  high (-) |
| **Sauter et al.**  2025  Germany  SITFLEX-1  (no underlying cohort)  07-11/2021 | Statistical methods:  linear mixed models (LMM) estimated  with restricted maximum likelihood and random  intercepts for study participants and interaction term (office x workplace)  Confounders:  Gender, age, application engineer and measurement phase | Estimated marginal means for PA and SB variables by workplace (based on own data)   \|  \| **WFH** \| **WAO** \| \| --- \| --- \| --- \| \| **Work time** \|  \|  \| \| SB in min/work (95% CI) \| 360 (323-396) \| 314 (278-350) \| \| STS in counts/work (95% CI) \| 28.9 (24.7-33.2) \| 25.4 (21.2-29.6) \| \| Steps in counts/work (95% CI) \| 2027 (1616–2439) \| 3515 (3105-3925) \| \| **Total time awake** \|  \|  \| \| SB in min/day (95% CI) \| 637 (585-688) \| 605 (554-657) \| \| STS in counts/day (95% CI) \| 57.9 (50.8-65.0) \| 50.9 (43.9-58.0) \| \| Steps in counts/day (95% CI) \| 7434 (6187-8681) \| 9454 (8203-10705) \| \| Adjusted for age, gender, application engineer and measurement phase  STS – sit-to-stand transitions \| \| \|  - When WFH time spent sedentary increased compared to WAO in hybrid office workers during work time and during total time awake, this result is in accordance with results of the compositional data analysis. - When WFH count of steps decreased compared to WAO in hybrid office workers during work time and during total time awake.   When WFH STS increased compared to WAO in hybrid office workers during work time and total time awake. | Funding:  The study is part of the F2499 project conducted by the Federal Institute for Occupational Safety and Health (BAuA). The funding body (BAuA) played no role in the design of the study and collection, analysis, and interpretation of data and in writing the manuscript.  Conflict of Interest:  The authors declare no conflicts of interest.  Overall RoB:  almost low (+/-) |
| **Scurati et al.**  2024  Italy  no study name  (no underlying cohort)  01- 05/2023*  *personal communication | Statistical methods:  Unpaired t-test (or respectively Mann-Whitney U Test) compared the physical status […] of hybrid and on-site workers and Person’s correlation (or respective Spearman’s rho) between the percentage of remote work per week and each variable was calculated solely for hybrid workers  Confounders:  Not considered | Physical status outcomes comparison between hybrid and on-site workers (based on table II)   \| **Variables** \| **Hybrid workers** \| **On-site workers** \| \| --- \| --- \| --- \| \| **IPAQ** \|  \|  \| \| Vigorous activity (MET) \| 1216.4±1360.3 * \| 553.3±891.6 \| \| Moderate activity (MET) \| 612.0±828.6 \| 774.0±1044.3 \| \| Walking activity (MET) \| 957.0±1425.9 \| 995.3±1297.6 \| \| Sitting activity (min/day) \| 562.4±137.6 \| 528.3±119.5 \| \| Total activity (MET) \| 2785.4±2331.6 \| 2326.0±1783.5 \| \| **Accelerometer** \|  \|  \| \| Vigorous activity (min/week) \| 31.4±49.4 \| 19.7±46.8 \| \| Moderate activity (min/week) \| 940.8±346.8 \| 964.9±428.3 \| \| Light activity (min/week) \| 562.0±125.2 \| 520.2±123.6 \| \| Sedentary time (min/week) \| 8080.8±461.7 \| 8111.8±559.2 \| \| Total activity (min/week) \| 1534.2±446.4 \| 1504.8±544.9 \| \| Values are reported as mean±SD. MET values indicate MET-min per week.  SD: standard deviation; AU: arbitrary units; MET: metabolic equivalent of task.  *Different than on-site workers (P<0.05). \| \| \|  - Concerning weekly physical activity, the IPAQ results detected that hybrid workers, when doing physical activity, performed more than double of the vigorous activity of workers on-site. - Direct correlation was found between the percentage of remote work and vigorous activity as measured by the IPAQ (based on figure 1). | Funding:  This work was funded by the University of Milan (grant number PSR2021_SCURATI_RAFFAELE).  Conflict of Interest:  The authors certify that there is no conflict of interest with any financial organization regarding the material discussed in the manuscript.  Overall RoB:  high (-) |
| **Sers et al.**  (2023)  Germany (area of Karlsruhe)  No study name  (no underlying cohort)  time of data collection is not reported | Statistical methods:  descriptive and multi-level analyses (two-level model with days of assessment (level 1) nested within participants (level 2)  Confounder:  age (years), sex (male vs. female), body mass index (kg/m^2^), household size (number of cohabitants), and distance to work (km) as between person (level 2) and valence (0-100), sleep quality (0-100), sleep duration (h), social stress (1-7), activity stress (1-7), and work ability (1-10) as within-person (level1) variables | Descriptive statistics of PB parameters by work environment (based on table 2)   \|  \| **Days WAO (n=135)** \| **Days WFH (n=141)** \| \| --- \| --- \| --- \| \| **Variable** \|  \|  \| \| **Parameters of PA** \|  \|  \| \| LPA time (h/day) \| 2.21 +/- 1.15 \| 2.33 +/- 1.20 \| \| MVPA time (h/day) \| 1.18 +/- 0.85 \| 0.87 +/- 0.70 \| \| Physical activity intensity (MET) (per day) \| 1.5.4 +/- 0.20 \| 1.48 +/- 0.22 \| \| Steps (no/day) \| 7548 +/- 3944 \| 6299 +/- 3779 \| \| PA bouts short (no/day) \| 23.44 +/- 11.42 \| 25.82 +/- 11.37 \| \| PA bouts short-to-moderate (no/day) \| 8.64 +/- 3.63 \| 7.88 +/- 4.03 \| \| PA bouts moderate-to-long (no/day) \| 2.59 +/- 1.65 \| 1.99 +/- 1.66 \| \| PA bouts long (no/day) \| 1.35 +/- 1.35 \| 1.38 +/- 1.44 \| \| **Parameters of SB** \|  \|  \| \| SB time (h/day) \| 9.61 +/- 2.53 \| 9.92 +/- 2.55 \| \| SB breaks (no/day) \| 36.01 +/- 12.34 \| 37.06 +/- 11.49 \| \| SB bouts short (no/day) \| 16.30 +/- 9.35 \| 17.21 +/- 8.93 \| \| SB bouts short-to-moderate (no/day) \| 10.44 +/- 4.99 \| 10.53 +/- 4.38 \| \| SB bout moderate-to-long (no/day) \| 5.43 +/- 2.51 \| 5.22 +/- 2.52 \| \| SB bouts long (no/day) \| 3.70 +/- 1.78 \| 4.14 +/- 2.10 \|   Multilevel model analyses (based on table 3 and table 4)   \|  \| **WFH^a^** \| \| --- \| --- \| \| **Models of PA-related Outcomes** \| b (SE) \| \| LPA time \| 0.09 (0.15) \| \| MVPA time \| -0.30 (0.08)** \| \| MET \| -0.05 (0.02)* \| \| Steps \| -1288.1 (449.0)* \| \| Short PA-bouts \| 3.60 (1.32)* \| \| Short-to-moderate PA-bouts \| -0.87 (0.64) \| \| Moderate-to-long PA-bouts \| -0.62 (0.23)** \| \| Long PA-bouts \| 0.07 (0.16) \| \| **Models of SB-related Outcomes** \|  \| \| SB time \| 0.16 (0.37) \| \| SB breaks \| 1.78 (1.84) \| \| Short SB bouts \| 1.95 (1.09) \| \| Short to moderate SB bouts \| 0.05 (0.59) \| \| Moderate-to-long SB bouts \| -0.36 (0.35) \| \| Long SB bouts \| 0.36 (0.26) \|   note: Data were presented as unstandardized estimates (b) and standard errors (SE)  note: * p<0.05; **p<0.01.  ^a^compared to working at the office (WAO).   - WFH has a negative effect on MVPA time, steps, and physical activity intensity (MET), but a positive effect on short PA bouts (=5 min). - No associations between the work environment and any SB parameter (i.e., SB time, SB breaks, SB bouts) were found | Funding:  Open Access funding enabled and organized by Projekt DEAL.  Conflicts of Interest:  not reported  Overall RoB:  almost low (+/-) |
| **Silva et al.**  2021  ConVid - Behavior Survey  (no underlying cohort)  24/04-24/05/2020 | Statistical methods:  data were analysed using percentages and 95% confidence intervals (95% CI)  Confounder:  not considered | In Brazil the prevalence of physical inactivity (< 150 minutes/week PA) during the COVID-19 pandemic (2020) was 88.9 % (95% CI [86.5-90.9]) in those working in normal routine and 86.6 % (95% CI [84.6-88.4]) in those working from home (table 4;)  Before the pandemic prevalence of physical inactivity (< 150 minutes/week activity) was 70.6% (95% CI [67.3-73.6]) in those working normal routine and 69.2% (95% CI [66.7-71.1]) in those working from home. (based on table 4)   \|  \| **% Physically Inactive (95% CI**) \| \| \| --- \| --- \| --- \| \|  \| Before pandemic \| During pandemic \| \| **Normal work routine** \| 70.6 (67.3-73.6) \| 88.9 (86.5-90.9) \| \| **Home Office** \| 69.2 (66.7-71.6) \| 86.6 (84.6-88.4) \| | Funding:  A. O. Werneck is supported by the São Paulo State Research Foundation (FAPESP) with a PhD scholarship (FAPESP process: 2019/24124-7)  D. C. Malta (process: 308250/2017-6)  M. B. A. Barros (process: 303241/2019-5)  C. L. Szwarcwald (process:310318/2020-3) are supported by the Brazilian National Research Council (CNPq), which funded the productivity scholarship  Conflicts of Interest:  not reported  Overall RoB:  high (-) |
| **Suzuki et al.**  2025  Japan  No study name  (no underlying cohort)  04-05/2020 | **Statistical methods:**  Chi-squared and Fisher’s exact tests were used to compare categorical data  Statistical significance was set at P<0.05.  **Confounder:**  Not considered | The physical activity decreased significantly in 60.9% of the homegroup (p<0.001) compared to 29.7% in commuting group (based on figure 2B)**.** | Funding:  none  Conflicts of Interest:  The authors have no conflicts of interest to declare.  Overall RoB:  high (-) |
| **Thralls Butte et al.**  2023  No study name  (no underlying cohort)  time of data collection not reported | Statistical methods:  one way Analysis of Variance (ANOVA) was used to assess differences between work environment (office vs. home vs. hybrid) and ActivPAL™ behavior variables and PA variables  α-level of 0.05 was used for tests of statistical significance.  Confounder:  not considered | Means, SDs, F-Statistic, and p-value of self-report and objective activity variables by work environment (based on Table 2)   \| **Variable** \| **Home** \| **Office** \| **Mix** \| **Total** \| **F (df)** \| **p^ƚ^** \| \| --- \| --- \| --- \| --- \| --- \| --- \| --- \| \| **Self-report** \|  \|  \|  \|  \|  \|  \| \| Sitting (hrs/d) \| 6.6 (1.8) \| 6.5 (1.3) \| 6.6 (0.6) \| 6.6 (1.4) \| 0.07 (240) \| 0.94 \| \| Aerobic PA (min/d) \| 103 (51) \| 81 (69) \| 150 (136) \| 109 (88) \| 1.99 (2,39) \| 0.15 \| \| Muscle PA (min/d) \| 2.2 (2)* \| 0.5 (1.2)* \| 2.0 (1.9) \| 1.7 (1.9) \| 3.64 (2,40) \| *0.04 \| \| **ActivPAL** \|  \|  \|  \|  \|  \|  \| \| Sitting (min/d) \| 569 (111)* \| 477 (46)* \| 526 (93) \| 530 (93) \| 4.17 (2,37) \| *0.02 \| \| Standing (min/d) \| 208 (60) \| 259 (82) \| 216 (51) \| 225 (68) \| 2.24 (2,37) \| 0.12 \| \| Stepping (min/d) \| 87 (36) \| 78 (20) \| 88 (25) \| 85 (29) \| 0.42 (2,37) \| 0.66 \| \| Steps (#/d) \| 7289 (3317) \| 5984 (1556) \| 7766 (2883) \| 7016 (2811) \| 1.27 (2,37) \| 0.29 \| \| STS (#/d) \| 45 (16) \| 51 (17) \| 40 (9) \| 45 (15) \| 1.54 (2,37) \| 0.23 \|   SD standard deviation, PA Physical Activity  ^ƚ^p-value from ANOVA  *Follow-up independent samples t-test significantly different from other *at p < 0.05  ^3outliers removed   - There were significant main effects for work environment on sitting time (F(df)=4.2 (2,37), p<0.02) and self-report muscular strengthening PA (F(df)=3.6 (2,40), p<0.04).   Those who worked from home had significantly higher sitting times, higher step counts, and more days per week of muscular strengthening activity than those who worked from the office. | Funding:  Faculty Research Grant at Seattle Pacific University, an internal university award.  Conflicts of Interest:  The authors declare that they have no conflicts of  interest.  Overall RoB  high (-) |
| **Tomonaga et al.**  2024  Japan  No study name  (no underlying cohort)  06/2022-03/2023 | Statistical methods:  multivariate analysis of variance  Confounder:  age and weight, working hours, exercise habits | Comparison between Work From Home and Work in Office Including Commute (based on Table 2)   \| **Parameter** \| **Work in Office**  **LS means (95% CI)** \| **Work From Home**  **LS means (95%CI)** \| **P value** \| \| --- \| --- \| --- \| --- \| \| Energy consumption, kcal  Model 1  Model 2 \| 452.3 (422.4–482.3)  425.8 (390.0–461.7) \| 183.8 (153.9–213.8)  228.0 (192.1–263.9) \| <0.01  <0.01 \| \| Standing or  activity time, min  Model 1  Model 2 \| 186.7 (174.1–199.3)  177.0 (161.1–192.9) \| 87.8 (75.2–100.3)  107.2 (91.3–123.1) \| <0.01  <0.01 \| \| Sitting time, min  Model 1  Model 2 \| 569.2 (551.1–587.3)  501.7 (485.8–517.6) \| 513.6 (495.5–531.7)  571.6 (555.7–587.5) \| <0.01  <0.01 \| \| Sedentary break, count  Model 1  Model 2 \| 46.5 (43.2–49.8)  44.2 (40.2–48.2) \| 31.4 (28.1–34.7  37.4 (33.3–41.4) \| <0.01  <0.05 \| \| Sedentary bout >30 min, count  Model 1  Model 2 \| 5.5 (5.1–5.9)  4.8 (4.3–5.2) \| 5.3 (4.9–5.7)  5.6 (5.1-6.1) \| 0.49  <0.05 \| \| Sedentary bout >60 min, count  Model 1  Model 2 \| 1.8 (1.6–2.1)  1.4 (1.0–1.7) \| 1.9 (1.7–2.2)  2.0 (1.7–2.4) \| 0.52  <0.01 \|   P value for the multivariate analysis of variance.  Model 1 was adjusted for age and weight.  Model 2 was adjusted for age, weight, working hours (including commuting, lunch break and overwork), and exercise habits.  Comparison between Work From Home and Work in Office excluding Commute   \| **Parameter** \| **Work in Office**  **LS means (95% CI)** \| **Work From Home**  **LS means (95% CI)** \| **P value** \| \| --- \| --- \| --- \| --- \| \| Energy consumption, kcal  Model 3  Model 4 \| 244.8 (222.4–267.3)  252.7 (228–277.4) \| 182.9 (160.5–205.4)  193.0 (168.3–217.6) \| <0.01  <0.01 \| \| Standing or  activity time, min  Model 3  Model 4 \| 108.6 (97.6–119.6)  113.1 (100.7–125.5) \| 87.4 (76.4–98.3)  92.5 (80.1–104.9) \| <0.01  <0.01 \| \| Sitting time, min  Model 3  Model 4 \| 504.9 (486.4-523.4)  493.7 (481.3 –506.1) \| 512.3 (493.8–530.8)  514.1 (501.8–526.5) \| 0.53  <0.01 \| \| Sedentary break, count  Model 3  Model 4 \| 34.3(31.3–37.3)  36.1 (32.8–39.4) \| 31.3 (28.3–34.3)  33.5 (30.2–36.8) \| 0.09  0.14 \| \| Sedentary bout >30 min, count  Model 3  Model 4 \| 5.2 (4.8–5.6)  4.9 (4.5–5.3) \| 5.3 (4.9–5.7)  5.1 (4.7–5.5) \| 0.70  0.34 \| \| Sedentary bout >60 min, count  Model 3  Model 4 \| 1.7 (1.5–2.0)  1.5 (1.2-1.8) \| 1.9 (1.7–2.2)  1.8 (1.5–2.1) \| 0.18  0.10 \|   P value for the multivariate analysis of variance.  Model 3 was adjusted for age and weight.  Model 4 was adjusted for age, weight, working hours (including lunch break and overwork), and exercise habits.  In days working from home energy consumption was lower, sitting time was longer, number of sitting breaks was lower and time standing or in activity was lower than on days working at the office. This difference is more distinct when commuting time is included in the comparison. | Funding:  This study was supported by University of Occupational and Environmental Health Grant-in-Aid for Priority Research in the Field of Occupational Medicine (2022-15).  Conflict of interest:  none declared  Overall RoB:  low (+) |
| **Wahlström et al.**  2024  Sweden  No study name  (Flexible Work: opportunities and challenges (FLOC) cohort)10-12/2020, 05-06/2020 and 11-12/2020 | Statistical analysis  multilevel linear mixed models to account for the nested structure of the data by using the 3 different organizations and the workers as random effects  Compositional Data Analysis (CoDA) of outcome measures (see table)  Confounder: age, stratification by gender | Mean (SD) of the 5 physical behaviors, in minutes, and ILR1-4 according to CoDA, during days working at office (WAO), working from home (WFH) (based on table 2)   \|  \| **WAO** \| **WFH** \| \| --- \| --- \| --- \| \| **Non sit** \| 344.3 (113.0) \| 325.1 (110.2) \| \| **Short sit** (sitting without interruption for <5 min) \| 47.4 (18.8) \| 50.6 (22.3) \| \| **Moderate Sit** (sitting without interruption for 5-30 min) \| 234.6 (69.5) \| 231.4 (72.1) \| \| **longSit** (sitting without interruption for >30 min) \| 355.4 (126.3) \| 355.8 (138.6) \| \| **ILR2 ^1^** \| 0.68 (0.40) \| 0.62 (0.39) \| \| **ILR3 ^2^** \| −1.5 (0.42) \| −1.43 (0.45) \| \| **ILR4 ^3^** \| −0.28 (0.41) \| −0.27 (0.50) \|   IRL2 represents time spent non-sitting relative to sitting time during waking hours. IRL3 represents the time spent in short bouts of sitting (<5 min) relative to moderate and long bouts of sitting (5–30 min and >30 min), and ILR4 represents the time spent in moderate bouts of sitting (5–30 min) relative to long bouts of sitting  Note: TiBa Time in bed and and ILR1 (TiB/time awake) are not reported here  Effects of day type on relative time-use in physical behaviors, after adjustment for age (*n* =165) (based on table 3)   \|  \| **nonSit/ Sit (ILR2)** \| \| **shortSit/ moderateSit & longSit (ILR3)** \| \| **moderateSit/ longSit (ILR4)** \| \| \| --- \| --- \| --- \| --- \| --- \| --- \| --- \| \| **Day type** \| B \| CI  P value \| B \| CI  P value \| B \| CI  P value \| \| **WAO** \| −0.19^a^ \| −0.26; −0.11 <0.01 \| −0.32 \| −0.40; 0.23 <0.01 \| −0.15 \| −0.24; −0.05 <0.01 \| \| **WFH** \| −0.28 \| −0.35; −0.21 <0.01 \| −0.28 \| −0.37; 0.19 <0.01 \| −0.16 \| −0.27; −0.05 <0.01 \| \| **NWD^b^** \| Ref. \|  \| Ref. \|  \| Ref. \|  \|   ^a^significant difference (*P* < 0.05) in pairwise comparison of WAO and WFH  ^b^non working days  Mean values of sedentary behaviour in hours (based on personal communication)   \|  \| **Work at the office** \| **Work from home** \| \| --- \| --- \| --- \| \| **Mean sedentary time in h (SD)** \| 10.62 (1.9) \| 10.63 (1.8) \| \| **Median sedentary time in h** \| 10.94 \| 10.69 \| | Funding  The study was funded by FORTE (2019-01257). funders had no role or influence over the study design, data collection, analysis, interpretation of the data.  Conflict of interest  no conflict of interest  Overall RoB:  almost low (+/-) |
| **Wallmann Sperlich et al.**  2023  Germany  no study name  (no underlying cohort)  03-05/2021 | Statistical methods:  pearson correlation coefficients to analyse relationship between homeoffice proportion and sitting behaviour as well as PA  Confounder  no adjustment | Self-reported sedentary and PA behaviour during working hours of participants working partly or fully in the home office (HO) according to their HO proportion. (based on Table 2)   \|  \| **All (n=575)** \| **1–25% HO (n=63)^a^** \| **26–50% HO (n=99)^b^** \| **51–75% HO (n=56)^c^** \| **76–99% HO (n=120)^d^** \| **100% HO (n=237)^e^** \| **P^f^ (F)** \| **r^g^ (p)** \| \| --- \| --- \| --- \| --- \| --- \| --- \| --- \| --- \| --- \| \| **Sedentary and PA behaviour during working hours** \| \| \| \| \| \| \| \| \| \| **Proportion of workday sitting in %±SD (median)** \| 73.3  ±27.2 (80.0) \| 61.1  ±31.7 (70.0) \| 65.9  ±26.9 (80.0) \| 67.8  ±27.5 (80.0) \| 80.5  ±28.6 (85.0) \| 77.3  ±27.8 (90.0) \| *** (9.5) e>a,b d>a,b,c \| .234*** \| \| **Proportion of workday standing in%±SD (median)** \| 11.9  ±15.0 (10.0) \| 17.9  ±24.2 (10.0) \| 17.2  ±19.8 (10.0) \| 13.6  ±11.7 (10.0) \| 7.8  ±8.6 (5.0) \| 9.6  ±10.8 (9.3) \| *** (8.6) e>a,b d<a,b \| -.233*** \| \| **Proportion of workday walking in%±SD (median)** \| 13.4  ±13.9 (10.0) \| 14.5  ±13.3 (10.0) \| 16.6  ±12.0 (10.0) \| 16.7  ±15.7 (10.0) \| 11.3  ±11.9 (10.0) \| 11.9  ±14.9 (9.2) \| ** (3.4) \| -.130** \| \| **Proportion of workday physically demanding tasks in% ± SD (median)** \| 6.3  ±13.1 (0.0) \| 12.0  ±22.4 (0.0) \| 4.4  ±6.8 (0.0) \| 6.3  ±9.8 (0.0) \| 3.8  ±7.0 (0.0) \| 6.7  ±14.2 (0.0) \| ** (3.6) a>b,d \| -.076 \| \| **Number of sitting breaks per hour ± SD (median)** \| 2.1  ±1.6 (2.0) \| 2.1  ±1.6 (2.0) \| 2.6  ±1.7 (2.0) \| 1.6  ±1.3 (1.0) \| 2.3  ±1.8 (2.0) \| 1.9  ±1.5 (1.7) \| ** (4.4) b>c,e \| -.054 \|   ***p<0.001; **p<0.01.  ^a^Participants working 1–25% from the home office.  ^b^Participants working 26–50% from the home office.  ^c^Participants working 51–75% from the home office.  ^d^Participants working 75–99% from the home office.  ^e^Participants working 100% from the home office.  ^f^ (differences between HO categories)  ^g^ Pearson correlation coefficient (sedentary and PA behaviour during working hours and HO proportion)   - Overall, the proportion of HO was positively correlated with the workday sitting proportion (r=0.234, p<0.001) and negatively correlated with workday standing (r=0.233, p<0.001) as well as the workday walking proportion (r=0.130; p<0.01). - Concerning the five groups according to the amount of HO:   - the workday sitting proportion of 80.5±18.6%, respectively 77.3±27.8% was highest in workers working 76–99%, respectively 100% from HO (p<0.001) compared to groups with lower HO proportion   - workday standing proportion was lower in workers working 76–99% from HO with 7.8±8.6%, respectively 100% from HO with 9.6±10.8% standing than in workers working 1–25%, respectively 26–50% from HO (p < 0.001)   - Sitting breaks/h were higher in workers working 26–50% in HO than in workers working 51–75%, respectively 100% in HO (p<0.05) | Funding:  data collection was supported by ERGO Insurance Group AG  Conflicts of Interest:  The authors declare that they have no conflicts of interest.  Overall RoB  high (-) |
| **Webber et al.**  2024  USA  no study name  (Summer Styles)  21/05-6/06/2022 | Statistical methods:  weighted prevalence  and 95% confidence intervals (CIs) of changes in each of the three  PA behaviors were estimated using log-binominal regression  Confounder:  Age, race/ethnicity, education, marital status, income and employment status (full-time or part-time | Perceived Decreases in PA Behaviours and Prevalence of Meeting the Aerobic Physical Activity Guidelines by Reported Changes in Telework Among the Employed US Adults – SummerStyles 2022 (n=2391) (based on table 3 and table 4)   \|  \| **Prevalence, %**  **(95% CI)** \| **Unadjusted PR**  **(95% CI)** \| **Adjusted PR**  **(95% CI)** \| \| --- \| --- \| --- \| --- \| \| **Decreased PA** \|  \|  \|  \| \| Stable telework \| 13.0 (10.7 -15.3) \| 1.00 \| 1.00 \| \| More telework \| 19.2 (15.9 - 22.5) \| 1.48 (1.21-1.80) \| 1.61 (1.30-1.98) \| \| Less telework \| 32.3 (24.1 – 40.5) \| 2.49 (1.96-3.16) \| 2.43 (1.91-3.10) \| \| **Decreased active transportation** \|  \|  \|  \| \| Stable telework \| 11.9 (9.7-14.1) \| 1.00 \| 1.00 \| \| More telework \| 16.8 (13.6-20.1) \| 1.41 (1.14-1.74) \| 1.65 (1.32-2.07) \| \| Less telework \| 33.4 (25.1-41.8) \| 2.80 (2.21-3.56) \| 2.66 (2.10-3.38) \| \| **Decreased use of parks and trails** \|  \|  \|  \| \| Stable telework \| 12.8 (10.6-15.0) \| 1.00 \| 1.00 \| \| More telework \| 16.7 (13.5-19.9) \| 1.20 (1.06-161) \| 1.52 (1.22-1.89) \| \| Less telework \| 42.5 (33.9-51.2) \| 3.32 (2.69-4.10) \| 3.05 (2.49-3.74) \| \| **Meeting aerobic guidelines** \|  \|  \|  \| \| Stable telework \| 51.4 (48.2-54.7) \| 1.00 \| 1.00 \| \| More telework \| 60.0 (55.8-64.1) \| 1.17 (1.08-1.26) \| 1.03 (0.95-1.11) \| \| Less telework \| 51.7 (43.1-60.3) \| 1.01 (0.87-1.16) \| - 1. 0.88-1.17) \|  - those who reported more telework and less telework, compared with those who reported stable levels (i.e., same or never), were more likely to perceive a decrease in any PA, active transportation, and use of parks and trails. - Compared with 13.0% of those with stable telework, 19.2% of those with more telework reported decreased PA, and 32.3% of those with less telework reported decreased PA - 51.4% of participants who had stable telework reported a level of leisure-time PA that meets the aerobic guideline, compared with 60.0% of those who had reported more and 51.7% of those who reported less.   Perceived Decrease in Physical Activity Behaviours and Prevalence of Meeting the Aerobic Physical Activity Guidelines by Reported changes in Telework Among Employed U.S. Adults, with Never Teleworks as Referent Group – SummerStyles 2022 (n=2391) (based on Supplementary Table 2)   \|  \| **Prevalence, %**  **(95% CI)** \| **Unadjusted PR**  **(95% CI)** \| **Adjusted PR**  **(95% CI)** \| \| --- \| --- \| --- \| --- \| \| **Decreased PA** \|  \|  \|  \| \| Never telework \| 15.5 (12.5, 18.6) \| 1.00 \| 1.00 \| \| More telework \| 19.2 (15.9, 22.5) \| 1.24 (1.00, 1.53) \| 1.37 (1.09, 1.72) \| \| Same telework \| 8.6 (5.5, 11.8) \| 0.56 (0.40, 0.76) \| 0.61 (0.44, 0.84) \| \| Less telework \| 32.3 (24.1, 40.5) \| 2.08 (1.62, 2.67) \| 2.09 (1.61, 2.70) \| \| Decreased active transportation \|  \|  \|  \| \| Never telework \| 12.7 (9.8, 15.6) \| 1.00 \| 1.00 \| \| More telework \| 16.8 (13.6, 20.1) \| 1.33 (1.05, 1.67) \| 1.63 (1.27, 2.09) \| \| Same telework \| 10.6 (7.1, 14.1) \| 0.84 (0.62, 1.13) \| 0.96 (0.71, 1.31) \| \| Less telework \| 33.4 (25.1, 41.8) \| 2.64 (2.03, 3.42) \| 2.63 (2.03, 3.40) \| \| **Decreased use of parks and trails** \|  \|  \|  \| \| Never telework \| 15.4 (12.4, 18.5) \| 1.00 \| 1.00 \| \| More telework \| 16.7 (13.5, 19.9) \| 1.08 (0.87, 1.35) \| 1.30 (1.02, 1.64) \| \| Same telework \| 8.4 (5.4, 11.3) \| 0.54 (0.39, 0.75) \| 0.61 (0.44, 0.84) \| \| Less telework \| 42.5 (33.9, 51.2) \| 2.76 (2.21, 3.44) \| 2.66 (2.14, 3.30) \| \| **Meeting aerobic guidelines** \|  \|  \|  \| \| Never telework \| 48.4 (44.3, 52.5) \| 1.00 \| 1.00 \| \| More telework \| 60.0 (55.8, 64.1) \| 1.24 (1.13, 1.35) \| 1.06 (0.97, 1.17) \| \| Same telework \| 56.6 (51.4, 61.8) \| 1.17 (1.06, 1.29) \| 1.07 (0.97, 1.18) \| \| Less telework \| 51.7 (43.1, 60.3) \| 1.07 (0.92, 1.24) \| - 1. 0.90, 1.21) \|  - Patterns largely held when never telework, instead of stable telework, was used as the referent group. | Funding:  This research was supported by the Centers for Disease Control and Prevention.  Conflicts of Interest:  none declared  Overall RoB:  high (-) |
| **Widar et al.**  2021  Sweden  no study name  (underlying cohort^[[14]](#footnote-14)^)  08/2018-06/2019 | Statistical methods:  repeated measures analyses of variance (ANOVA) were performed with Workplace (two levels) and Time (three and six levels) as within-subjects effect  ANOVAs were performed with and without adjustment  level of significance was set to P < 0.05 in all tests  Confounder:  commuting time, children living at home, gender | - Compositional data analysis: The isometric log-ratio (ilr) transformed SB (sitting time related to time standing or moving) relative to other behaviours showed no significant interaction effect of Workplace and Time (ղ2=0.013) but a significant effect of Time (ղ2=0.480) (Table 3)   Estimated geometrical means for time (min) spent in different physical behaviours before, during and after workhours on teleworking days and office days (n=23) (based on Table 4)   \| **Variable description** \|  \| **Before work** \| **During work** \| **After work** \| **Total time** \| \| --- \| --- \| --- \| --- \| --- \| --- \| \| **Sit/lie** \| Telework  Office \| 34  41 \| 324  312 \| 193  222 \| 551  575 \| \| **Stand** \| Telework  Office \| 20  20 \| 78  73 \| 57  64 \| 155  157 \| \| **Move*** \| Telework  Office \| 15  26 \| 66  51 \| 54  66 \| 135  143 \|   *physical movements such as walking, running, cycling  The Workplace (telework; office) and time (leisure time before workhours; work during regular workhours; leisure time after workhours) effects and interaction effect for postures and movement (n=23) (based on table 3)   \|  \|  \| **F** \| ***p** \| **Partial η^2^** \| **Power** \| \| --- \| --- \| --- \| --- \| --- \| --- \| \| **Number of transitions sit to stand** \| Time \| 63.853 \| **0.000** \| 0.753 \| 1.000 \| \| Workplace \| 0.036 \| 0.851 \| 0.002 \| 0.054 \| \| Time x Workplace \| 5.059 \| **0.021** \| 0.194 \| 0.688 \|   *significant differences are shown in bold, p <0.05.   - most time spent in sedentary position was seen during regular workhours and during leisure time after workhours - There was a significant Workplace and Time interaction effect for the variation in movements, i.e., transitions between sitting and standing (ղ2=0.194; p=0.021), with more transitions being made during teleworking hours (Mean=31) than during office hours (Mean=25 Table 3). | Funding:  No external funding, the University of Gävle funded this project and had no influence on the design, process or conduct of this study.  Conflicts of Interest:  The authors declare no conflicts of interest.  Overall RoB:  low (+) |

1. Barone Gibbs B, Conroy MB, Huber K, et al. Effect of Reducing Sedentary Behavior on Blood Pressure (RESET BP): rationale, design, and methods. Contemp Clin Trials. 2021; 106:106428. [↑](#footnote-ref-1)
2. Study characteristics extracted partially from: Bérard, E.; Huo Yung Kai, S.; Coley, N.; Bongard, V.; Ferrières, J. One-Year Impact of COVID-19 Lockdown-Related Factors on Cardiovascular Risk and Mental Health: A Population-Based Cohort Study. Int. J. Environ. Res. Public Health 2022, 19, 1684. https:// doi.org/10.3390/ijerph19031684. [↑](#footnote-ref-2)
3. Marie Pigeyre, Luc Dauchet, Chantal Simon, Vanina Bongard, Annie Bingham, Dominique Arveiler, Jean-Bernard Ruidavets, Aline Wagner, Jean Ferrières, Philippe Amouyel, Jean Dallongeville. Effects of occupational and educational changes on obesity trends in France: The results of the MONICA-France survey 1986–2006. Preventive Medicine 2011, Volume 52, Issue 5, https://doi.org/10.1016/j.ypmed.2011.02.004 [↑](#footnote-ref-3)
4. Aquino, E. M. et al. (2013). Recrutamento de participantes no Estudo Longitudinal de Saúde do Adulto [Participants recruitment in ELSA-Brasil (Brazilian Longitudinal Study for Adult Health)]. Revista de saude publica, 47 Suppl 2, 10–18. https://doi.org/10.1590/s0034-8910.2013047003953) [↑](#footnote-ref-4)
5. Masaki Machida, Itaru Nakamura, Reiko Saito, Tomoki Nakaya, Tomoya Hanibuchi, Tomoko Takamiya, Yuko Odagiri, Noritoshi Fukushima, Hiroyuki Kikuchi, Takako Kojima, Hidehiro Watanabe, Shigeru Inoue. Adoption of personal protective measures by ordinary citizens during the COVID-19 outbreak in Japan. International Journal of Infectious Diseases, Volume 94, 2020, Pages 139-144, ISSN 1201-9712, https://doi.org/10.1016/j.ijid.2020.04.014. [↑](#footnote-ref-5)
6. https://www.lissdata.nl/about-panel/composition-and-response [↑](#footnote-ref-6)
7. Svensson S, Hallman DM, Mathiassen S, et al. Flexible Work: Opportunity and Challenge (FLOC) for individual, social and economic sustainability. Protocol for a prospective cohort study of non-standard employment and flexible work arrangements in Sweden. BMJ Open 2022;12: e057409. doi:10.1136/ bmjopen-2021-057409 [↑](#footnote-ref-7)
8. Barone Gibbs B, Conroy MB, Huber K, et al. Effect of Reducing Sedentary Behavior on Blood Pressure (RESET BP): rationale, design, and methods. Contemp Clin Trials. 2021; 106:106428. [↑](#footnote-ref-8)
9. Barone Gibbs B, Conroy MB, Huber K, et al. Effect of Reducing Sedentary Behavior on Blood Pressure (RESET BP): rationale, design, and methods. Contemp Clin Trials. 2021; 106:106428. [↑](#footnote-ref-9)
10. Buck N, McFall S. Understanding Society: design overview. Longitudinal and Life course Studies. 2012; 3(1):5-17.

    [↑](#footnote-ref-10)
11. Heiden, M., Widar, L., Wiitavaara, B. *et al.* Telework in academia: associations with health and well-being among staff. *High Educ* **81**, 707–722 (2021). https://doi.org/10.1007/s10734-020-00569-4 [↑](#footnote-ref-11)
12. Study characteristics extracted partially from: Bérard, E.; Huo Yung Kai, S.; Coley, N.; Bongard, V.; Ferrières, J. One-Year Impact of COVID-19 Lockdown-Related Factors on Cardiovascular Risk and Mental Health: A Population-Based Cohort Study. Int. J. Environ. Res. Public Health 2022, 19, 1684. https:// doi.org/10.3390/ijerph19031684. [↑](#footnote-ref-12)
13. Svensson S, Hallman DM, Mathiassen S, et al. Flexible Work: Opportunity and Challenge (FLOC) for individual, social and economic sustainability. Protocol for a prospective cohort study of non-standard employment and flexible work arrangements in Sweden. BMJ Open 2022;12: e057409. doi:10.1136/ bmjopen-2021-057409 [↑](#footnote-ref-13)
14. Heiden, M., Widar, L., Wiitavaara, B. et al. Telework in academia: associations with health and well-being among staff. High Educ 81, 707–722 (2021). https://doi.org/10.1007/s10734-020-00569-4 [↑](#footnote-ref-14)
