# Supplementary material for: The impact of working from home on sedentary behaviour and physical activity compared to onsite work in the working population: a systematic review and meta-analysis
Source: BMC Public Health. 2025 Nov 17;25:3963. doi: 10.1186/s12889-025-24960-x (PMC12621373; doi:10.1186/s12889-025-24960-x)
Supplement: Supplementary file 3 — Additional file 3: List of excluded studies (Authors and references with reason for exclusion). [file 12889_2025_24960_MOESM3_ESM.docx]

## Additional file 3 – documentation of excluded studies

# Excluded articles and main reason for exclusion

| Ref. | Autor (publication year) | Main reason for exclusion |
| --- | --- | --- |
| (1) | Aktan (2022) | wrong comparison |
| (2) | Alpers (2023) | wrong population |
| (3) | Alpers (2024) | wrong population |
| (4) | Anugrahsari (2021) | wrong study design |
| (5) | Argus (2021) | wrong exposure |
| (6) | Arjmand (2023) | wrong exposure |
| (7) | Bailly (2022) | wrong exposure |
| (8) | Barğı (2024) | wrong exposure |
| (9) | Borgatti (2021) | wrong exposure |
| (10) | Borowski (2021) | wrong comparison |
| (11) | Bosma (2023) | wrong outcome |
| (12) | Bosnjak (2022) | wrong publication type |
| (13) | Bouden (2024) | no comparison |
| (14) | Brancaccio (2021) | wrong exposure |
| (15) | Brusaca (2021) | wrong exposure |
| (16) | Brusaca (2022) | wrong comparison |
| (17) | Chang (2023) | wrong comparison |
| (18) | Corrigan (2021) | wrong publication type |
| (19) | Coskun (2022) | wrong comparison |
| (20) | Cousins (2023) | wrong exposure |
| (21) | Cross (2021) | wrong publication type |
| (22) | De Santana (2022) | wrong exposure |
| (23) | Delgado-Ortiz (2023) | wrong exposure |
| (24) | De Lima (2024) | No comparison |
| (25) | DeSimone (2022) | wrong study design |
| (26) | Di Fusco (2021) | wrong study design |
| (27) | Dillon-Rossiter (2023) | wrong comparison |
| (28) | Dos Santos (2021) | wrong study design |
| (29) | Dos Santos Ferreira Viero (2022) | wrong exposure |
| (30) | Duteil (2025) | Wrong population |
| (31) | El Kadri Filho (2022) | wrong comparison |
| (32) | Elvén (2022) | wrong exposure |
| (33) | Fiorini (2022) | wrong publication type |
| (34) | Fiorini (2023) | wrong outcome |
| (35) | Forte (2021) | wrong exposure |
| (36) | Fouquet (2025) | wrong outcome |
| (37) | Füzéki (2021) | wrong population |
| (38) | Gallé (2022) | wrong exposure |
| (39) | Garcia (2022) | only lockdown effect |
| (40) | Gonzales (2022) | wrong exposure |
| (41) | Gorska (2021) | wrong exposure |
| (42) | Griffiths (2022) | wrong exposure |
| (43) | Guler (2021) | wrong exposure |
| (44) | Güney-Coskun (2021) | wrong publication type |
| (45) | Gupta (2023) | wrong exposure |
| (46) | Haddad (2024) | Wrong outcome |
| (47) | Holmes (2021) | wrong publication type |
| (48) | Houtenbos (2024) | wrong exposure |
| (49) | Howe (2021) | only lockdown effect |
| (50) | Jalalvand (2021) | wrong study design |
| (51) | Jepson (2022) | wrong exposure |
| (52) | Kakar (2020) | wrong publication type |
| (53) | Kantyka (2024) | wrong population |
| (54) | Kawada (2023) | wrong exposure |
| (55) | Kayali (2021) | wrong publication type |
| (56) | Kim (2024) | wrong outcome |
| (57) | Kishimoto (2021) | wrong exposure |
| (58) | Koohsari (2021) | only lockdown effect |
| (59) | Kuk (2021) | wrong outcome |
| (60) | Lindsey (2021) | wrong publication type |
| (61) | Martin (2008) | wrong population |
| (62) | Martinez (2021) | wrong exposure |
| (63) | Massar (2022) | wrong exposure |
| (64) | McDowell (2020) | wrong exposure |
| (65) | Michailidou (2022) | wrong comparison |
| (66) | Michalchuk (2022) | wrong study design |
| (67) | Miura (2024) | wrong outcome |
| (68) | Moreira (2022) | wrong exposure |
| (69) | Morton (2022) | wrong study design |
| (70) | Morton (2022) | wrong outcome |
| (71) | Nagata (2021) | wrong population |
| (72) | Niu (2021) | wrong exposure |
| (73) | Niven (2023) | wrong comparison |
| (74) | Olsen (2014) | wrong publication type |
| (75) | Olsen (2018) | wrong exposure |
| (76) | Papalia (2022) | wrong study design |
| (77) | Petroni (2020) | wrong publication type |
| (78) | Pino-Casal (2021) | foreign language |
| (79) | Radulovic (2021) | wrong exposure |
| (80) | Rapisarda (2021) | only lockdown effect |
| (81) | Roberts (2021) | wrong exposure |
| (82) | Schifferli (2025) | Foreign language |
| (83) | Scoditti (2024) | wrong exposure |
| (84) | Seker (20249 | wrong exposure |
| (85) | Shifrin (2022) | wrong study design |
| (86) | Silvestre (2023) | wrong outcome |
| (87) | Smite (2023) | wrong exposure |
| (88) | Somasundram (2022) | wrong comparison |
| (89) | Sparks (2021) | wrong exposure |
| (90) | Tazegül (2022) | wrong publication type |
| (91) | Tobin (2021) | wrong publication type |
| (92) | Valecha (2021) | wrong publication type |
| (93) | Wakaizumi (2021) | only lockdown effect |
| (94) | Weale (2023) | wrong exposure |
| (95) | Werneck (2021) | wrong exposure |
| (96) | Wiezer (2022) | wrong publication type |
| (97) | Wilke (2021) | wrong exposure |
| (98) | Xiao (2021) | wrong exposure |
| (99) | Yang (2021) | wrong population |
| (100) | Yan (2021) | wrong publication type |
| (101) | Yoshimoto (2021) | only lockdown effect |
| (102) | Zhou (2024) | only lockdown effect |

# References

1. Aktan B, Gamsızkan Z. Physical activity and eating changes during lockdown periods during the COVID-19 pandemic – threat of obesity pandemic. Family Medicine & Primary Care Review. 2022;24(3):197-201.

2. Alpers SE, Pallesen S, Vold JH, Haug E, Lunde LH, Skogen JC, et al. The association between psychological distress and alcohol consumption and physical activity: a population-based cohort study. Frontiers in Psychiatry. 2023;14:1181046.

3. Alpers SE, Druckrey-Fiskaaen KT, Madebo T, Vold JH, Pallesen S, Skogen JC, et al. The association of psychological distress and economic and health worries with tobacco smoking behavior during the COVID-19 pandemic: a two-year longitudinal cohort study. BMC Public Health. 2024;24(1):375.

4. Anugrahsari S, Abba H, Suryadi. Physical activity and daily exercise-related social distance policy during Covid-19 pandemic: A systematic literature review. Journal of Physical Education & Sport. 2021;21:2440-6.

5. Argus M, Pääsuke M. Effects of the COVID-19 lockdown on musculoskeletal pain, physical activity, and work environment in Estonian office workers transitioning to working from home. Work (Reading, Mass). 2021;69(3):741-9.

6. Arjmand EJ, Skogen JC, Vold JH, Alpers SE, Arnesen EK, Maeland S, et al. Changes in Body Mass Index and Their Associations with Psychological Distress, Worries, and Emotional Eating during the COVID-19 Pandemic: A Norwegian Cohort Study. Nutrients. 2023;15(17).

7. Bailly F, Genevay S, Foltz V, Bohm-Sigrand A, Zagala A, Nizard J, et al. Effects of COVID-19 lockdown on low back pain intensity in chronic low back pain patients: results of the multicenter CONFI-LOMB study. European spine journal : official publication of the European Spine Society, the European Spinal Deformity Society, and the European Section of the Cervical Spine Research Society. 2022;31(1):159-66.

8. Bargi G, Suner-Keklik S. Effects of short-term upper extremity exercise training in office workers during COVID-19 restrictions: A randomized controlled trial. Work. 2024;78(4):1187-99.

9. Borgatti AC, Schneider-Worthington CR, Stager LM, Krantz OM, Davis AL, Blevins M, et al. The COVID-19 pandemic and weight management: Effective behaviors and pandemic-specific risk factors. Obesity Research & Clinical Practice 2021;15(5):518-21.

10. Borowski S, Savla J, Zvonkovic AM. Impact of Flexible Work Arrangements, Self-Efficacy, and Barriers on Daily Physical Activity Among University Staff. Journal of physical activity & health. 2021;18(5):594-602.

11. Bosma E, Loef B, van Oostrom SH, Proper KI. The longitudinal association between working from home and musculoskeletal pain during the COVID-19 pandemic. International archives of occupational and environmental health. 2023;96(4):521-35.

12. Bosnjak E, Benard J, Ohler A. COVID-19 and Healthy Habitsin Missouri Families. Pediatrics. 2022;149.

13. Bouden S, Loukil S, Mhamdi S, Rouached L, Ben Tekaya A, Mahmoud I, et al. Ab0378 Musculoskeletal Disorders during Teleworking. Annals of the Rheumatic Diseases. 2024;83.

14. Brancaccio M, Mennitti C, Gentile A, Correale L, Buzzachera CF, Ferraris C, et al. Effects of the COVID-19 Pandemic on Job Activity, Dietary Behaviours and Physical Activity Habits of University Population of Naples, Federico II-Italy. International journal of environmental research and public health. 2021;18(4).

15. Brusaca LA, Barbieri DF, Mathiassen SE, Holtermann A, Oliveira AB. Physical Behaviours in Brazilian Office Workers Working from Home during the COVID-19 Pandemic, Compared to before the Pandemic: A Compositional Data Analysis. International journal of environmental research and public health. 2021;18(12).

16. Brusaca LA, Januario LB, Mathiassen SE, Barbieri DF, Oliveira RV, Heiden M, et al. Sedentary behaviour, physical activity, and sleep among office workers during the COVID-19 pandemic: a comparison of Brazil and Sweden. BMC Public Health. 2022;22(1):2196.

17. Chang Q. PHYSICAL EXERCISE IN REMOTE EMPLOYEES DURING COVID-19. Revista brasileira de medicina do Esporte. 2023;29.

18. Corrigan RJ, Hillman A, Chase P, de Faria FR, Johanna Z, Howe CA. Impact Of Covid-19 Stay-at-home Restrictions And Employment Changes On Physical Activity And Sedentary Behavior Changes. Medicine & Science in Sports & Exercise. 2021;53:324-5.

19. Coşkun MG, Öztürk Rİ, Tak AY, Sanlier N. Working from Home during the COVID-19 Pandemic and Its Effects on Diet, Sedentary Lifestyle, and Stress. Nutrients. 2022;14(19).

20. Cousins DJE, Schaefer BH, Holmes MWR, Beaudette SM. The effects of COVID-19 related shutdowns on perceived lifestyle and prevalence of musculoskeletal discomfort. Work. 2023;76(1):11-20.

21. Cross TJ, Isautier JMJ, Ziegler BL, Morris SJ, Johnson BD, Wheatley C, et al. The Paws Project: The Impact Of Social Distancing On Physical Activity During The COVID-19 Pandemic. Medicine & Science in Sports & Exercise. 2021;53:191-.

22. de Santana WF, Tavares GH, Pires LC, Romano FS, de Oliveira NRC, Lusby C, et al. The decrease in the physical activity levels during the COVID-19 social distancing period. Motriz Revista Educacao Fisica. 2022;28.

23. Delgado-Ortiz L, Carsin AE, Merino J, Cobo I, Koch S, Goldberg X, et al. Changes in Population Health-Related Behaviors During a COVID-19 Surge: A Natural Experiment. Annals of behavioral medicine : a publication of the Society of Behavioral Medicine. 2023;57(3):216-26.

24. de Lima MNM, Oliveira GTA, Araujo AO, Pereira LC, Cevada T, NSS ES, et al. The relationship between physical inactivity, excessive screen time, and health indicators among teachers working from home during the COVID-19 pandemic: A cross-sectional study. Work. 2025;80(3):1110-21.

25. DeSimone GT. Shareable Resource: Take a Stand Against Sitting. ACSM's Health & Fitness Journal. 2022;29(6):4-.

26. Di Fusco SA, Spinelli A, Castello L, Mocini E, Gulizia MM, Oliva F, et al. Impact of Working from Home on Cardiovascular Health: An Emerging Issue with the COVID-19 Pandemic. International journal of environmental research and public health. 2021;18(22).

27. Dillon-Rossiter K, Hiemstra M, Bartmann N, Sui W, Mitchell M, Rollo S, et al. Validity of the Modified SIT-Q 7d for Estimating Sedentary Break Frequency and Duration in Home-Based Office Workers During the COVID-19 Global Pandemic: A Secondary Analysis. Journal for the Measurement of Physical Behaviour. 2023;6(1):60-72.

28. Dos Santos IN, Pernambuco ML, da Silva AMB, Ruela GA, de Oliveira AS. Association between musculoskeletal pain and telework in the context of the COVID 19 pandemic: an integrative review. Revista brasileira de medicina do trabalho : publicacao oficial da Associacao Nacional de Medicina do Trabalho-ANAMT. 2021;19(3):342-50.

29. Dos Santos Ferreira Viero V, Matias TS, Alexandrino EG, Vieira YP, Meller FO, Schäfer AA, et al. Physical activity pattern before and during the COVID-19 pandemic and association with contextual variables of the pandemic in adults and older adults in southern Brazil. Zeitschrift fur Gesundheitswissenschaften = Journal of public health. 2022:1-9.

30. Dutheil F, Saint-Arroman C, Clinchamps M, Flaudias V, Fantini ML, Pereira B, et al. Influence of Socio-Demographic, Occupational and Lifestyle Variables on Sleep Time. Nat Sci Sleep. 2025;17:195-210.

31. El Kadri Filho F, Lucca SRD. Telework Conditions, Ergonomic and Psychosocial Risks, and Musculoskeletal Problems in the COVID-19 Pandemic. Journal of Occupational and Environmental Medicine. 2022;64(12):E811-E7.

32. Elvén M, Kerstis B, Stier J, Hellström C, von Heideken Wågert P, Dahlen M, et al. Changes in Physical Activity and Sedentary Behavior before and during the COVID-19 Pandemic: A Swedish Population Study. International Journal of Environmental Research and Public Health. 2022;19(5).

33. Fiorini LA. Remote workers’ perceived health during the COVID-19 pandemic: a mixed methods study of influencing factors. Safety and Health at Work. 2022;13:S194.

34. Fiorini LA. Remote workers' perceived health during the COVID-19 pandemic: an exploratory study of influencing factors in the IT and communications sector in Malta. Industrial Health. 2023;61(5):342-56.

35. Forte T, Santinha G, Carvalho SA. The COVID-19 Pandemic Strain: Teleworking and Health Behavior Changes in the Portuguese Context. Healthcare. 2021;9(9).

36. Fouquet N, Menard C, Fadel M, Bodin J, Roquelaure Y. Onset of low back pain and teleworking conditions during lockdown in France: a structural equation modelling approach. Ann Work Expo Health. 2025;69(5):531-43.

37. Füzéki E, Schröder J, Groneberg DA, Banzer W. Physical Activity and Its Related Factors during the First COVID-19 Lockdown in Germany. Sustainability. 2021;13(10).

38. Gallé F, Quaranta A, Napoli C, Diella G, De Giglio O, Caggiano G, et al. How do Vaccinators Experience the Pandemic? Lifestyle Behaviors in a Sample of Italian Public Health Workers during the COVID-19 Era. Vaccines. 2022;10(2).

39. Garcia MG, Aguiar B, Bonilla S, Yepez N, Arauz PG, Martin BJ. Perceived Physical Discomfort and Its Associations With Home Office Characteristics During the COVID-19 Pandemic. Human factors. 2022:187208221110683.

40. Gonzales A, Lin JH, Cha JS. Physical activity changes among office workers during the COVID-19 pandemic lockdown and the agreement between objective and subjective physical activity metrics. Applied ergonomics. 2022;105:103845.

41. Górska P, Górna I, Miechowicz I, Przysławski J. Changes in Life Situations during the SARS-CoV-2 Virus Pandemic and Their Impact on Eating Behaviors for Residents of Europe, Australia as Well as North and South America. Nutrients. 2021;13(10).

42. Griffiths ML, Gray BJ, Kyle RG, Song J, Davies AR. Exploring the health impacts and inequalities of the new way of working: findings from a cross-sectional study. Journal of Occupational and Environmental Medicine. 2022.

43. Guler MA, Guler K, Gulec MG, Ozdoglar E. Working From Home During a Pandemic: Investigation of the Impact of COVID-19 on Employee Health and Productivity. Journal of Occupational and Environmental Medicine. 2021;63(9):731-41.

44. Güney-Coşkun M, Öztürk RI. Working from home during the COVID-19 pandemic and its' effects on stress, sedentary lifestyle, and diet. Annals of nutrition and metabolism. 2021;77(6):356.

45. Gupta G, Jadhav RA, Nataraj M, Maiya GA. Effect of Covid-19 lockdown/ compulsory work from home (WFH) situation on musculoskeletal disorders in India. Journal of bodywork and movement therapies. 2023;33:39-45.

46. Haddad C, Zakhour M, Sacre H, Karam J, El Khatib S, Akel M, et al. Perceived advantages and drawbacks of teleworking during the economic crisis and COVID-19: A cross-sectional study among university degree holders. Arch Environ Occup Health. 2024;79(2):91-105.

47. Holmes AJ, Quinn TD, Paley JL, Conroy MB, Barone Gibbs B. Associations Of The Physical Workplace Environment With Sedentary Behaviour At Work. Medicine & Science in Sports & Exercise. 2021;53:236-.

48. <Houtenbos_2024_Poster_Congress_ISPNE_2024_Houtenbos.pdf>.

49. Howe CA, Corrigan RJ, FR dF, Johanni Z, Chase P, Hillman AR. Impact of COVID-19 Stay-at-Home Restrictions on Employment Status, Physical Activity, and Sedentary Behavior. International journal of environmental research and public health. 2021;18(22).

50. Jalalvand D, Moghaddam AM, Eshaghzadeh M, Nejad FM. Detection of back pains among office workers using mri. Journal of Pain and Symptom Management. 2021;14(2):179-81.

51. Jepson R, Baker G, Sivaramakrishnan D, Manner J, Parker R, Lloyd S, et al. Feasibility of a theory-based intervention to reduce sedentary behaviour among contact centre staff: the SUH stepped-wedge cluster RCT. Southampton (UK)2022 2022-12.

52. Kakar RS, Lomond K. Can COVID-19 Lead to Another Pandemic of Back Pain? Archives of Physical Medicine and Rehabilitation. 2020;101(12):e132.

53. Kantyka J, Maciąg J. Impact of Remote Working on Recreational Physical Activity (RPA) Behaviours – a Case Study of the Covid-19 Lockdown in Poland. Physical Culture and Sport Studies and Research. 2024;103(1):45-56.

54. Kawada S, Maeno T, Yokoya S, Maeno T. Factors associated with decreased physical activity levels among community-dwelling residents during the coronavirus disease 2019 pandemic: a long-term observational study. Journal of physical therapy science. 2023;35(6):447-54.

55. Kayali S, Furnari M, Ziola S, Giuliana E, Casagrande E, Djahandideh Sheijani A, et al. Impact of environmental changes forced by pandemic restrictive measures on the development and course of irritable bowel syndrome. United European Gastroenterology Journal. 2021;9:632-3.

56. Kim HJ, Lee DW, Choi J, Hong YC, Kang MY. Association between work from home and health-related productivity loss among Korean employees. Ann Occup Environ Med. 2024;36:e13.

57. Kishimoto M, Ishikawa T, Odawara M. Behavioral changes in patients with diabetes during the COVID-19 pandemic. Diabetology international. 2021;12(2):241-5.

58. Koohsari MJ, Nakaya T, McCormack GR, Shibata A, Ishii K, Oka K. Changes in Workers' Sedentary and Physical Activity Behaviors in Response to the COVID-19 Pandemic and Their Relationships With Fatigue: Longitudinal Online Study. JMIR public health and surveillance. 2021;7(3):e26293.

59. Kuk JL, Christensen RAG, Kamran Samani E, Wharton S. Predictors of Weight Loss and Weight Gain in Weight Management Patients during the COVID-19 Pandemic. Journal of obesity. 2021;2021:4881430.

60. Lindsey B, Boolani A, Merrigan J, Cortes N, Caswell S, Martin J. Employment Status is Related to Time Sitting but not Physcial Activity During THE COVID-19 Pandemic. Medicine & Science in Sports & Exercise. 2021;53:188-.

61. Martín AR, Nieto JM, Ruiz JP, Jiménez LE. Overweight and obesity: the role of education, employment and income in Spanish adults. Appetite. 2008;51(2):266-72.

62. Martínez L, Valencia I, Trofimoff V, Vidal N, Robles E, Duque JC, et al. Quality of life, health, and government perception during COVID-19 times: Data from Colombia. Data in brief. 2021;37:107268.

63. Massar SAA, Ong JL, Lau T, Ng BKL, Chan LF, Koek D, et al. Working-from-home persistently influences sleep and physical activity 2 years after the Covid-19 pandemic onset: a longitudinal sleep tracker and electronic diary-based study. Frontiers in psychology. 2023;14:1145893.

64. McDowell CP, Herring MP, Lansing J, Brower C, Meyer JD. Working From Home and Job Loss Due to the COVID-19 Pandemic Are Associated With Greater Time in Sedentary Behaviors. Frontiers in public health. 2020;8:597619.

65. Michailidou C, Charalambous L, King C, Tsangari H. Pain and stiffness resulting from an imposed sedentary lifestyle during lockdown, in the university community. Archives of Hellenic Medicine 2022;39(6):772-80.

66. Michalchuk VF, Lee S-J, Waters CM, Hong OS, Fukuoka Y. Systematic Review of the Influence of Physical Work Environment on Office Workers' Physical Activity Behavior. Workplace Health Saf. 2022;70(2):97-119.

67. Miura M, Tabuchi T, Amano H, Katanoda K. Evaluation of the Association Between Sedentary Time and Low Work Engagement in the Work Environment After COVID-19 Pandemic: A Cross-Sectional Study of Japanese Workers. Cureus. 2024;16(6):e62725.

68. Moreira S, Criado MB, Ferreira MS, Machado J, Gonçalves C, Mesquita C, et al. The Effects of COVID-19 Lockdown on the Perception of Physical Activity and on the Perception of Musculoskeletal Symptoms in Computer Workers: Comparative Longitudinal Study Design. International journal of environmental research and public health. 2022;19(12).

69. Morton S, C F, Jepson R, DH S, Sivaramakrishnan D, Niven A. What works to reduce sedentary behavior in the office, and could these intervention components transfer to the home working environment?: A rapid review and transferability appraisal. Frontieres in sports and active living 2022;4:954639.

70. Morton L, Stelfox K, Beasley M, Jones GT, Macfarlane GJ, Walker-Bone K, et al. Enabling work participation for people with musculoskeletal conditions: lessons from work changes imposed by COVID-19: a mixed-method study. BMJ Open. 2022;12(4):e057919.

71. Nagata S, Adachi HM, Hanibuchi T, Amagasa S, Inoue S, Nakaya T. Relationships among changes in walking and sedentary behaviors, individual attributes, changes in work situation, and anxiety during the COVID-19 pandemic in Japan. Preventive medicine reports. 2021;24:101640.

72. Niu Q, Nagata T, Fukutani N, Tezuka M, Shimoura K, Nagai-Tanima M, et al. Health effects of immediate telework introduction during the COVID-19 era in Japan: A cross-sectional study. PloS one. 2021;16(10):e0256530.

73. Niven A, Baker G, Almeida EC, Fawkner SG, Jepson R, Manner J, et al. "Are We Working (Too) Comfortably?": Understanding the Nature of and Factors Associated with Sedentary Behaviour When Working in the Home Environment. Occupational health science. 2023;7(1):71-88.

74. Olsen H, Brown W, Burton N. Workplace flexibility: Does policy influence physical activity and sedentary behaviour in employees? Journal of Science and Medicine in Sport. 2014;18:e7.

75. Olsen HM, Brown WJ, Kolbe-Alexander T, Burton NW. A Brief Self-Directed Intervention to Reduce Office Employees' Sedentary Behavior in a Flexible Workplace. Journal of occupational and environmental medicine. 2018;60(10):954-9.

76. Papalia GF, Petrucci G, Russo F, Ambrosio L, Vadalà G, Iavicoli S, et al. COVID-19 Pandemic Increases the Impact of Low Back Pain: A Systematic Review and Metanalysis. International journal of environmental research and public health. 2022;19(8).

77. Petroni ML, Caretto A, Pintus S, Morabito S, Zuliani P, Di Carlo MS, et al. Factors predicting weight gain during Covid-19 lockdown in patients with obesity: A national survey. Clinical Nutrition ESPEN. 2020;40:659.

78. Pino-Casal V, De-Pedro-jiménez D. CHANGES IN SEDENTARY AND PHYSICAL ACTIVITY HABITS OF WORKERS WHO SWITCHED TO TELEWORKING DURING THE COVID PANDEMIC19. PLos one. 2021;30(4):436-42.

79. Radulović AH, Žaja R, Milošević M, Radulović B, Luketić I, Božić T. Work from home and musculoskeletal pain in telecommunications workers during COVID-19 pandemic: a pilot study. Archives of Industrial Hygiene and Toxikology. 2021;72(3):232-9.

80. Rapisarda V, Loreto C, De Angelis L, Simoncelli G, C L, Resina R, et al. Home Working and Physical Activity during SARS-CoV-2 Pandemic: A Longitudinal Cohort Study. International journal of environmental research and public health. 2021;18(24).

81. Roberts H, van Lissa C, Helbich M. Perceived neighbourhood characteristics and depressive symptoms: Potential mediators and the moderating role of employment status. Social science & medicine (1982). 2021;268:113533.

82. Schifferli I, Orellana JJ, Pedraza L. [Influence of Remote Work Imposed by COVID-19 Lockdowns on Weight Gain in Adults Across 21 Ibero-American Countries]. Rev Med Chil. 2025;153(1):1-10.

83. Scoditti E, Bodini A, Sabina S, Leo CG, Mincarone P, Rissotto A, et al. Effects of working from home on lifestyle behaviors and mental health during the COVID-19 pandemic: A survey study. PLoS One. 2024;19(4):e0300812.

84. Namli Seker A, Arman N. Comparison of the Effects of Two Different Exercise Programs on Lower Limb Functions, Posture, and Physical Activity in Office Workers Working at Home and in Office Alternately: A Randomized Controlled Trial. American journal of physical medicine & rehabilitation. 2024;103(2):134-42.

85. Shifrin NV, Michel JS. Flexible work arrangements and employee health: A meta-analytic review. Work & Stress. 2022;36(1):60-85.

86. Silvestre BA, Miotto LP, Gramani-Say K, Barbosa MH, Hortense P. Chronic pain and associated factors in remote work during the COVID-19 pandemic in Brazil. Brazilian Journal of Nursing. 2023;76Suppl 1(Suppl 1):e20230012.

87. Šmite D, Moe NB, Klotins E, Gonzalez-Huerta J. From forced Working-From-Home to voluntary working-from-anywhere: Two revolutions in telework. The Journal of systems and software. 2023;195:111509.

88. Somasundram KG, Hackney A, Yung M, Du B, Oakman J, Nowrouzi-Kia B, et al. Mental and physical health and well-being of canadian employees who were working from home during the COVID-19 pandemic. BMC public health. 2022;22(1):1987.

89. Sparks JR, Kebbe M, Flanagan EW, Beyl RA, Altazan AD, Yang S, et al. Impact of COVID-19 Stay-at-Home Orders on Health Behaviors and Anxiety in Black and White Americans. Journal of racial and ethnic health disparities. 2021:1-5.

90. Tazegül G, Aydin V, Aksuna GK, Yücel R, Dik B, Aksoy S, et al. An assessment of cardiovascular health risks in people working from home during the pandemic period: PANDEV-KALP study. Anatololian journal of cardiology. 2022;26:S164-S5.

91. Tobin S, Halliday T, Burns R, Qeadan F, Glazer Baron K. Factors Influencing Physical Activity During The COVID-19 Pandemic In Adults From Utah. Medicine & Science in Sports & Exercise. 2021;53:189-90.

92. Valecha RL. Dual Role Doubles Health Risk for Women. Indian Journal of Occupational and Environmental Medicine. 2021;25(1):48.

93. Wakaizumi K, Yamada K, Shimazu A, Tabuchi T. Sitting for long periods is associated with impaired work performance during the COVID-19 pandemic. Journal of occupational health. 2021;63(1):e12258.

94. Weale V, Lambert KA, Graham M, Stuckey R, Oakman J. Do work-family conflict or family-work conflict mediate relationships between work-related hazards and stress and pain? American journal of industrial medicine. 2023;66(9):780-93.

95. Werneck AO, Silva DR, Malta DC, Souza-Júnior PRB, Azevedo LO, Barros MBA, et al. Changes in the clustering of unhealthy movement behaviors during the COVID-19 quarantine and the association with mental health indicators among Brazilian adults. Translational behavioral medicine. 2021;11(2):323-31.

96. Wiezer N, Hengel KO, Zoomer T, Hooftman W. Working from home during the COVID -19 pandemic in the Netherlands. Safety and Health at Work. 2022;13:S78.

97. Wilke J, Hollander K, Mohr L, Edouard P, Fossati C, González-Gross M, et al. Drastic Reductions in Mental Well-Being Observed Globally During the COVID-19 Pandemic: Results From the ASAP Survey. Frontiers in Medicine. 2021;8.

98. Xiao Y, Becerik-Gerber B, Lucas G, Roll SC. Impacts of Working From Home During COVID-19 Pandemic on Physical and Mental Well-Being of Office Workstation Users. Journal of occupational and environmental medicine. 2021;63(3):181-90.

99. Yang GY, Lin XL, Fang AP, Zhu HL. Eating Habits and Lifestyles during the Initial Stage of the COVID-19 Lockdown in China: A Cross-Sectional Study. Nutrients. 2021;13(3).

100. Yan Z, Xiao S, Zhao L. Physical Activity, Type Of Workplaces, And Mood Disturbance Among Chinese Adults During Covid-19. Medicine & Science in Sports & Exercise. 2021;53:309-.

101. Yoshimoto T, Fujii T, Oka H, Kasahara S, Kawamata K, Matsudaira K. Pain Status and Its Association with Physical Activity, Psychological Stress, and Telework among Japanese Workers with Pain during the COVID-19 Pandemic. International journal of environmental research and public health. 2021;18(11).

102. Zhou M, Gebreslassie M, Ponce de Leon A, Tynelius P, Ahlqvist VH, Dahlen M, et al. The influence of the COVID-19 pandemic on physical activity in Stockholm County - Evidence from time series models of smartphone measured daily steps data spanning over 3 years. Preventive Medicine. 2024;183.
